# Author Correction: Polyrotaxane-based supramolecular theranostics

**DOI:** 10.1038/s41467-026-69976-y

**Published:** 2026-04-02

**Authors:** Guocan Yu, Zhen Yang, Xiao Fu, Bryant C. Yung, Jie Yang, Zhengwei Mao, Li Shao, Bin Hua, Yijing Liu, Fuwu Zhang, Quli Fan, Sheng Wang, Orit Jacobson, Albert Jin, Changyou Gao, Xiaoying Tang, Feihe Huang, Xiaoyuan Chen

**Affiliations:** 1 Laboratory of Molecular Imaging and Nanomedicine, National Institute of Biomedical Imaging and Bioengineering, National Institutes of Health, Bethesda, MD 20892 USA; 2 Key Laboratory for Organic Electronics and Information Displays & Institute of Advanced Materials (IAM), Jiangsu National Synergetic Innovation Centre for Advanced Materials (SICAM), Nanjing University of Posts & Telecommunications, 210023 Nanjing, China; 3 Laboratory of Cellular Imaging and Macromolecular Biophysics, National Institute of Biomedical Imaging and Bioengineering (NIBIB), National Institutes of Health, Bethesda, MD 20892 USA; 4 School of Life Science, Beijing Institute of Technology, 100081 Beijing, China; 5 State Key Laboratory of Chemical Engineering, Centre for Chemistry of High-Performance & Novel Materials, Department of Chemistry, Zhejiang University, 310027 Hangzhou, China; 6 MOE Key Laboratory of Macromolecular Synthesis and Functionalization, Department of Polymer Science and Engineering, Zhejiang University, 310027 Hangzhou, China

Correction to: *Nature Communications* 10.1038/s41467-018-03119-w, published online 22 February 2018

This Article contains an error in Supplementary Fig. 34a, in which a wrong image has been used, and in Supplementary Fig. 41c and d and Supplementary Fig. 42a and b in which a cropped version of the same image was used.

The original Supplementary Fig. 34:
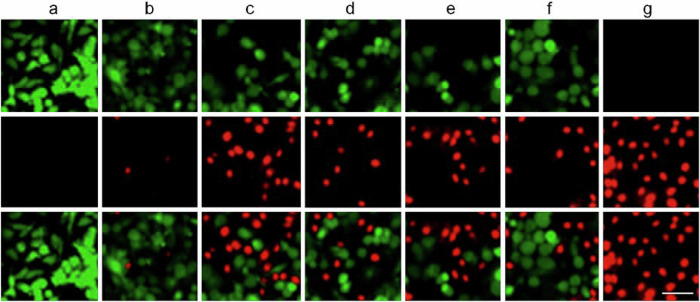


has been replaced with the corrected Figure:
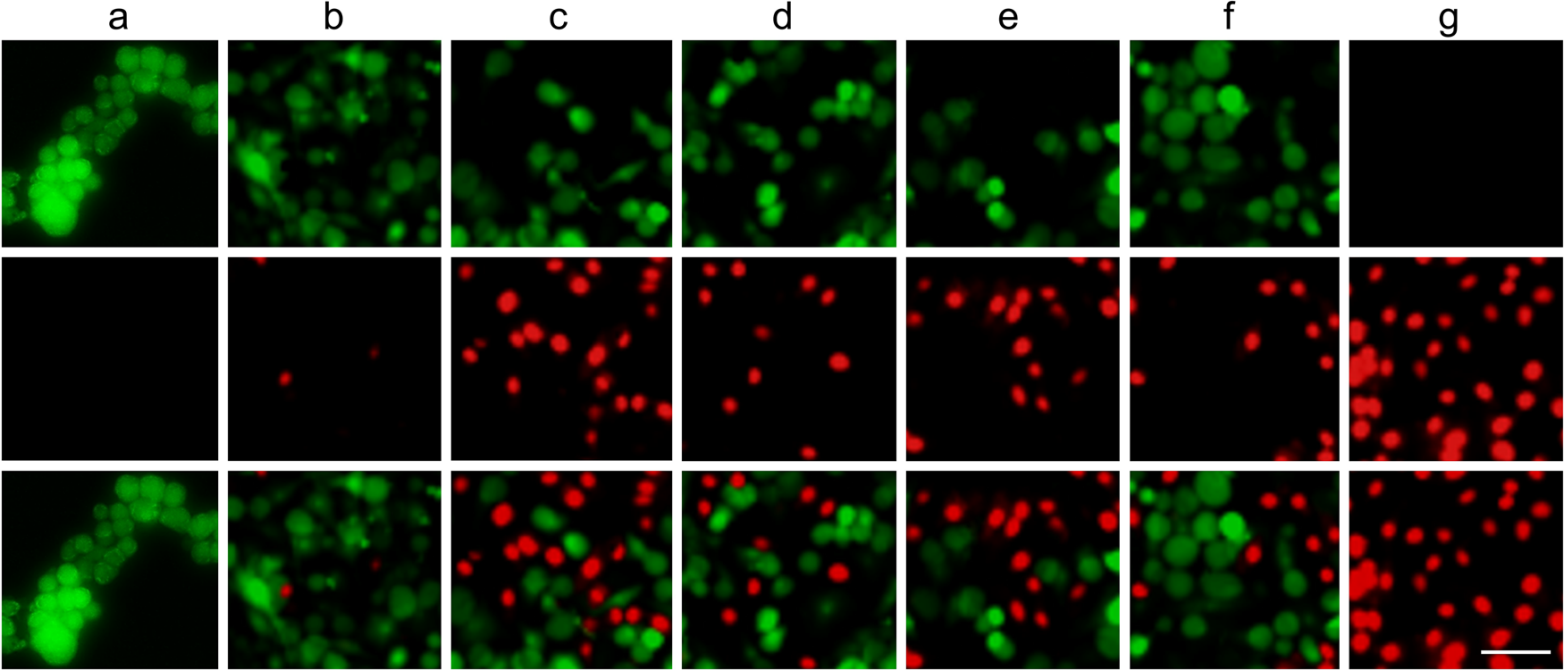


The original Supplementary Fig. 41:
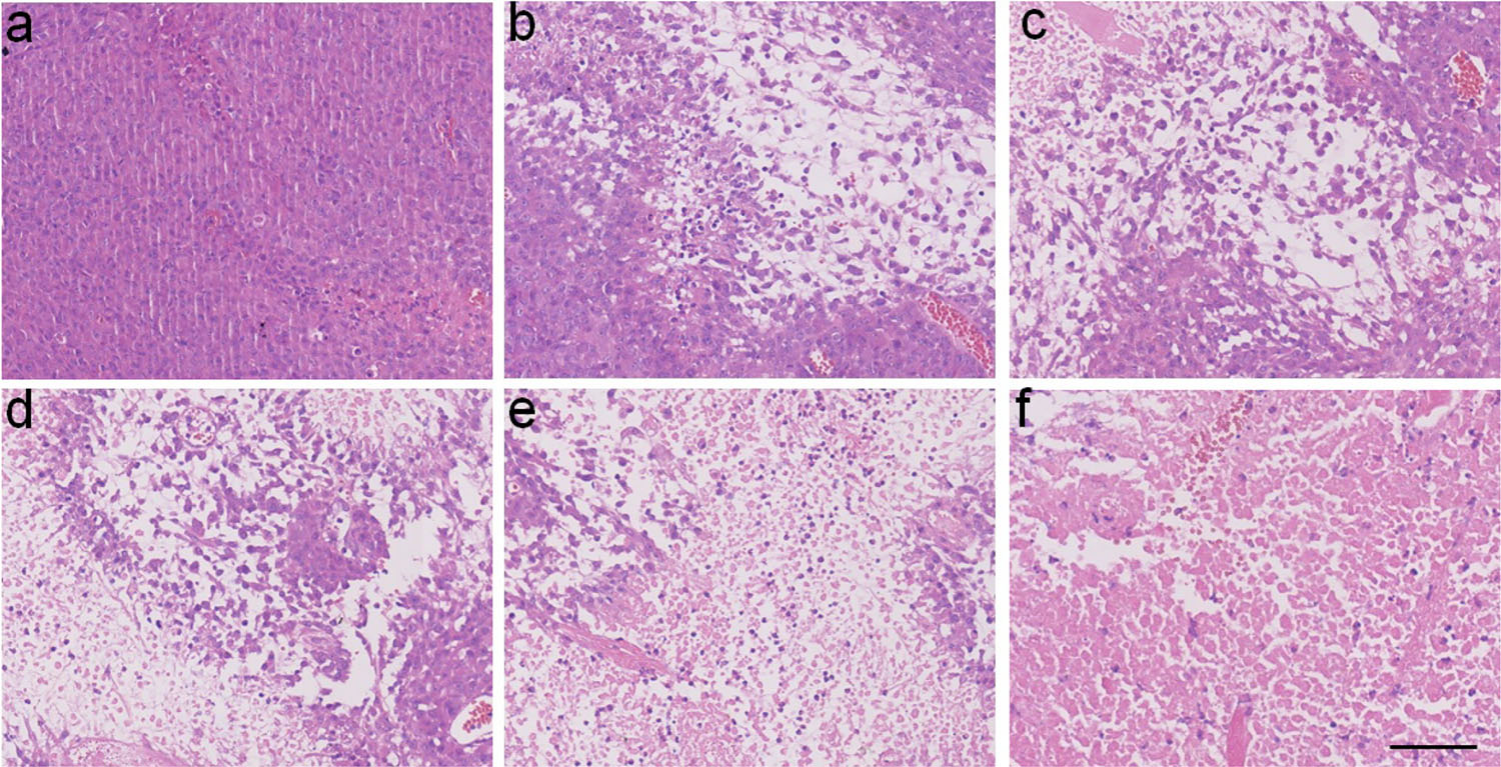


has been replaced with the corrected Figure:
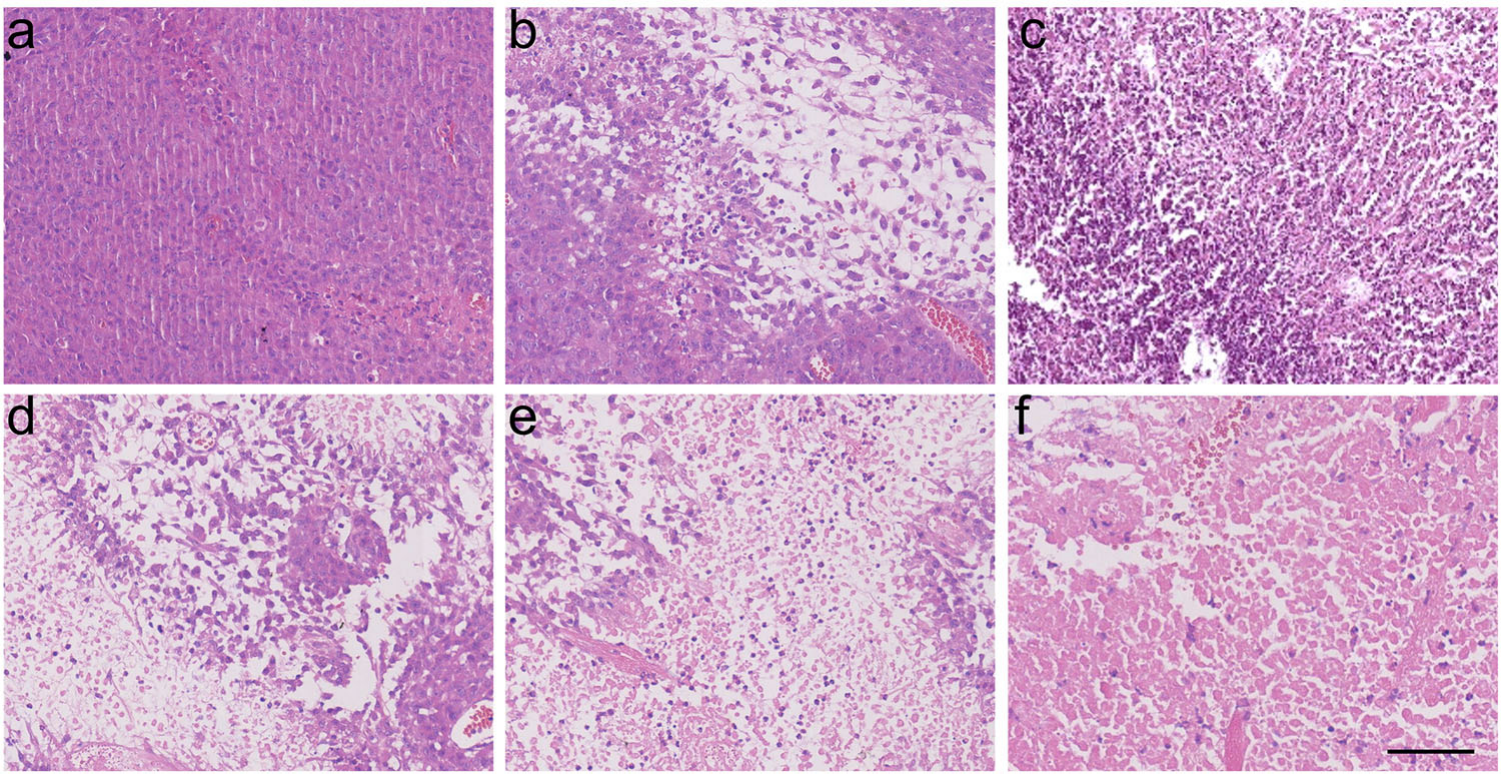


The original Supplementary Fig. 42:
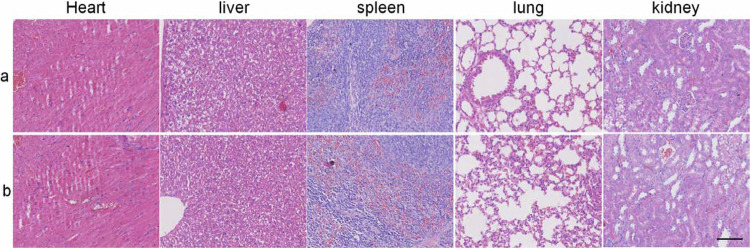


has been replaced with the corrected Figure:
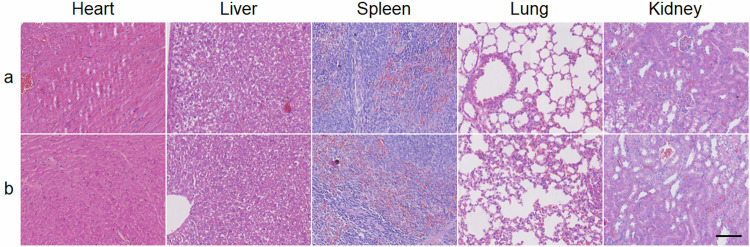


The error has been corrected in the PDF versions of the Supplementary Information.

