# Supplementary material for: Polyrotaxane-based supramolecular theranostics
Source: Nat Commun. 2018 Feb 22;9:766. doi: 10.1038/s41467-018-03119-w (PMC5823937; doi:10.1038/s41467-018-03119-w)
Supplement: Supplementary file 1 — Supplementary Information [file 41467_2018_3119_MOESM1_ESM.pdf]

Polyrotaxane-based supramolecular theranostics

Guocan Yu[1#], Zhen Yang[1,3#], Xiao Fu[4,6#], Bryant C. Yung[1], Jie Yang[5], Zhengwei Mao[2], Li Shao[5], Bin Hua[5], Yijing Liu[1], Fuwu Zhang[1], Quli Fan[3], Sheng Wang[1], Orit Jacobson[1], Albert Jin[4], Changyou Gao[2], Xiaoying Tang[6], Feihe Huang[5], and Xiaoyuan Chen[1]

[1] *Laboratory of Molecular Imaging and Nanomedicine, National Institute of Biomedical Imaging and Bioengineering, National Institutes of Health, Bethesda, Maryland 20892, United States. Email: shawn.chen@nih.gov*

[2] *MOE Key Laboratory of Macromolecular Synthesis and Functionalization, Department of Polymer Science and Engineering, Zhejiang University, Hangzhou 310027, P. R. China. Email: zwmao@zju.edu.cn*

[3] *Key Laboratory for Organic Electronics and Information Displays & Institute of Advanced Materials (IAM), Jiangsu National Synergetic Innovation Center for Advanced Materials (SICAM), Nanjing University of Posts & Telecommunications, Nanjing 210023, P. R. China. Email: iamqlfan@njupt.edu.cn*

[4] *Laboratory of Cellular Imaging and Macromolecular Biophysics, National Institute of Biomedical Imaging and Bioengineering (NIBIB), National Institutes of Health, Bethesda, Maryland 20892, United States*

[5] *State Key Laboratory of Chemical Engineering, Center for Chemistry of High-Performance & Novel Materials, Department of Chemistry, Zhejiang University, Hangzhou 310027, P. R. China. Email: fhuang@zju.edu.cn*

[6] *School of Life Science, Beijing Institute of Technology, Beijing 100081, P. R. China*

[#]These authors contributed equally

Supporting Information (57 pages)

1. *Supplementary Methods* S2
2. *Syntheses and characterizations of* ***PDI-PCL-b-PEG-RGD⊃β-CD-NH₂*** S7
3. *Self-assembly studies* S22
4. *In vitro photothermal and photoacoustic studies* S23
5. *In vitro thermo-chemotherapy investigations of* ***SCNPs****@PTX* S24
6. *In vivo thermo-chemotherapy investigations of* ***SCNPs****@PTX* S30
7. *In vitro and in vivo thermo-chemotherapy investigations of* ***SCNPs****@CPT* S43

Supplementary Methods

1,5,7-triazabicyclo[4.4.0]dec-5-ene (TBD), (3,3′-dithiobis(sulfosuccinimidyl propionate)) (**NHS-SS-NHS**), suberic acid bis(3-sulfo-*N*-hydroxysuccinimide ester) sodium salt (**NHS-CC-NHS**) were purchased from Sigma. **HO-PEG-Mal** (2 kDa) was purchased from Biochempeg. Radio metal [^{64}Cu] was produced by the PET Department, NIH Clinical Center. All reagents were commercially available and used as supplied without further purification. Solvents were either employed as purchased or dried according to procedures described in the literature. ^{1}H NMR spectra were recorded on a Bruker Avance III-300 spectrometer with internal standard TMS. The 2D NOESY NMR spectrum was collected on a Bruker Avance DMX-500 spectrometer. HRMS were obtained on a WATERS GCT Premier mass spectrometer. The melting points were collected on a SHPSIC WRS-2 automatic melting point apparatus. The fluorescence experiments were conducted on a RF-5301 spectrofluorophotometer (Shimadzu Corporation, Japan). UV-vis absorption spectra were recorded by using a Shimadzu UV-2501 spectrophotometer. Thermogravimetric analysis (TGA) was performed on a Perkin-Elmer Diamond TG/DTA. Samples were placed in platinum sample pans and heated under a nitrogen atmosphere at a rate of 10 °C min^{-1} to 100 °C and held for 40 min to completely remove residual solvent. Samples were then heated to 800 °C at a rate of 10 °C min^{-1}. Transmission electron microscopy (TEM) investigations were carried out on a Jeol JEM 2010 instrument. Gel permeation chromatography (GPC) was conducted on a Waters Chromatography, Inc. (Milford, MA) system using DMF containing 0.05 M LiBr as eluent (flow rate set to 1.00 mL min^{-1}) equipped with an isocratic pump model 1515, a differential refractometer model 1515, and a three-column set of Styragel HR 4 5 μm DMF (300 × 7.5 mm), Styragel HR 4E 5 μm DMF (300 × 7.5 mm), and Styragel HR 2 5 μm DMF (300 × 7.5 mm). Polymer solutions were prepared at a concentration of *ca.* 3 mg mL^{-1} and the injection volume was 200 μL. Data collection and analysis were performed using Empower 2 v. 6.10.01.00 software (Waters, Inc.). The system was calibrated with polystyrene standards (Polymer Laboratories, Amherst, MA) ranging from 615 to 442,800 Da.

Critical Aggregation Concentration (CAC) Determination. The CAC values of **PDI-PCL-*b*-PEG-RGD** and **PDI-PCL-*b*-PEG-RGD⊃*β*-CD-NH$_2$** in water were determined by using pyrene as a fluorescent probe. The sample solutions with different concentrations were prepared in the presence of pyrene (2 × 10^{-7} mol L^{-1}). Then, all the sample solutions were stirred at room temperature for 24 h to allow for equilibration of pyrene into the self-assemblies. The excitation wavelength was kept at 334 nm, while the emission spectra were recorded from 360 to 460 nm. By plotting the ratios of intensities at 369 and 382 nm against the polymer concentrations in the sample solutions, the CAC values were taken as the intersection of the tangent to the curve at the inflection with tangent through the points at low polymer concentration.

Preparation of Nanoparticles (NPs) and Shell-Crosslinked NPs (SCNPs). PDI-PCL-*b*-PEG-RGD⊃*β*-CD-NH$_2$ (2.00 mg) was dissolved in DMF (3 mL) in a 50 mL round bottom flask and allowed to stir for 1 h at room temperature. To this solution, nanopure water (10 mL) was added dropwise *via* a syringe pump over a period of 6 h. The mixture was allowed to stir overnight at room temperature. DMF was eliminated by dialysis (MWCO 3 kDa) against deionized water to afford non-crosslinking NPs. *In situ* crosslinking was performed by the addition of a stock solution of **NHS-SS-NHS** (250 μg) into the solution of the NPs. The solution was allowed to stir for an additional 24 h at room temperature. NHS byproduct and unreacted **NHS-SS-NHS** were eliminated by dialysis (MWCO 3 kDa) against deionized water to afford **SCNPs**.

TEM and DLS Studies. The nanostructures of the self-assemblies were revealed using TEM. TEM samples were prepared by drop-coating a solution onto a carbon-coated copper grid. The corresponding solution was left to stand overnight and the insoluble precipitate was eliminated by using a microporous membrane before DLS tests. Dynamic light scattering (DLS) measurements were carried out using a 200 mW polarized laser source Nd:YAG (λ = 532 nm). The polarized scattered light was collected at 90° in a self-beating mode with a Hamamatsu R942/02 photomultiplier. The signals were sent to a Malvern 4700 submicrometer particle analyzer system.

Drug Release of from SCNPs. The release profiles were studied by monitoring the increase of PTX or CPT concentration in culture solution over time by high-performance liquid chromatography (HPLC) using a UV detector. In a typical procedure, drug-loaded NPs or drug-loaded **SCNPs** were diluted with phosphate buffered saline (PBS), yielding a concentration of 500 μg mL^{-1}. A portion of the solution (3.0 mL) was transferred into a presoaked dialysis cassette (Slide-A-Lyzer, MWCO 10 kDa, Pierce Biotechnology, Rockford IL). The cassette was allowed to stir in a beaker containing 500 mL PBS (pH 7.4 at 37 °C) in the absence or presence of GSH (10.0 mM) with/without laser irradiation (671 nm, 0.5 W cm^{-2}). At designated time intervals, 50 mL of medium was taken out from the culture solution and concentrated by vacuum rotary. The concentration of the released drug was analyzed by HPLC based on the standard curve calibrated with a drug solution with a known concentration. The release was evaluated in triplicate.

Calculation of the Photothermal Conversion Efficiency. The photothermal conversion efficiency (η) was determined according to the reported method.[S1] The temperature change of the aqueous dispersion was recorded as a function of time under continuous irradiation of the 671 nm laser with a power density of 0.5 W cm^{-2} until the solution reached a steady-state temperature. The η value was calculated using an equation as follows:

$\eta = [hS(T_{max} - T_{surr}) - Q_{dis}]/I(1 - 10^{-A})$

where h is the heat transfer coefficient, S is the surface area of the container, T_{max} is the equilibrium temperature, T_{Surr} is ambient temperature of the surroundings, Q_{dis} expresses the heat dissipation from the light absorbed by the quartz sample cell, I is incident laser power (0.5 W cm^{-2}), and A is the absorbance at 671 nm. The value of hS is derived according to the equation as follows:

$\tau = mC/hS$

where τ is the sample system time constant, m and C are the mass and heat capacity (4.2 J g^{-1}) of deionized water used as the solvent, respectively. The Q_{dis} was measured independently using a quartz cuvette cell containing pure water without **SCNPs**.

AFM Tip Modification. In order to reduce the indentation depth of the AFM tip during the experiments, a silica bead with diameter 9.6 μm was manually attached to the top of the cantilever tip. Using navigation functions in the Bio-AFM, the AFM tip was dipped in UV-curing glue, carefully lowered onto the center of a pre-selected silica sphere bead. The tip was then withdrawn, and given that the bead was visibly attached to the end of the tip, ultraviolet light was turned on for 30 minutes to set the glue.

AFM Force Volume Measurement. AFM measurements were taken at 37 ^{o}C using a Bioscope Catalyst AFM device (Bruker Santa Barbara, CA) with a combined optical microscope (IX71, Olympus, Japan). This combination allowed lateral positioning of the AFM tip in the region of interest cells with a micrometer precision. The system was equipped with a piezo-electric AFM scanner to drive the movement of the AFM probe in the z-axis within a range of 25 μm. The maximum x-y scan range was 177x177 $μm^2$. AFM mechanical measurements were collected using Nanoscope software (Bruker, Santa Barbara, CA) in Force Volume mode using a 9.6 μm-wide glass bead modified MSCT-D tip at 37 ^{o}C. In order to obtain quantitative data, we used the same tip for all the force measurement. The spring constant of the cantilever was experimentally determined by the thermal tuning method. The deflection sensitivity of the cantilever was calculated from the slope of the force-displacement curve, obtained from the ramp curves on a hard surface. To get quantitative data, we used consistent parameters: ramp rate of 1.0 Hz, indentation of 3 μm, and imaging force of 1.5 nN. For each dish of cells, we acquired multiple force-displacement (F-z) curves (where $F = kd$, d is the deflection of the cantilever). The tip moved across the defined scan area with 32*32 or 64*64 data points.

AFM Data Analysis. The real-time monitoring of mechanical properties were analysed based on force displacement curves by Nanoscope Analysis Software (Ver. 1.7, Bruker, Santa Barbara, CA). Each experimental F-z curve was fitted to the sphere indenter Hertz model:

$$F = \frac{4}{3}\frac{E}{1-\mu^2}\sqrt{R\delta_0^3}$$

where E is the Young's modulus, μ is Poisson's ratio, R is the radius of the sphere probe (4800 nm), and δ is the indentation depth of the sample. Poisson's ratio of incompressible composites is 0.3 here for cells.

The Hertz model was developed for a known geometry as a sphere over either a plane or hemisphere. Herein, we processed on the force displacement curves from the top area of the cell as the force displacement curves on the cell. The top area was defined by the real height of the cell. On each cell, 5–20 force displacement curves were picked randomly as representative of the mechanical properties of the cell. In total, approximately 500–700 force displacement curves were analyzed for each case. We used the retrace force displacement curves and the fitting boundaries (10–30%) of the baseline to trigger force for acquiring the Young's modulus values. We only included data points having an R^2 value predominantly between 0.8 and 0.996 and disregarded a small fraction of force displacement curves that did not show a designed trigger force of typically 1.5 nN.

***In Vitro* Cellular Internalization of SCNPs.** HeLa and A549 cells were seeded at a density of 3.00×10^5 cells per well in 12-well cell culture plates. The cells were left to grow for 24 h in DMEM media containing 10% FBS at 37 °C in 5% CO_2 atmosphere. After 24 h, **SCNPs** at different concentrations were added to the wells and the cells were incubated for 24 h. Following incubation, cells were rinsed twice with PBS to remove residual **SCNPs**. Cells were lysed and the amount of **SCNPs** endocytosed by the cells was determined by UV-vis spectroscopy (based on the characteristic absorbance of PDI at 671 nm). Cells pre-treated with free cRGDfK (20 μM) for 30 min were used as a control to confirm the targeting ability of **SCNPs**.

***In Vitro* Cell Accumulation of Drug-Loaded SCNPs by Confocal Laser Scanning Microscopy (CLSM).** HeLa and A549 cells were treated with Cy5.5 labeled **SCNPs**@PTX or **SCNPs**@CPT in the culture medium at 37 °C for 8 h. The cells were washed three times with PBS and fixed with fresh 4.0% formaldehyde at room temperature for 15 min. For the cells treated with Cy5.5 labeled **SCNPs**@PTX, the nucleus were stained with DAPI and β-actins were stained with phalloidin-FITC. For the cells treated with **SCNPs**@CPT, the nucleus were stained with DOX·HCl (0.10 μM) and the lysosomes were stained with Lyso tracker Green. The images were taken using a LSM-510 confocal laser scanning microscope (Zeiss, Germany) (100 × oil objective, 405/488 nm excitation). Cells pre-treated with free cRGDfK (20 μM) for 30 min were used as a control to confirm the targeting ability of **SCNPs**.

***Ex Vivo* Histological Staining.** HeLa tumour-bearing mice or A549 tumour-bearing mice were sacrificed

at different days. Then, tumours and major organs were collected, and tissues were fixed with *Z*-fix solution and stored at room temperature. For the mice treated with high dosage **SCNPs**@PTX (or **SCNPs**@CPT) followed by laser irradiation, the tumours and major organs were collected after irradiation for 24 h, because the tumours were totally suppressed at 3 day post irradiation. The blocks were sectioned transversely at a thickness of 4 μm. H&E staining was examined by a BX41 bright field microscopy (Olympus).

Statistical Analysis. Data are reported as mean ± SD. The differences among groups were determined using one-way ANOVA analysis followed by Tukey' s post-test: *$P < 0.05$, **$P < 0.01$, ***$P < 0.001$.

Syntheses and characterizations of ***PDI-PCL-b-PEG-RGD⊐β-CD-NH₂***

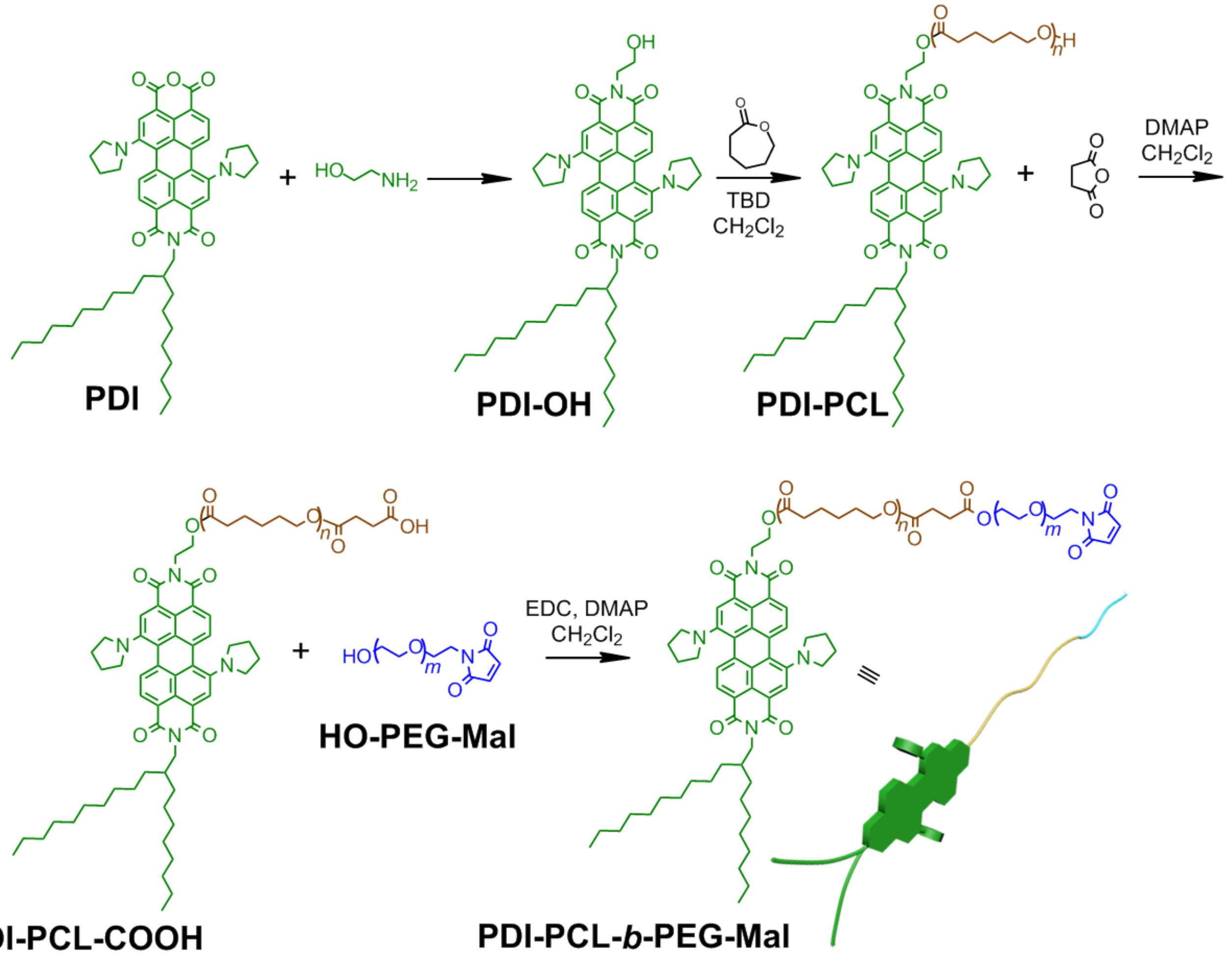

Supplementary Figure 1. Synthetic scheme of **PDI-PCL-*b*-PEG-Mal**, starting from PDI.

Synthesis of **PDI-OH**: Compound PDI[S2] (809 mg, 1.00 mmol), NMP (60 mL), propanoic acid (20 mL) and 2-aminoethanol (610 mL, 10 mmol) were mixed in a 250 mL three-neck flask, The mixture was degassed and refluxed at 150 ºC for 24 h under N_2. After being cooled to room temperature, the mixture was poured into 300 mL methanol and stored at temperature of –10 ºC overnight, the formed precipitate was collected by filtration and washed with cold methanol. The precipitate was purified on a silica gel column using ethyl acetate/CH_2Cl_2 (1:2) as eluent to give **PDI-OH** as a dark green solid (750 mg, 88%). The ^{1}H NMR spectrum of **PDI-OH** is shown in Supplementary Fig. 1. ^{1}H NMR (300 MHz, chloroform-*d*, room temperature) δ (ppm): 8.50–8.42 (m, 4H), 7.74 (d, *J* = 9 Hz, 1H), 7.67 (d, *J* = 6 Hz, 1H), 4.53 (t, *J* = 6 Hz, 2H), 4.15 (d, *J* = 3 Hz, 2H), 4.02 (s, 2H), 3.76 (s, 4H), 2.84 (s, 4H), 2.63 (s, 1H), 2.10–2.02 (m, 8H), 1.62–1.51 (m, 9H), 1.27–1.22 (m, 25H), 0.87–0.82 (m, 6H). The ^{13}C NMR spectrum of **3** is shown in Supplementary Fig. 2. ^{13}C NMR (75 MHz, chloroform-*d*, room temperature) δ (ppm): 164.95, 164.88, 164.26, 164.22, 146.38, 146.21, 134.20, 133.47, 129.63, 129.40, 126.82, 126.79, 126.72, 126.69, 123.88, 123.24, 122.12, 121.72, 121.41, 121.26, 120.72, 120.50, 118.97, 118.52, 118.18, 111.35, 77.25, 76.35, 62.33, 61.98, 61.85, 52.19, 52.11, 44.63, 44.61, 43.13, 43.01, 36.79, 31.93, 31.79, 30.16, 29.71, 29.67,

29.61, 29.37, 29.33, 26.64, 25.79, 22.69, 14.13. HRESIMS: *m*/*z* calcd for $[M + H]^+$ $C_{54}H_{69}N_4O_5$, 854.1495, found 854.1505, error 1.1 ppm.

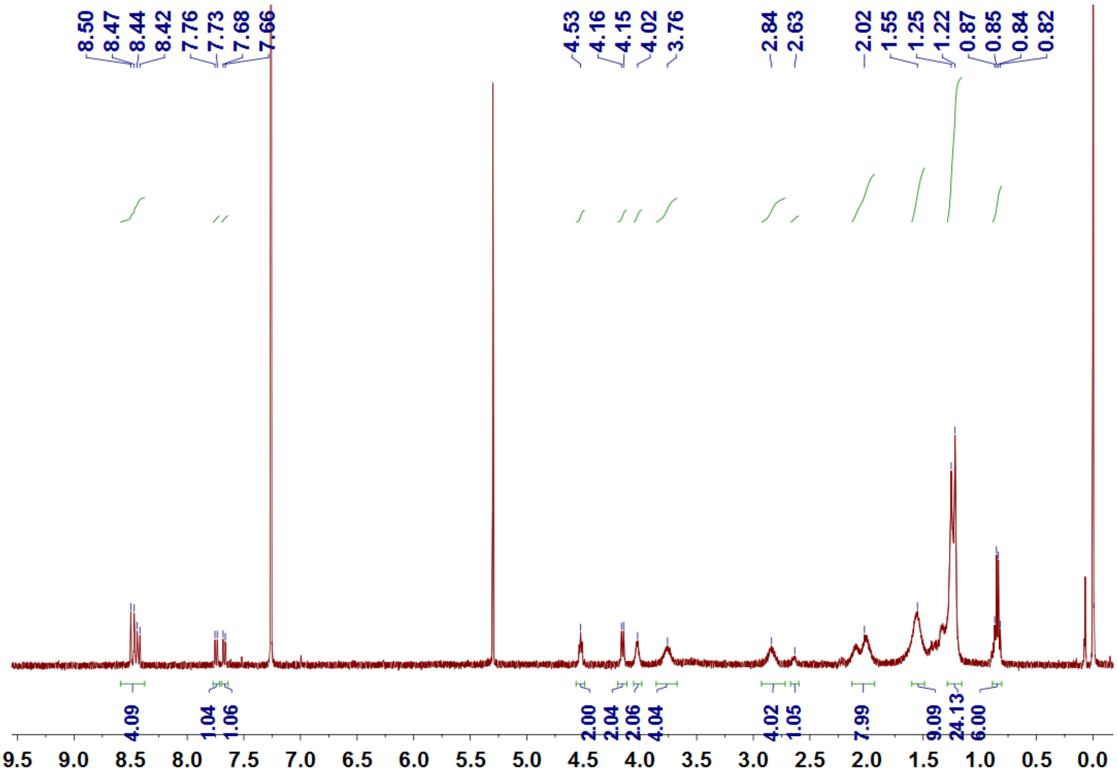

Supplementary Figure 2. ^{1}H NMR spectrum (300 MHz, chloroform-*d*, room temperature) of **PDI-OH**.

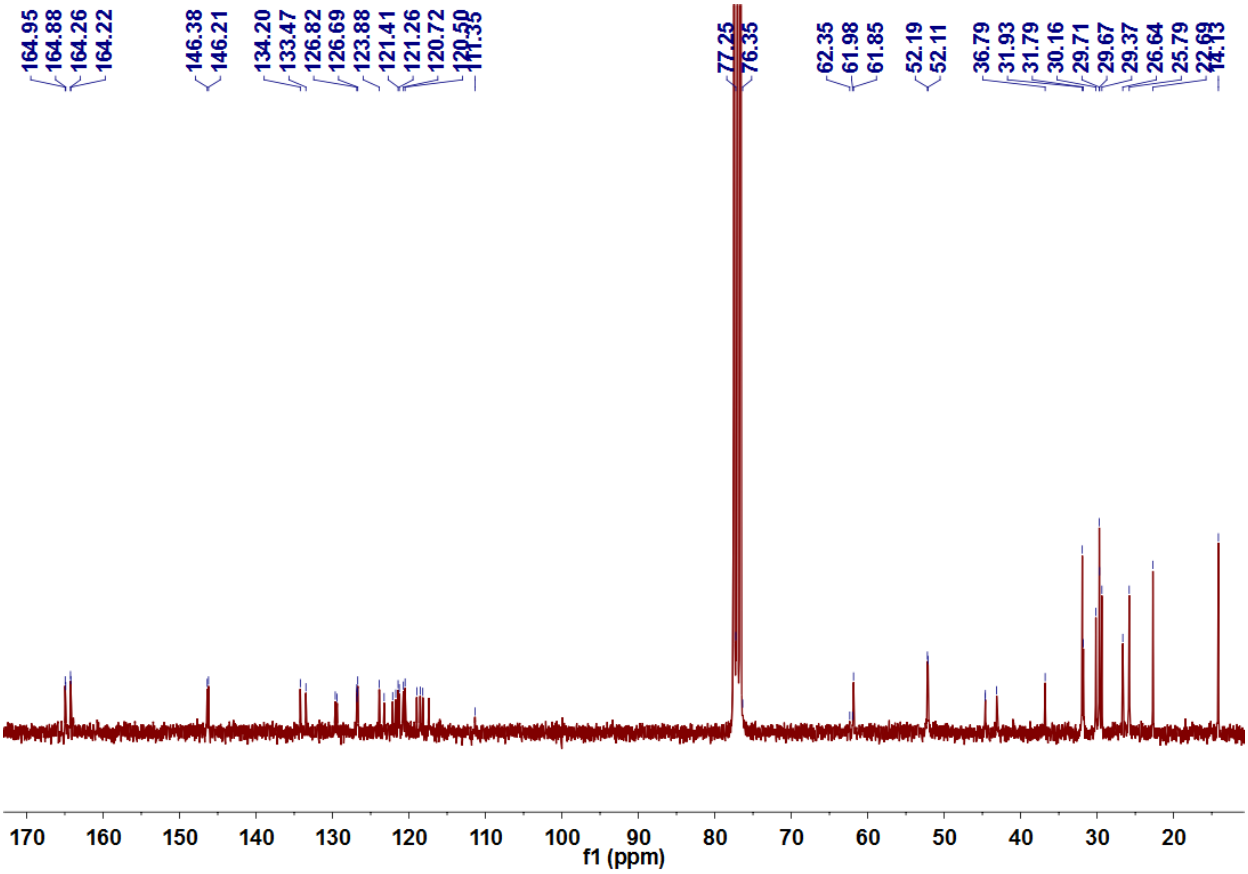

Supplementary Figure 3. ^{13}C NMR spectrum (75 MHz, chloroform-*d*, room temperature) of **PDI-OH**.

Synthesis of **PDI-PCL**: **PDI-OH** (85.4 mg, 0.100 mmol) and *ε*-caprolactone (399 mg, 3.50 mmol) were solubilized in anhydrous CH_2Cl_2 (8 mL). TBD (13.9 mg, 0.100 mmol) was added into the mixture under stirring. After 15 min, the reaction was quenched by adding 1 mL acetic acid. The resulting solution was precipitated into an excess of diethyl ether. After filtration, the sediments were dissolved in CH_2Cl_2 and precipitated into an excess of diethyl ether; the above dissolution-precipitation cycle was repeated three times. After drying in a vacuum oven overnight at room temperature, **PDI-PCL** was obtained as a green solid (460 mg, yield: 95%; M_n (GPC) = 6.47 kDa, M_w/M_n = 1.14). The molecular weight and composition of **PDI-PCL** were determined by ^{1}H NMR spectroscopy (Supplementary Fig. 4) and GPC (Supplementary Fig. 5).

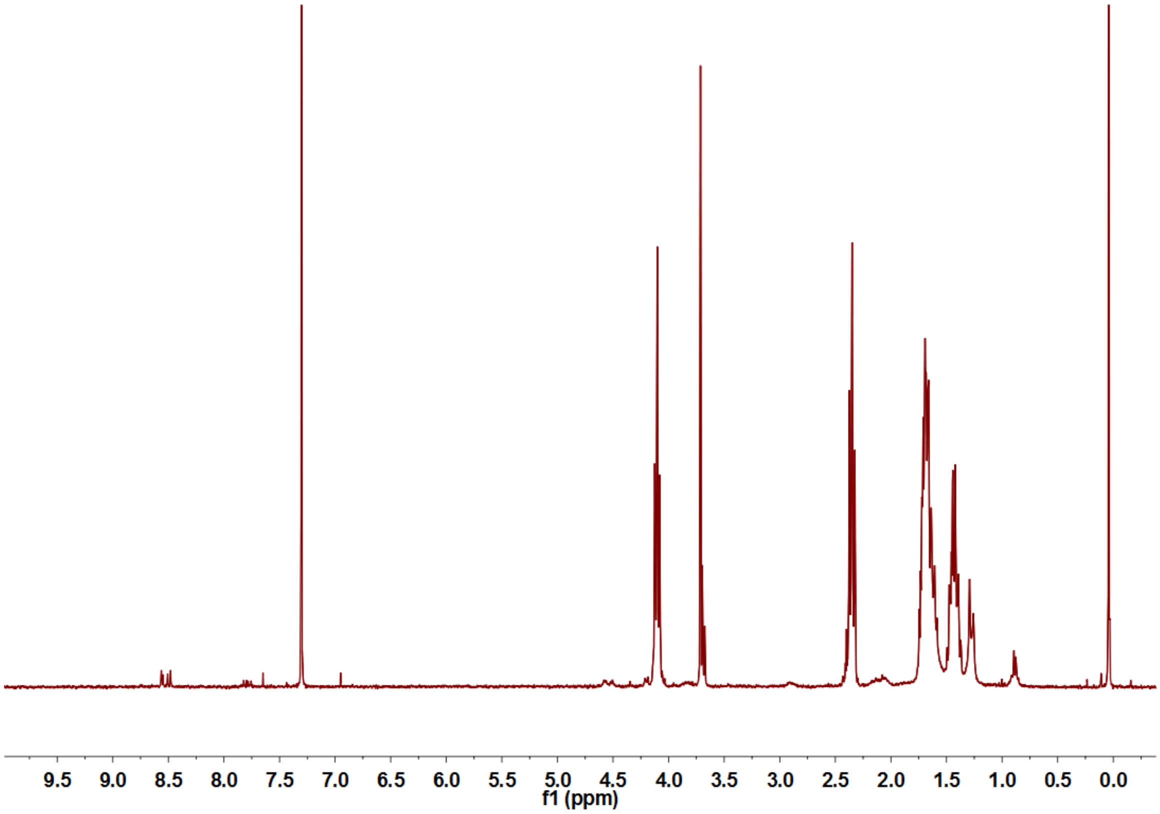

Supplementary Figure 4. ^{1}H NMR spectrum (300 MHz, chloroform-*d*, room temperature) of **PDI-PCL**.

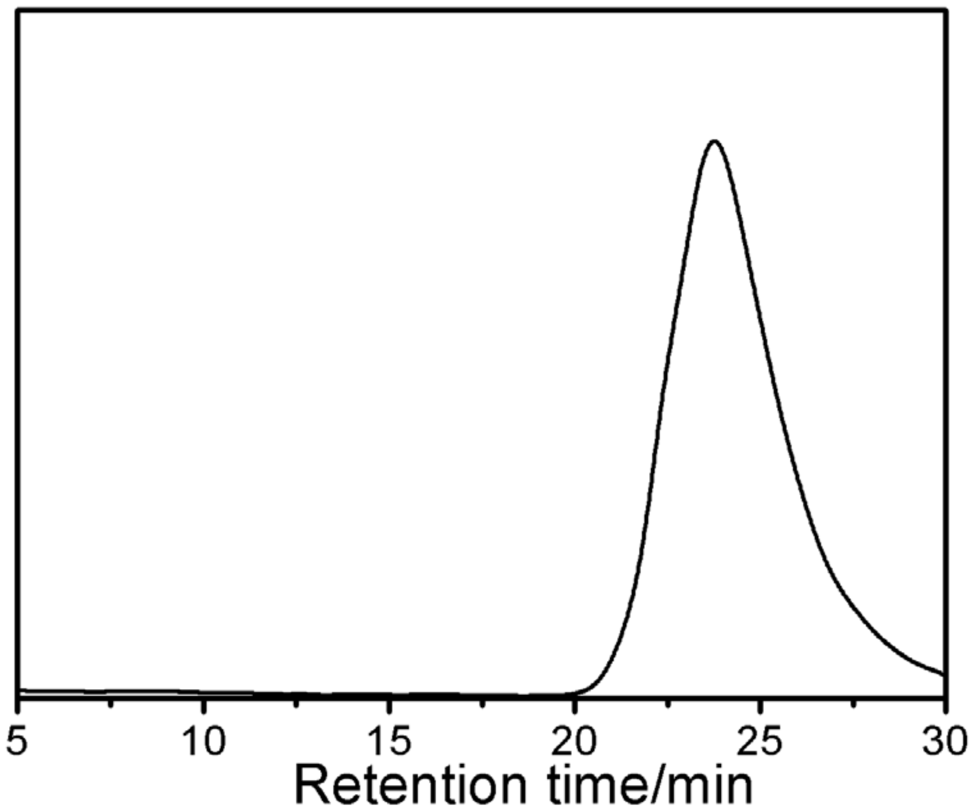

Supplementary Figure 5. GPC curve of **PDI-PCL**.

Synthesis of **PDI-PCL-COOH**: Succinic anhydride (200 mg, 2.00 mmol) was added into the anhydrous CH_2Cl_2 solution containing **PDI-PCL** (440 mg, 1.00 mmol) and DMAP (24.5 mg, 2.00 mmol). After stirring for 12 h, the solution was poured into an excess of diethyl ether. After filtration, the sediments was dissolved in CH_2Cl_2 and precipitated into an excess of diethyl ether; the above dissolution-precipitation cycle was repeated three times. After drying in a vacuum oven overnight at room temperature, **PDI-PCL-COOH** was obtained as a green solid (420 mg, yield: 93%; M_n (GPC) = 6.61 kDa, M_w/M_n = 1.17). The molecular weight and composition of **PDI-PCL-COOH** were determined by ^1H NMR spectroscopy (Supplementary Fig. 6) and GPC (Supplementary Fig. 7).

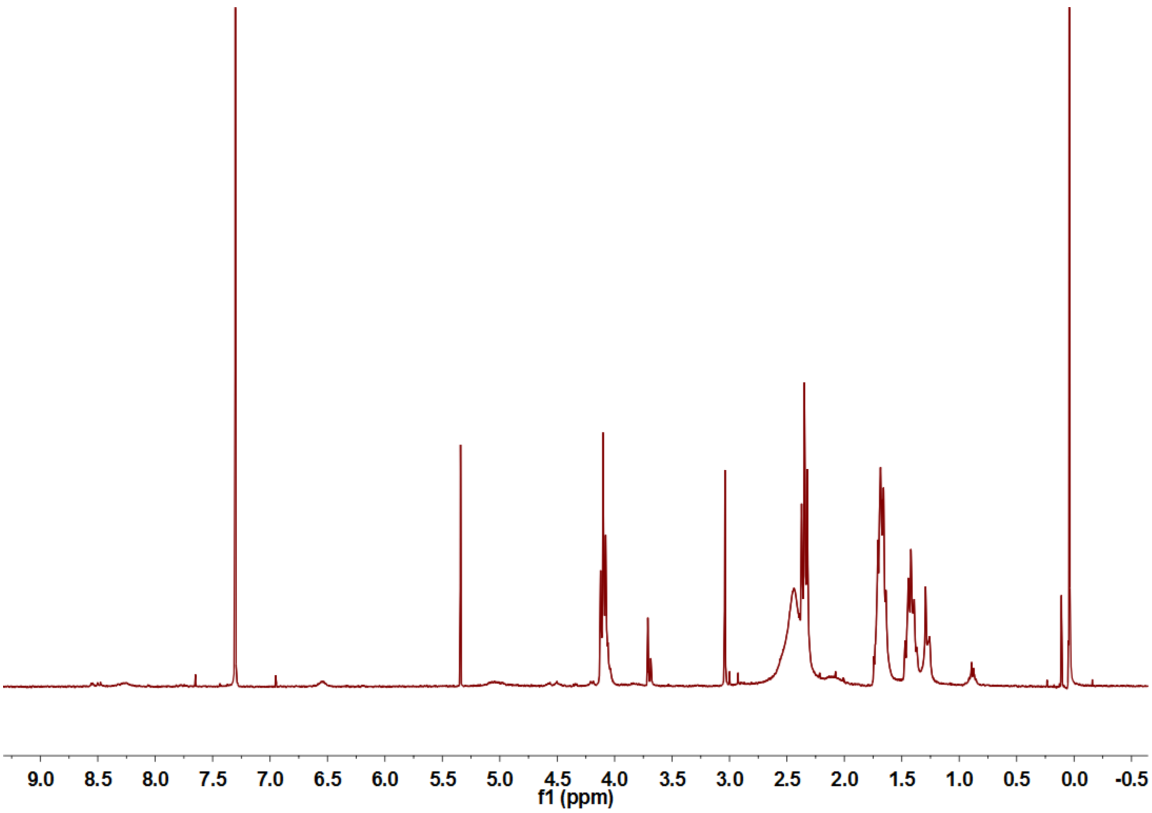

Supplementary Figure 6. ^{1}H NMR spectrum (300 MHz, chloroform-*d*, room temperature) of **PDI-PCL-COOH**.

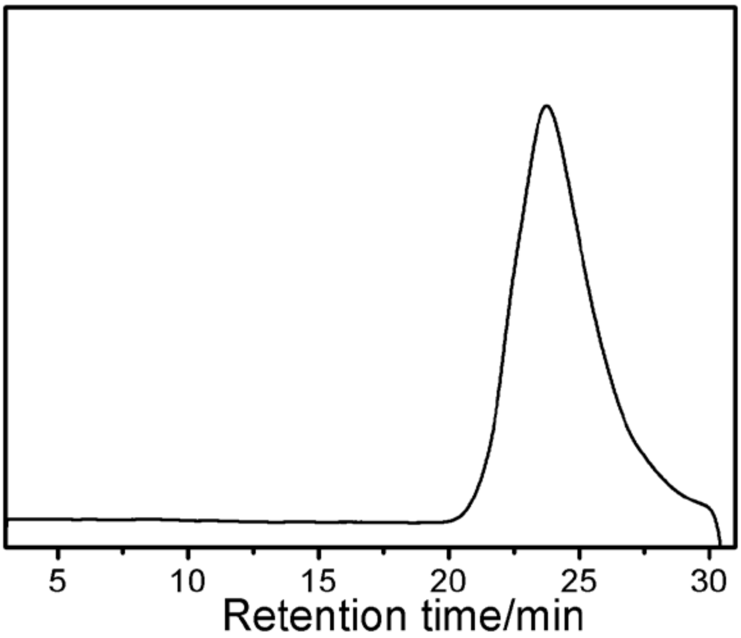

Supplementary Figure 7. GPC curve of **PDI-PCL-COOH**.

Synthesis of **PDI-PCL-*b*-PEG-Mal**: To a solution of **PDI-PCL-COOH** (225 mg, 50.0 μmol) and **HO-PEG-Mal** (210 mg, 100 μmol) in dry CH_2Cl_2 (10 mL), 4-dimethylaminopyridine (DMAP, catalytic amount) and 1-(3'-dimethylaminopropyl)-3-ethylcarbodiimide hydrochloride (EDC, 95.5 mg, 500 μmol) were added. The mixture was stirred overnight at room temperature for 12 h. The solution was evaporated *in vacuo*. The residue was dissolved in THF and the resulting solution was dialyzed overnight against

deionized water using a dialysis bag (MWCO 12 kDa) and then lyophilized to afford **PDI-PCL-*b*-PEG-Mal** as a green solid (286 mg, yield: 88%; M_n (GPC) = 8.65 kDa, M_w/M_n = 1.14). The molecular weight and composition of **PDI-PCL-*b*-PEG-Mal** were determined by ^{1}H NMR spectroscopy (Supplementary Fig. 8) and GPC (Supplementary Fig. 9).

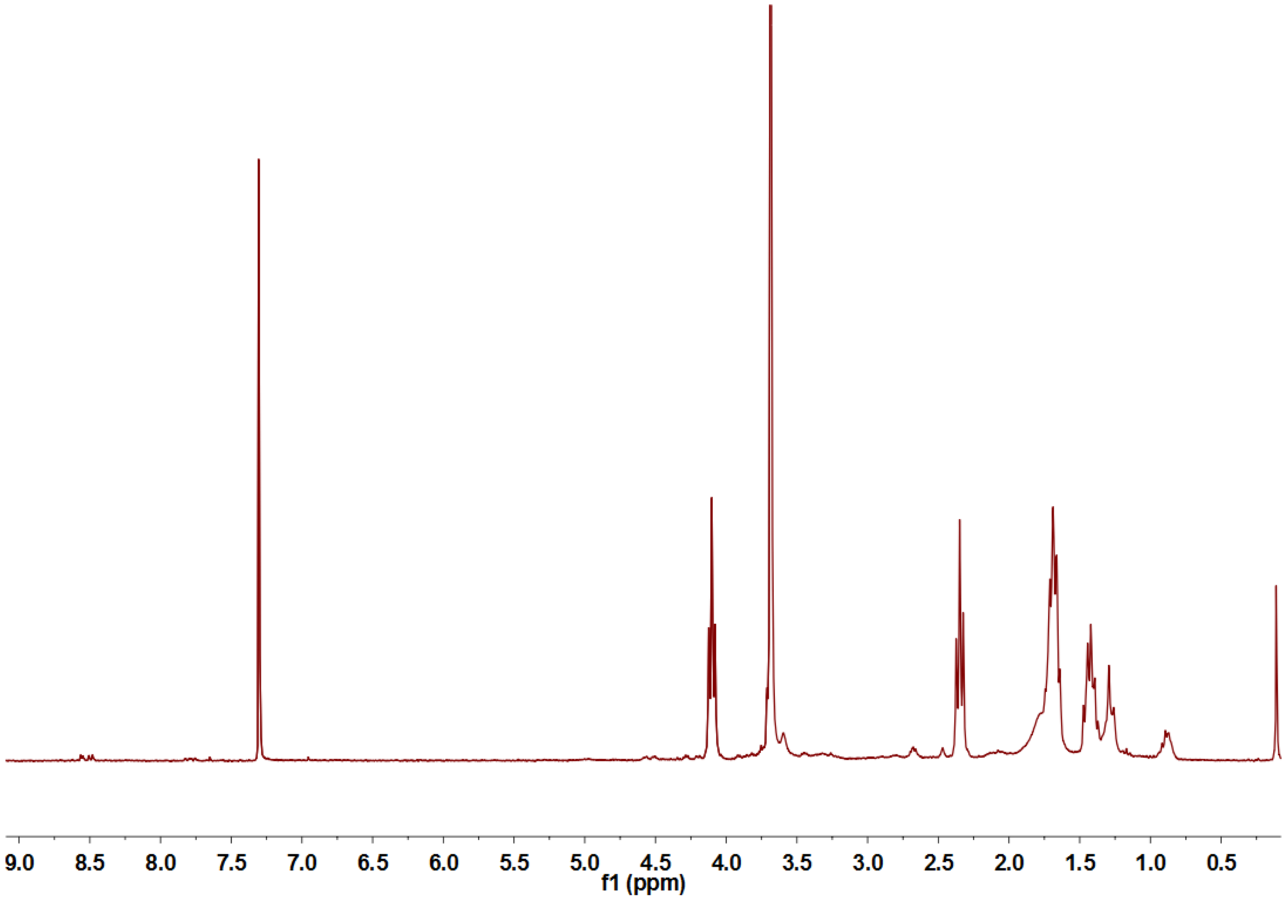

Supplementary Figure 8. ^{1}H NMR spectrum (300 MHz, chloroform-*d*, room temperature) of **PDI-PCL-*b*-PEG-Mal**.

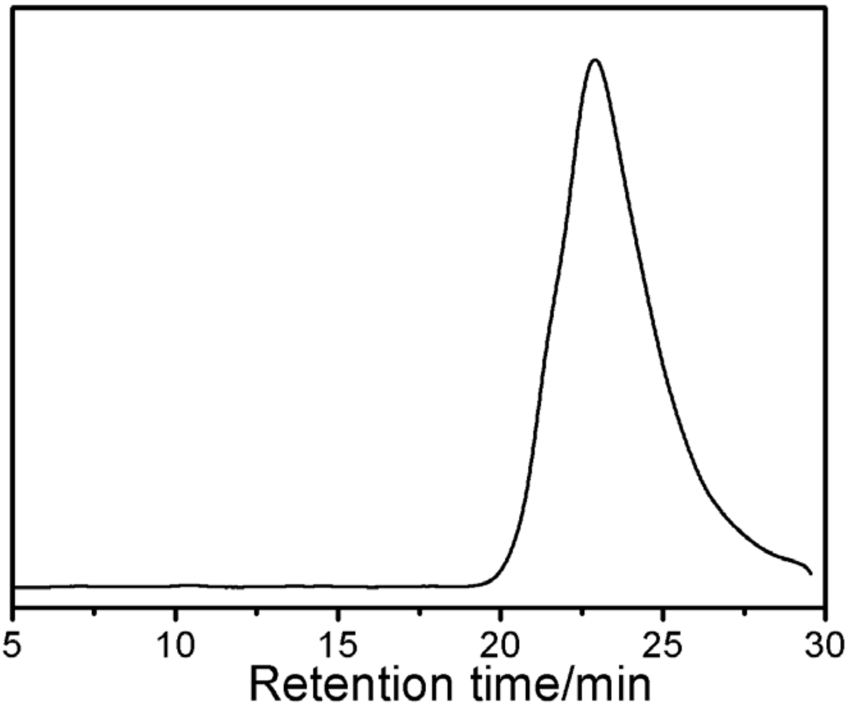

Supplementary Figure 9. GPC curve of **PDI-PCL-*b*-PEG-Mal**.

Synthesis of **PDI-PCL-*b*-PEG-RGD⊃*β*-CD-NH_2: PDI-PCL-*b*-PEG-Mal** (86.5 mg, 10.0 μmol) and ***β*-CD-NH_2**[S3] (235 mg, 200 μmol) were dissolved in the mixture of THF and water (1/1, *v*/*v*). The organic solvent was evaporated by stirring the solution for 12 h at room temperature. Then, cRGDfK-SH (67.5 mg, 100 μmol) was added, and the solution was further stirred for another 24 h. The resulting solution was dialyzed overnight against deionized water using a dialysis bag (MWCO 12 kDa) and then lyophilized to afford **PDI-PCL-*b*-PEG-RGD⊃*β*-CD-NH_2** as a green solid. The molecular weight and composition of **PDI-PCL-*b*-PEG-RGD⊃*β*-CD-NH_2** were determined by ^{1}H NMR spectroscopy (Supplementary Fig. 10) and GPC (Supplementary Fig. 11).

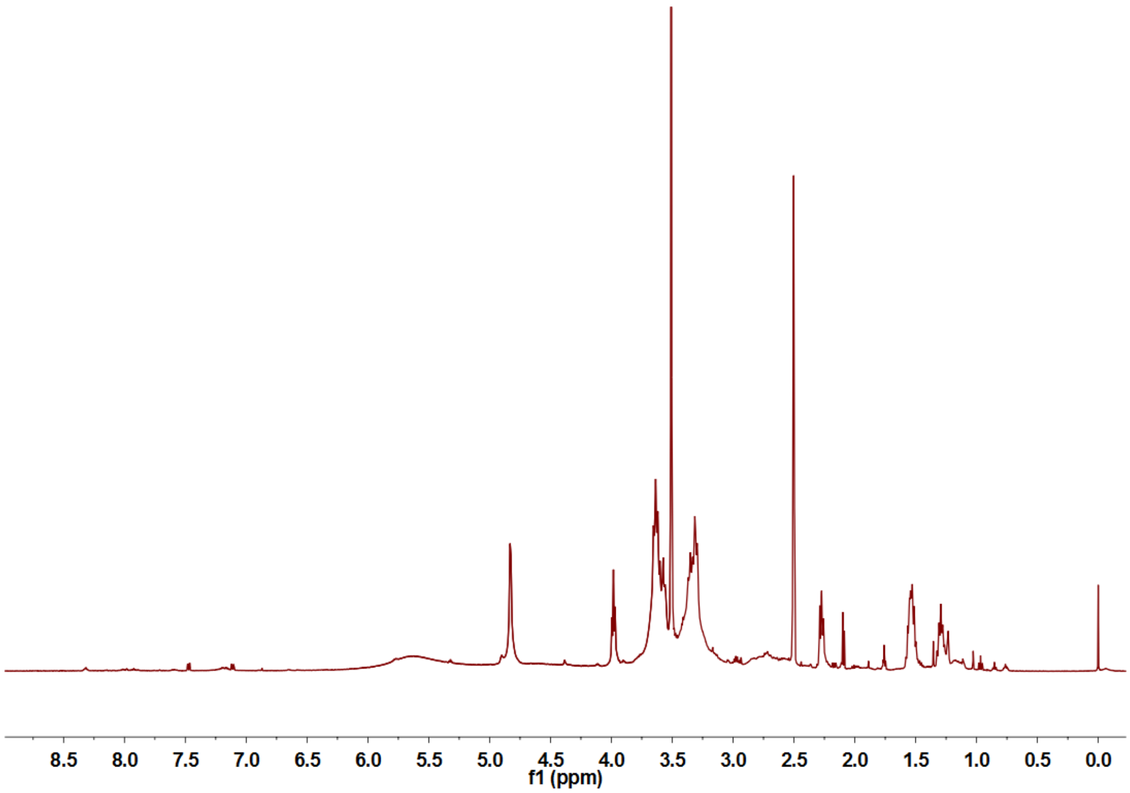

Supplementary Figure 10. ^{1}H NMR spectrum (300 MHz, DMSO-d_6, room temperature) of **PDI-PCL-*b*-PEG-RGD⊃*β*-CD-NH_2**.

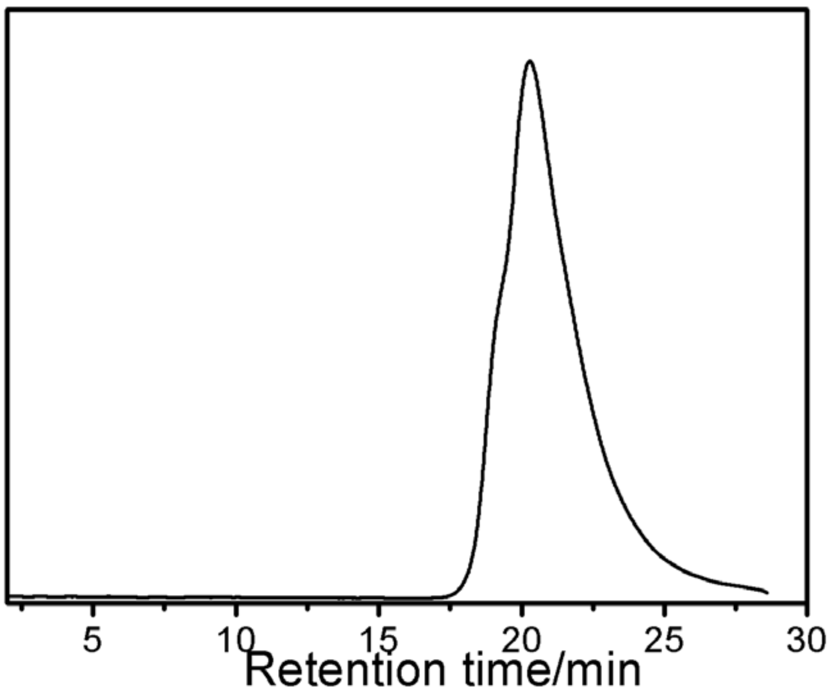

Supplementary Figure 11. GPC curve of **PDI-PCL-*b*-PEG-RGD⊃*β*-CD-NH_2**.

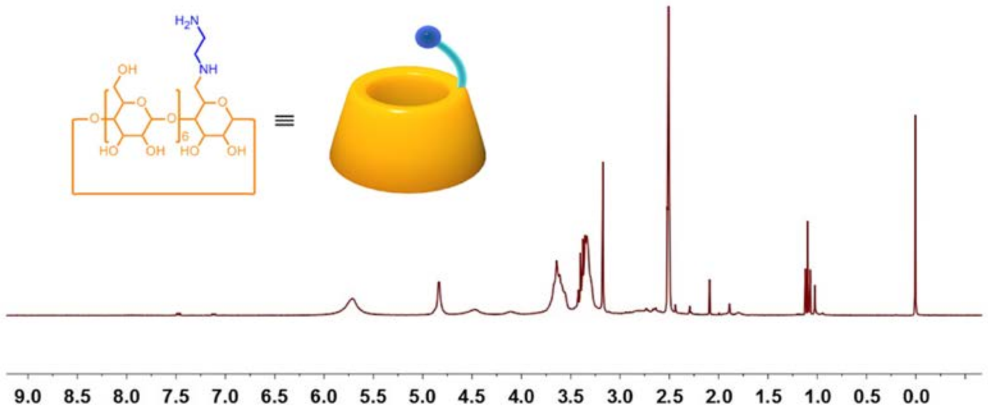

Supplementary Figure 12. ^1H NMR spectrum (300 MHz, DMSO-d_6, room temperature) of ***β*-CD-NH$_2$**.

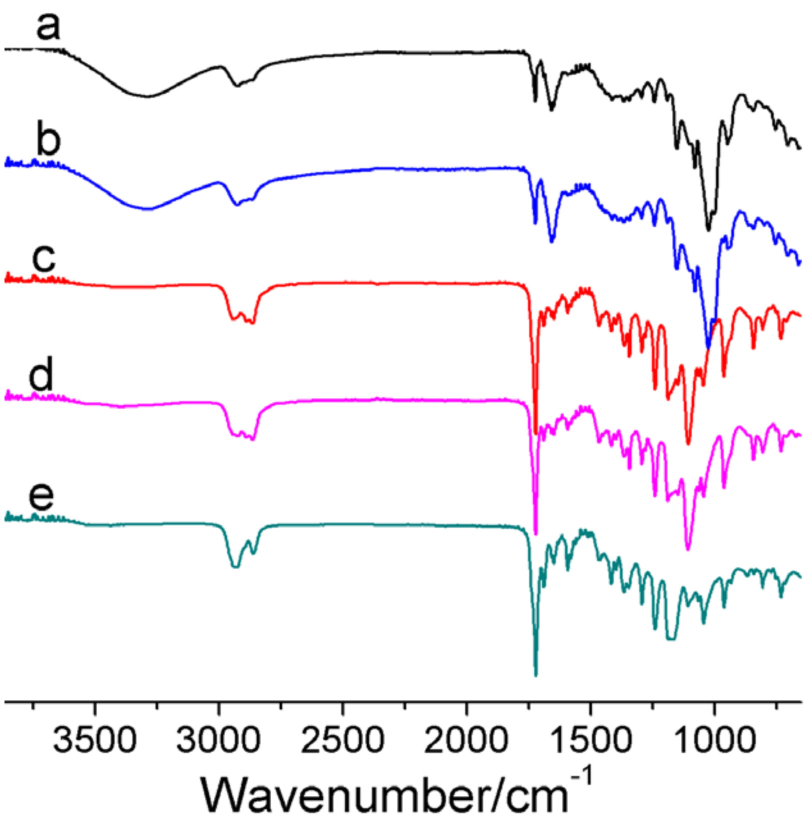

Supplementary Figure 13. FTIR spectra of (a) **SCNPs**, (b) **PDI-PCL-*b*-PEG-RGD⊃*β*-CD-NH$_2$**, (c) **PDI-PCL-*b*-PEG-Mal**, (d) **PDI-PCL-COOH**, and (e) **PDI-PCL**.

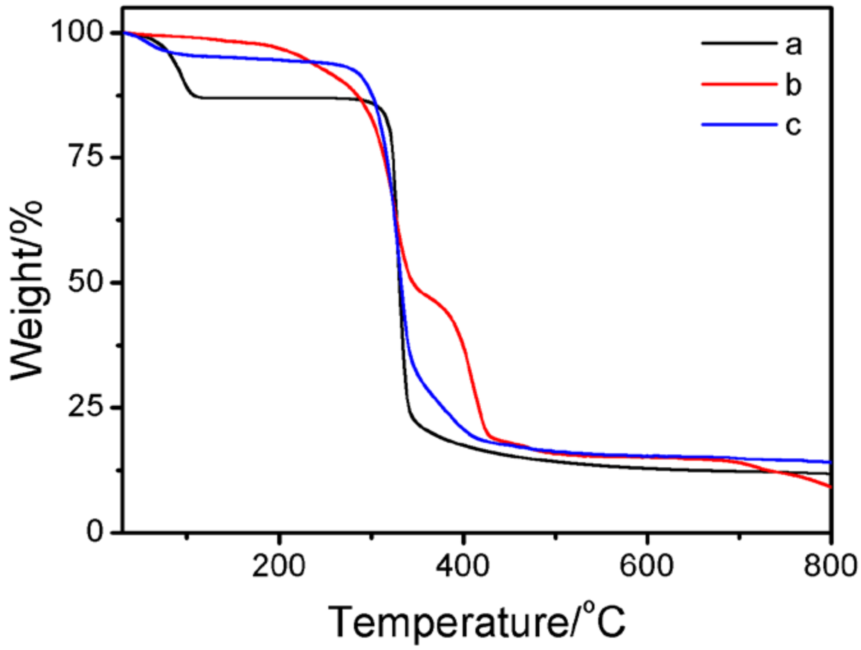

Supplementary Figure 14. TGA thermograms of (a) ***β*-CD-NH$_2$**, (b) **PDI-PCL-*b*-PEG-Mal**, and (c) **PDI-PCL-*b*-PEG-RGD⊃*β*-CD-NH$_2$** from 30 °C to 800 °C at a rate of 10 °C min^{-1} under nitrogen atmosphere.

Thermal properties of ***β*-CD-NH$_2$**, **PDI-PCL-*b*-PEG-Mal,** and **PDI-PCL-*b*-PEG-RGD⊃*β*-CD-NH$_2$** were also analyzed by thermogravimetric analysis (TGA) technique (Supplementary Fig. 14). Two decomposition temperatures at about 244 and 338 °C were observed for **PDI-PCL-*b*-PEG-Mal**, corresponding to the thermal degradation of **PCL-*b*-PEG** and **PDI** parts, respectively. For **PDI-PCL-*b*-PEG-RGD⊃*β*-CD-NH$_2$**, a three-step degradation pattern was detected. Dehydration of the polyrotaxane occurred when the temperature increased from about 50 °C to 125 °C, which indicated the presence of ***β*-CD-NH$_2$**. This was followed by the degradation of **PCL-*b*-PEG** and **PDI** by further increasing the experimental temperature.

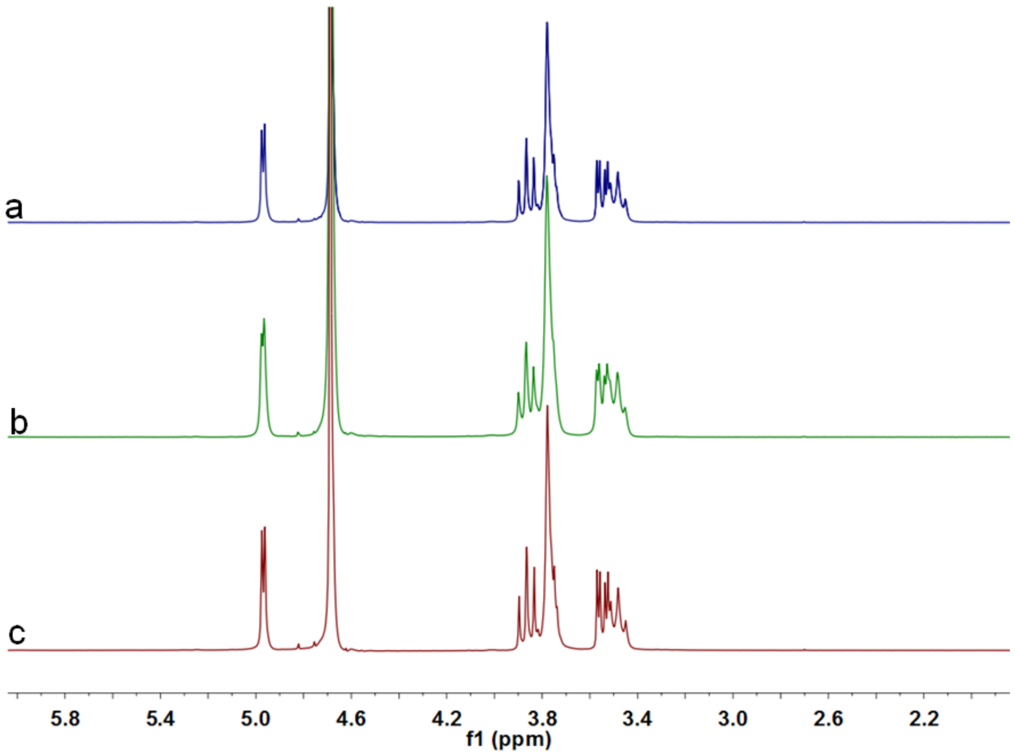

Supplementary Figure 15. ^{1}H NMR spectra (300 MHz, D_2O, room temperature) of (a) ***β*-CD**, (b) ***β*-CD** treated with **NHS-SS-NHS** in the presence of ethylenediamine, and (c) ***β*-CD** treated with **NHS-SS-NHS**.

In order to demonstrate that **NHS-SS-NHS** reacted with the amine group rather than the hydroxyl groups on ***β*-CD-NH$_2$**, we utilized ***β*-CD** as a model compound. After stirring the mixture of ***β*-CD** (2.00 mM) and **NHS-SS-NHS** (1.00 mM) for 24 h in the presence of ethylenediamine (2.00 mM), the solution was dialyzed against water for 2 day (MWCO 1 kDa) and then lyophilized for ^{1}H NMR study. As shown in Supplementary Fig. 15b, no signals were monitored corresponding to the methylene protons on **NHS-SS-NHS**, demonstrating that **NHS-SS-NHS** preferred to reacting with the amine group. Moreover, we tested the reaction possibility between **NHS-SS-NHS** and hydroxyl groups in the absence of amine group. After stirring the mixture of ***β*-CD** (2.00 mM) and **NHS-SS-NHS** (1.00 mM) for 24 h, the solution was dialyzed against water for 2 day (MWCO 1 kDa) and lyophilized for ^{1}H NMR study. Similarly, no new signals were monitored (Supplementary Fig. 15c), confirming that the reaction between between **NHS-SS-NHS** and hydroxyl groups was negligible. These results demonstrated that the hydroxyl groups on ***β*-CD-NH$_2$** did not interfere the crosslinking reaction during the preparation of **SCNPs**.

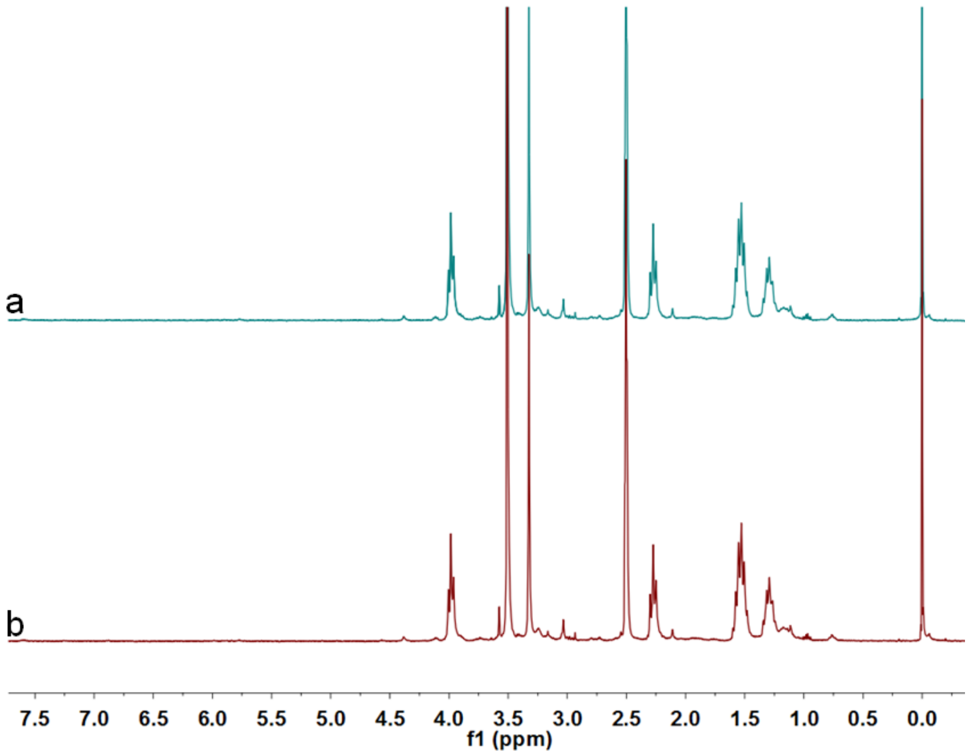

Supplementary Figure 16. ^{1}H NMR spectra (300 MHz, DMSO-d_6, room temperature) of (a) **PDI-PCL-*b*-PEG-Mal**, (b) **PDI-PCL-*b*-PEG-Mal** after culturing with ***β*-CD-NH$_2$**.

^{1}H NMR spectroscopy was utilized to verify the possible reaction between maleimide and amine group during the preparation of polyrotaxane. **PDI-PCL-*b*-PEG-Mal** (65.9 mg) and ***β*-CD-NH$_2$** (235 mg,) were dissolved in the mixture of THF and water (1/1, *v*/*v*). The organic solvent was evaporated by stirring the solution for 12 h at room temperature. The solution was dialyzed against the mixture of DMSO/water (1/1, *v*/*v*) for 1 day (MWCO 12 kDa), then dialyzed against water for 1 day (MWCO 12 kDa) to eliminate free ***β*-CD-NH$_2$**. The aqueous solution was lyophilized for ^{1}H NMR study. As shown in Supplementary Fig. 16b, negligible signals related to the protons of ***β*-CD-NH$_2$** were monitor, demonstrating that the amine group did not interfere with the reaction between maleimide and thiol group. Actually, the Michael addition reaction between maleimide and amine always takes place at reletively high temperature. Apart from the ^{1}H NMR studies, other experiments were also verify the successful preparation of polyrotaxane with the cRGDfK as one of the stopper, such as CLSM images (Fig. 5b, Supplementary Fig. 30, and Supplementary Fig. 66), cellular uptake studies (Fig. 5f and Supplementary Fig. 65), and MTT assay (Fig. 5g), in which targeting capacity of **SCNPs** was confirmed due to the existence of cRGDfK.

LC-MS was employed to measure the amount of NHS or COOH groups existed in **SCNPs** after crosslinking reaction. After culturing the solution of **SCNPs** with excess of tris(2-carboxyethyl)phosphine hydrochloride for 4 h, the small molecular weighted components cleaved from polyrotaxane were collected through ultrafiltration centrifugation (MWCO 10 kDa) for LC-MS study. However, no peaks were monitor corresponding to $SHCH_2CH_2COOH$ (m/z = 106) or $SHCH_2CH_2COONHS$ (sodium form

m/*z* = 305, acid form *m*/*z* = 283), demonstrating that negligible NHS or COOH groups existed in **SCNPs**. The reason was that the amount of NHS added was less than that of ***β*-CD-NH$_2$**, and the –NHS/NH$_2$– reaction was highly efficient.

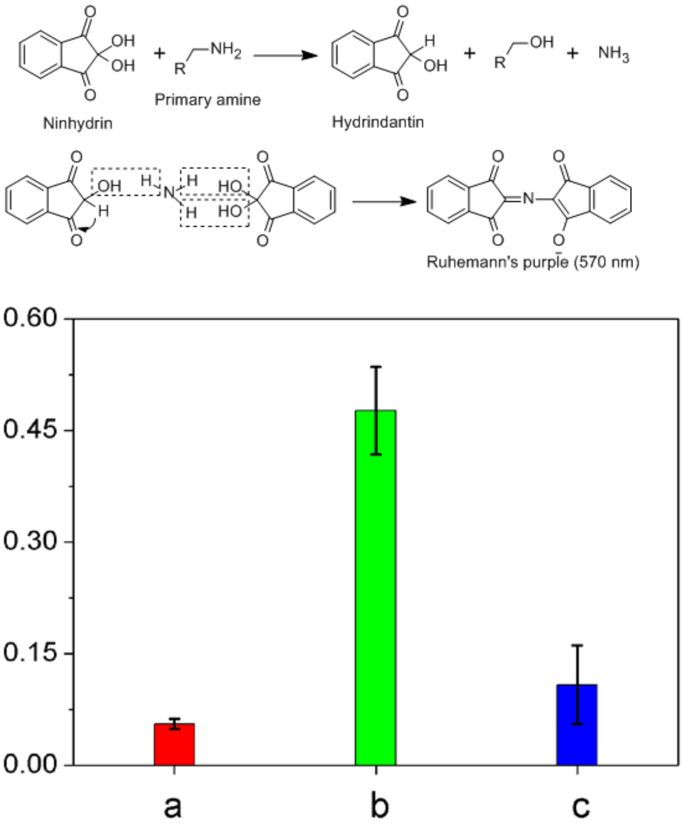

Supplementary Figure 17. Absorbance intensity at 570 nm of **PDI-PCL-*b*-PEG-RGD**, (b) **PDI-PCL-*b*-PEG-RGD⊃*β*-CD-NH$_2$**, and (c) **SCNPs** after reaction with ninhydrin.

Crosslinking efficient (CE) was calculated using the following equation:

CE = $(A_c - A_a)/(A_b - A_a) * 100\%$

Where A_a, A_b, and A_c represent the absorbance intensity at 570 nm of the aqueous solution containing **PDI-PCL-*b*-PEG-Mal**, **PDI-PCL-*b*-PEG-RGD⊃*β*-CD-NH$_2$**, and **SCNPs**, respectively. The molar concentration of these three samples kept same. For the **SCNPs** used in our further studies, the CE value was calculated to be 84.7%, suggesting that small amount of amine groups existed in **SCNPs** that could be used to conjugate **NHS-DOTA** and **NHS-Cy5.5** for PET imaging and fluorescence imaging.

Supplementary Table 1. Preparation of seven polyrotaxanes containing various *β*-CD-NH_2.

	PDI-PCL-*b*-PEG-Mal (μmol)	*β*-CD-NH_2 (μmol)	Average number of *β*-CD-NH_2[a]	PTX (mg)	DLC (%)[b]	NHS-SS-NHS (μmol)	Crosslinking efficient (%)[c]	Average size (nm)[d]	Stability
Polyrotaxane 1	10.0	20.0	0.3	50.0	22.7	1.50	Non-detectable	94.7	No precipitation over 2 d
Polyrotaxane 2	10.0	40.0	1.1	50.0	23.3	5.00	~37.2	105	No precipitation over 2 d
Polyrotaxane 3	10.0	80.0	2.6	50.0	27.4	10.0	~59.3	118	No precipitation over 2 d
Polyrotaxane 4	10.0	100	4.2	50.0	28.6	20.0	~77.6	124	No precipitation over 2 d
Polyrotaxane 5	10.0	150	5.7	50.0	32.1	25.0	~83.1	121	No precipitation over 2 d
Polyrotaxane 6	10.0	200	7.3	50.0	35.4	35.0	~84.7	128	No precipitation over 2 d
Polyrotaxane 7	10.0	300	8.2	50.0	29.2	40.0	~86.2	136	No precipitation over 2 d

[a] The number of ***β*-CD-NH_2** on the polyrotaxanes was calculated on the basis of the ^{1}H NMR spectra by comparing the integrations of the peaks related to the protons on ***β*-CD-NH_2** with the integration of the peaks related to the PEG segment.

[b] Drug loading content (DLC) = $m_{load}/(m_{load} + m) * 100\%$, where m represents the polymer mass used during the preparation of drug-loaded **SCNPs**.

[c] The crosslinking effient was calculated on the basis of ninhydrin reaction.

[d] The size of the PTX-loaded **SCNPs** was measured by using DLS.

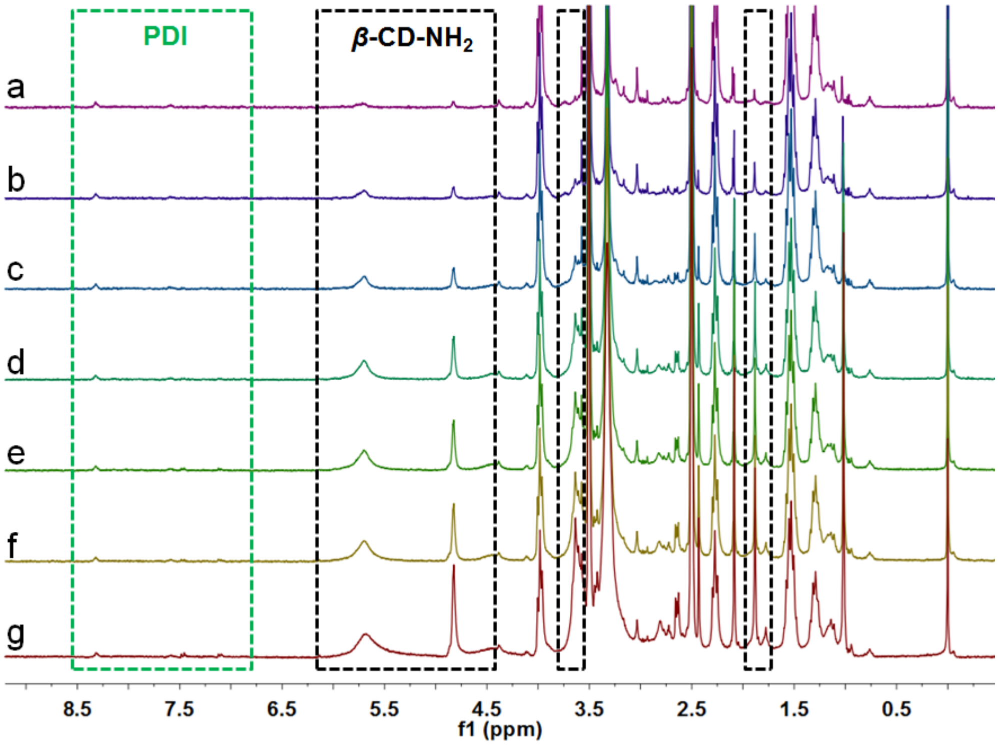

Supplementary Figure 18. ^{1}H NMR spectra (300 MHz, DMSO-d_6, room temperature) of (a) Polyrotaxane 1, (b) Polyrotaxane 2, (c) Polyrotaxane 3, (d) Polyrotaxane 4, (e) Polyrotaxane 5, (f) Polyrotaxane 6, and (g) Polyrotaxane 7.

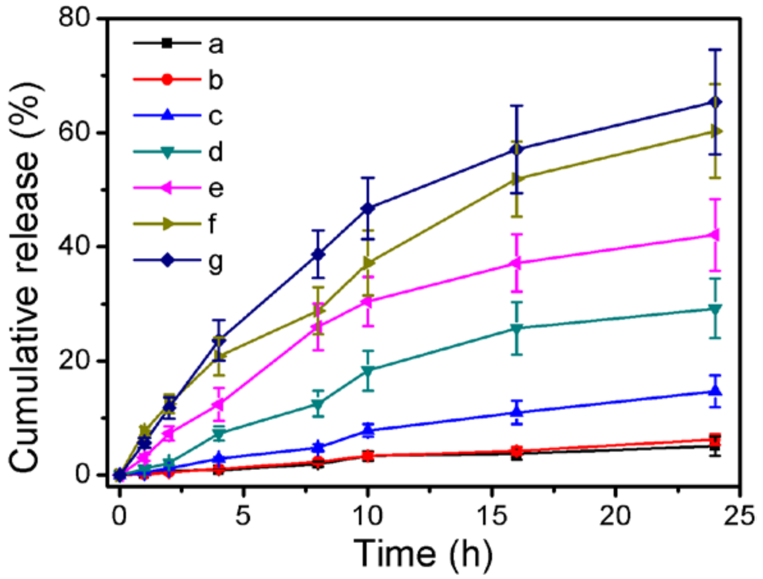

Supplementary Figure 19. Controlled release profiles of (a) **SCNPs**@PTX prepared from polyrotaxane 7, (b) **SCNPs**@PTX prepared from polyrotaxane 6, (c) **SCNPs**@PTX prepared from polyrotaxane 5, (d) **SCNPs**@PTX prepared from polyrotaxane 4, (e) **SCNPs**@PTX prepared from polyrotaxane 3, (f) **SCNPs**@PTX prepared from polyrotaxane 2, and (g) **SCNPs**@PTX prepared from polyrotaxane 1. Data are expressed as mean ± s.e.m. of three independent experiments.

As shown in Supplementary Fig. 19, the release rate of the PTX-loaded **SCNPs** decreased significantly by increasing the number of ***β*-CD-NH$_2$** in the polyrotaxanes and their crosslinking efficient. The reason was that the wheels (***β*-CD-NH$_2$**) of the polyrotaxanes acted as "molecular gates" to inhibit the release of the loaded PTX by forming shell crosslinked nanoparticles. This property is extremely important to avoid premature drug release during their blood circulation, which is able to effectively reduce side effect towards normal tissues.

Self-assembly studies

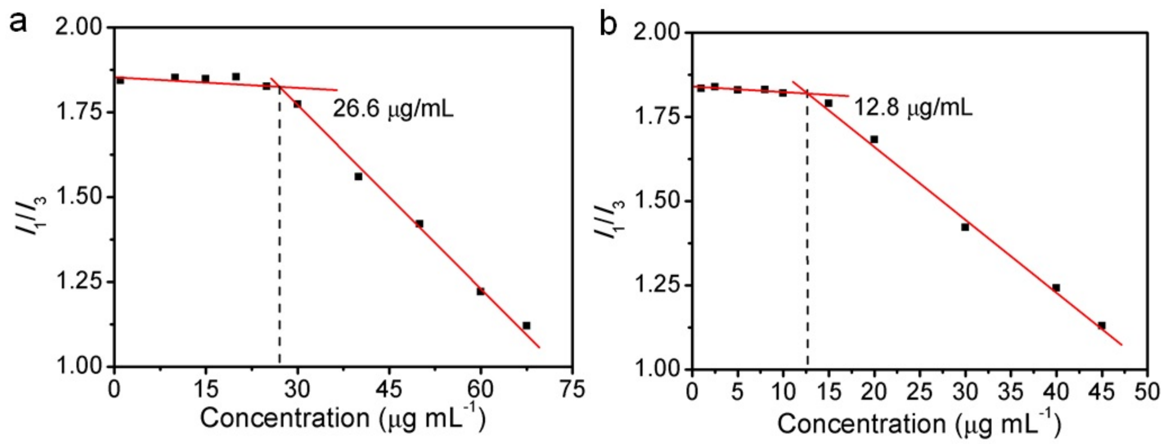

Supplementary Figure 20. Determination of CAC values for **PDI-PCL-*b*-PEG-Mal** and **PDI-PCL-*b*-PEG-RGD⊃*β*-CD-NH$_2$** by using the fluorescent method with pyrene as a probe.

The critical aggregation concentration of **PDI-PCL-*b*-PEG-RGD⊃*β*-CD-NH$_2$** was measured to be 26.6 μg mL^{-1}, about 2-fold that of **PDI-PCL-*b*-PEG-RGD** (12.8 μg mL^{-1}), suggesting that the introduction of water-soluble cyclodextrin inceased the solubility of the diblock polymeric axle (Supplementary Fig. 20), confirming the fabrication of **PDI-PCL-*b*-PEG-RGD⊃*β*-CD-NH$_2$** through cyclodextrin-based host−guest chemistry.

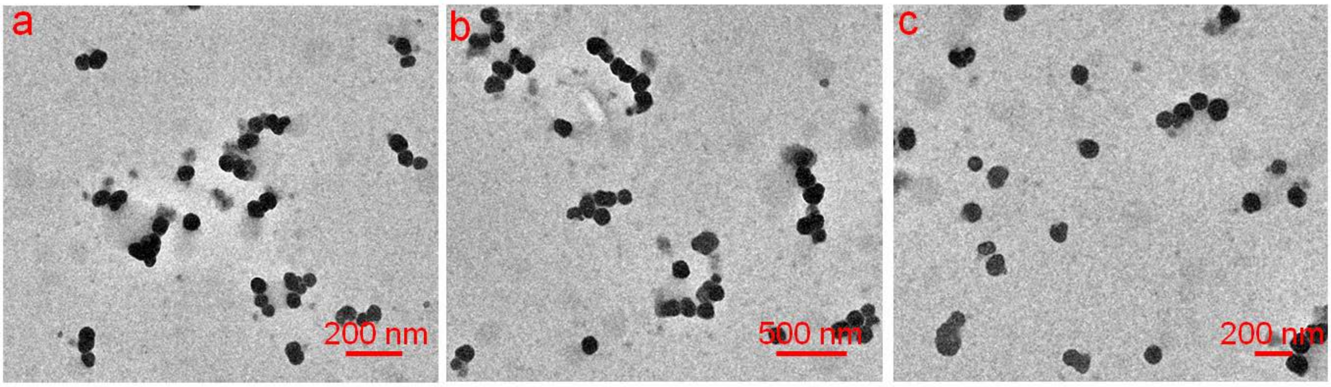

Supplementary Figure 21. TEM images of (a) NPs formed from **PDI-PCL-*b*-PEG-RGD⊃*β*-CD-NH$_2$**, (b) **SCNPs**@PTX, and (c) **SCNPs**@CPT.

In vitro photothermal and photoacoustic studies

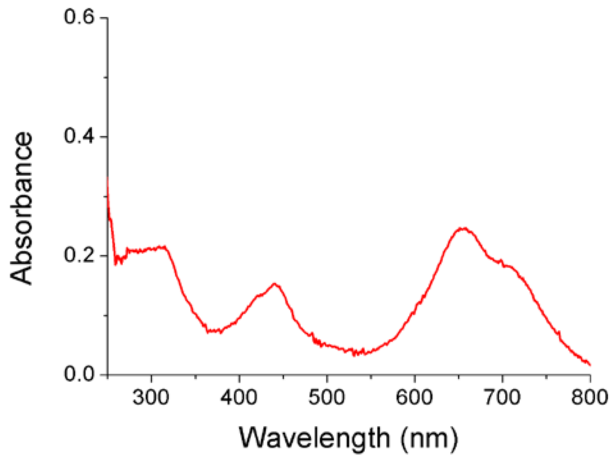

Supplementary Figure 22. UV-vis spectrum of **SCNPs** at a concentration of 0125mg mL^{-1}.

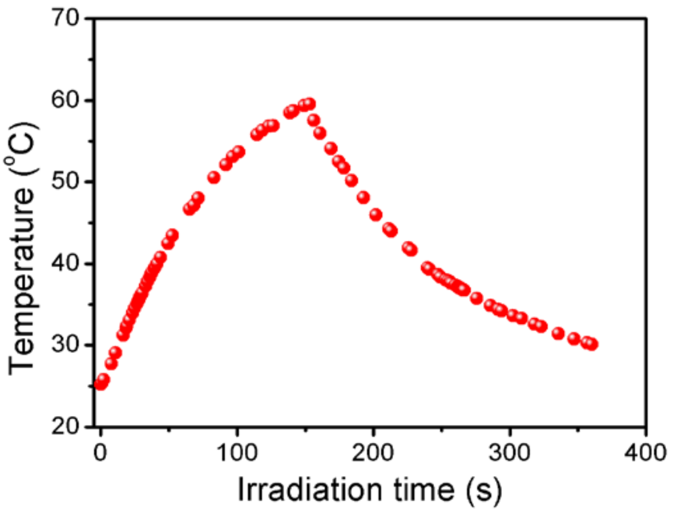

Supplementary Figure 23. Photothermal effect of **SCNPs** aqueous solution (0.250 mg mL^{-1}) when radiated by a 671 nm laser (0.5 W cm^{-2}). The laser was removed after irradiating sample for 150 s.

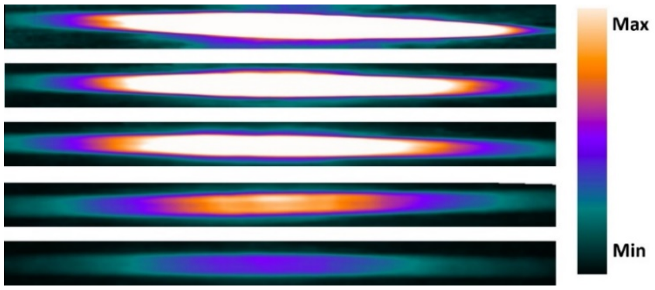

Supplementary Figure 24. PA images of **SCNPs** at different concentration in tube.

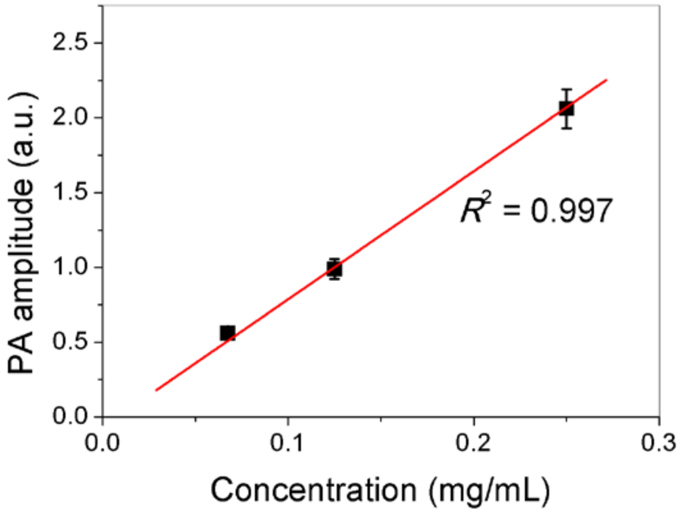

Supplementary Figure 25. PA intensity of the aqueous solution containing **SCNPs** as a function of concentration. Data are expressed as mean ± s.e.m. of three independent experiments.

The PA amplitudes and images at 671 nm were measure with the concentration of **SCNPs** ranging from 67.5 to 250 µg mL^{-1} (Supplementary Fig. 24–25), exhibiting an excellent linear relationship between the concentrations and PA signals.

In vitro thermo-chemotherapy investigations of ***SCNPs****@PTX*

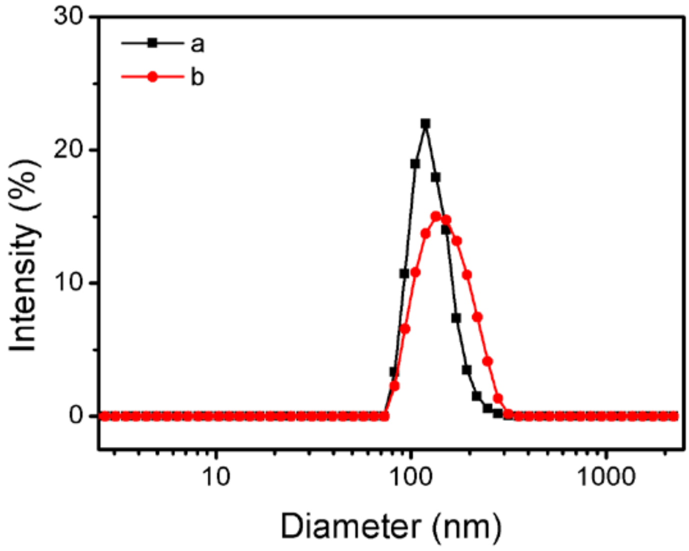

Supplementary Figure 26. DLS results of (a) **SCNPs**@PTX and (b) **SCNPs**@CPT.

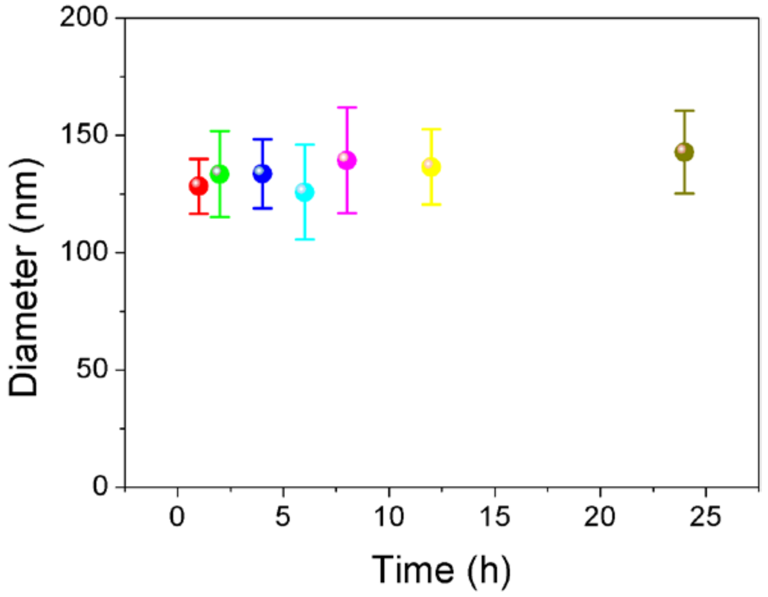

Supplementary Figure 27. The diameter changes of **SCNPs**@PTX determined by DLS tests in PBS containing 10% FBS after different time periods of incubation. Data are expressed as mean ± s.e.m. of three independent experiments.

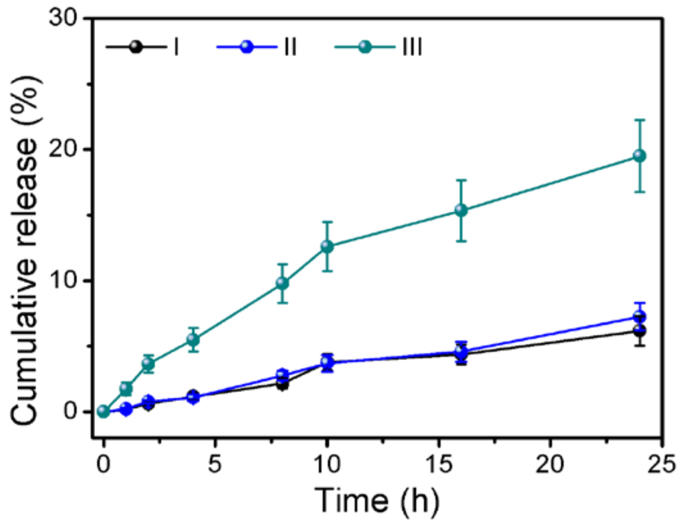

Supplementary Figure 28. Controlled release profiles of (I) **SCNPsCC**@PTX without any treatments, (II) **SCNPsCC**@PTX in the presence of 10.0 mM GSH, and (III) **SCNPsCC**@PTX in the presence of 10.0 mM GSH upon laser irradiation (671 nm, 0.5 W cm^{-2}). Data are expressed as mean ± s.e.m. of three independent experiments.

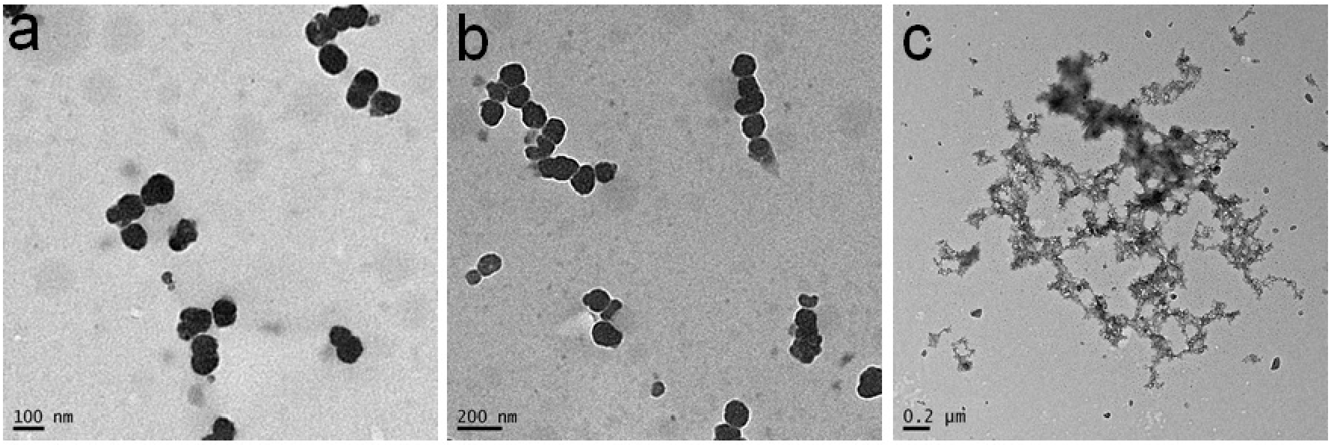

Supplementary Figure 29. TEM image of **SCNPs**@PTX after culturing with (a) GSH (1.00 mM) and (b) GSH (10.0 mM) for 24 h. (b) TEM image of **SCNPs**@PTX after culturing with GSH (10.0 mM) for 24 h upon laser irradiation (671 nm, 0.5 W cm^{-2}, 5 min).

We used TEM to study the morphology changes of the **SCNPs** in the presence of GSH with various concentrations. As shown in Supplementary Fig. 29, rare changes in size and morphology of **SCNPs** were observed after culturing them with GSH for 24 h, because the structure of **SCNPs** could be maintained by the π-π stacking interaction between PDI groups and hydrophobic interactions between the PCL segments. Upon laser irradiation (671 nm, 0.5 W cm^{-2}) for 5 min, **SCNPs** disassembled into irregular aggregated with larger diameter because of the photothermal effect (Supplementary Fig. 29c).

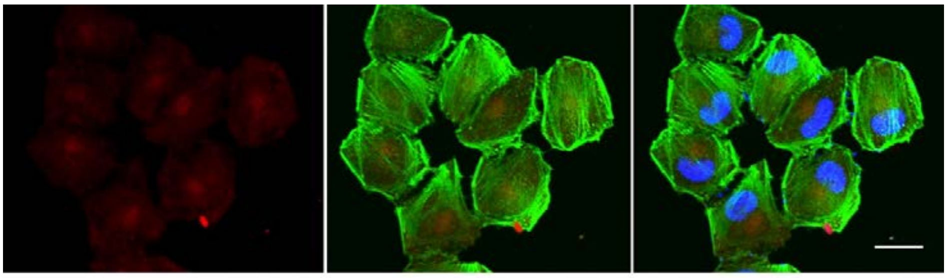

Supplementary Figure 30. CLSM images of the HeLa cells cultured with Cy5.5-labeled **SCNPs**@PTX pre-treated with cRGDfK (20 μM) for 30 min. Blue fluorescence shows nuclear staining with Hoechst 33342; red fluorescence shows the location of **SCNPs**@PTX staining with Cy5.5; green fluorescence shows *β*-actins staining with FITC-phalloidin. Scale bar is 20 μm.

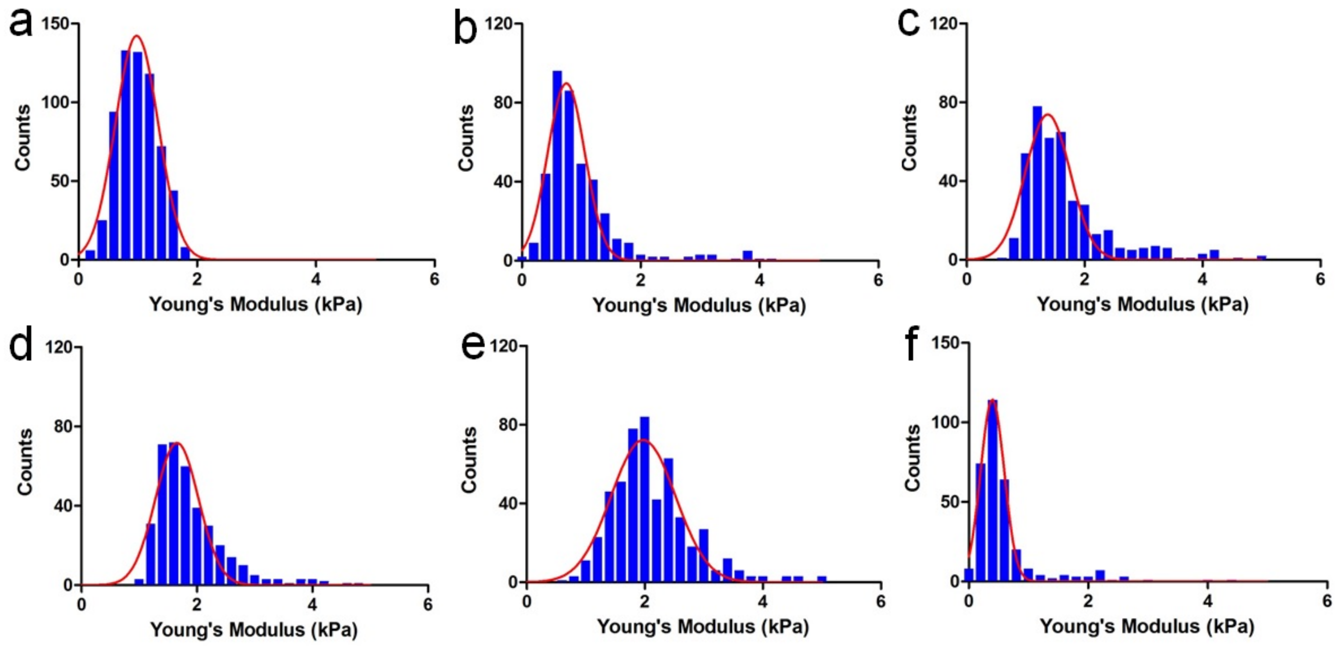

Supplementary Figure 31. Histogram of reduced Young's modulus for all data collected from six different groups: (a) control, (b) **SCNPsCC**@PTX (PTX, 50.0 nM), (c) PTX (10.0 nM), (d) **SCNPs**@PTX (PTX, 10.0 nM), (e) **SCNPs**@PTX (PTX, 50.0 nM), (f) **SCNPs** + laser (671 nm, 0.1 W cm^{-2}, 3 min). Red lines are the Gaussian fit for the data.

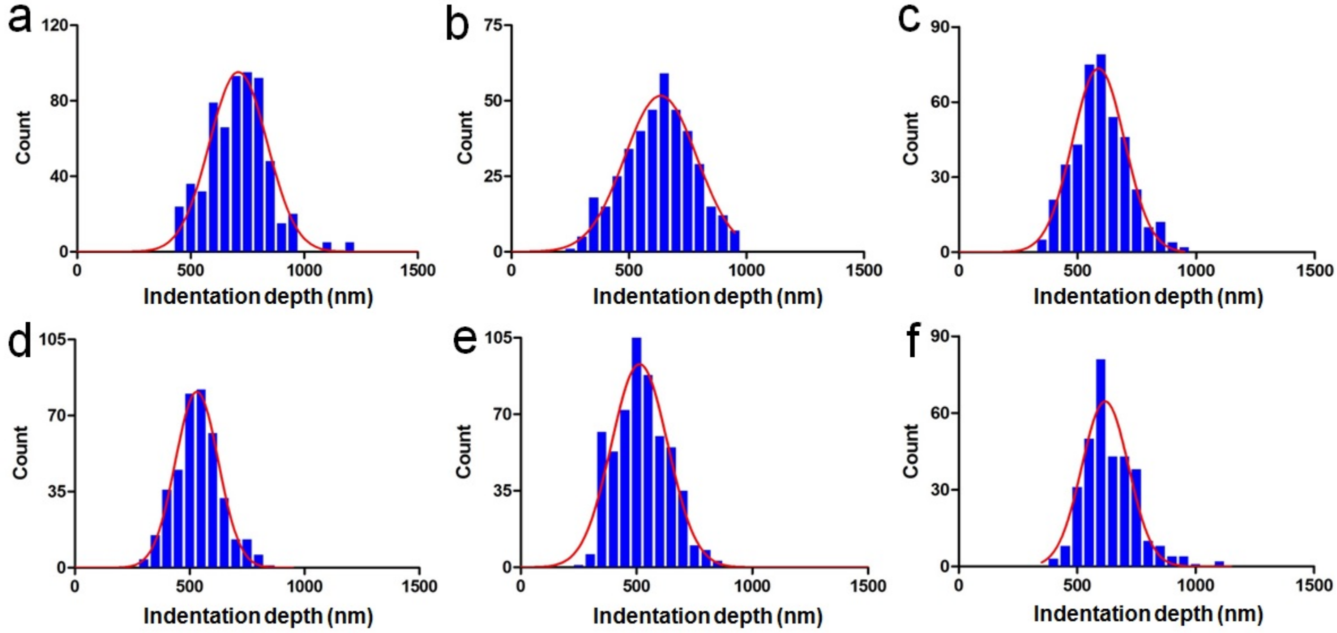

Supplementary Figure 32. Histogram of indentation depth for all data collected from six different groups: (a) control, (b) **SCNPsCC**@PTX (PTX, 50.0 nM), (c) PTX (10.0 nM), (d) **SCNPs**@PTX (PTX, 10.0 nM), (e) **SCNPs**@PTX (PTX, 50.0 nM), (f) **SCNPs** + laser (671 nm, 0.1 W cm^{-2}, 3 min). Red lines are the Gaussian fit for the data.

PTX is a cytoskeletal drug that targets tubulin by binding to the *N*-terminal 31 amino acids of the tubulin subunit, thereby stabilizing the microtubule polymers and inhibiting disassembly. As a consequence, the mechanical properties of the cells change remarkably due to the formation of redundant microtubules. In the hope of providing accurate, quantitative, and orthogonal insights into assessments of the therapeutic performances in these groups, a statistical analysis of alterations in cell elasticity (reduced Young's modulus, *E*) and indentation depth acquisition were conducted on multiple cells (Fig. 5a), and their respective histograms are shown in Fig. 5 and Supplementary Fig. 31–32. For the cells without any treatment, the reduced Young's modulus displayed a normal Gaussian distribution, and the *E* value was calculated to be 0.98 ± 0.08 kPa. After different treatments (**SCNPsCC**@PTX, free PTX, **SCNPs**@PTX, and **SCNPs** + laser), the distribution of reduced Young's modulus became much broader due to the action of PTX and photothermal effect. By analysizing these data, we discovered that the cell mechanical stiffness decreased significantly due to the photothermal effect. The *E* value dropped to 0.57 ± 0.04 kPa after receiving photothermal treatment (Fig. 5d), possibly because the formed microtubules disassembled at relatively high temperature. Compared with the free PTX (2.31 ± 0.10 kPa), only a slight improvement in *E* value was detected for the cells treated with **SCNPsCC**@PTX (1.16 ± 0.08 kPa), which indicated that the release of the sealed drug was inhibited, because the "supramolecular gates" were barely opened inside the cells using **NHS-CC-NHS** as crosslinkers. Notably, the *E* value increased to 1.93 ± 0.09 kPa for the cells after treatment with **SCNPs**@PTX (PTX, 10 nM), which was comparable to that of free PTX, because the encapsulated PTX was opportunely released from **SCNPs**@PTX triggered by intracellular GSH. When the concentration of **SCNPs**@PTX increased to 50 nM PTX, the *E* value further increased to 2.80 ± 0.11 kPa. In terms of indentation depth, opposite trends were observed; greater cell stiffness resulted in low indentation depth (Fig. 5e and Supplementary Fig. 32). These two complementary parameters demonstrated that **SCNPs** are promising DDSs to deliver hydrophobic PTX.

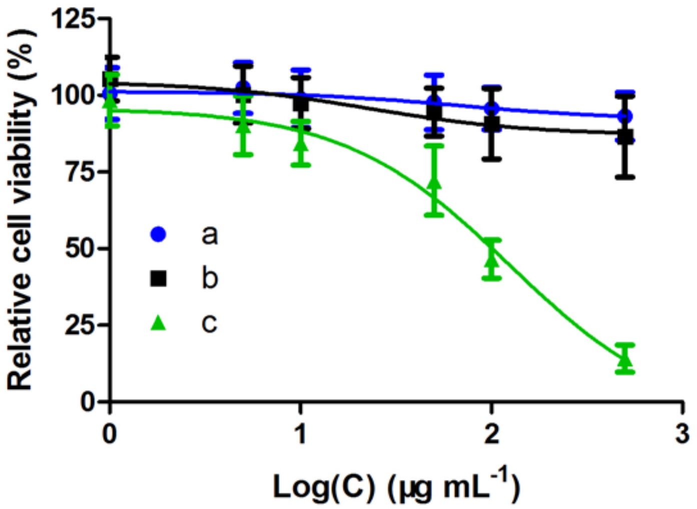

Supplementary Figure 33. Relative cell viability of HeLa cells incubated with various concentrations of (a)

SCNPs, (b) **SCNPs** + laser (671 nm, 0.1 W cm^{-2}, 3 min), and (c) **SCNPs** + laser (671 nm, 0.5 W cm^{-2}, 3 min). Data are expressed as mean ± s.e.m. of five independent experiments.

Cytotoxicity of the vehicles is an essential concern when it comes to the development of DDSs for biomedical applications. PCL, PEG, and *β*-CD are FDA-approved components with wide availability and excellent biocompatibility, making **SCNPs** a promising candidate for practical applications. As determined by 3-(4′,5′-dimethylthiazol-2′-yl)-2,5-diphenyl tetrazolium bromide (MTT) assay, the relative cell viability was higher than 90% for the HeLa cells cultured with **SCNPs,** even when the concentration of **SCNPs** reached 500 μg mL^{-1} (Supplementary Fig. 33), suggesting very low cytotoxicity. At low laser power density (0.1 W cm^{-2}), the culture temperature was around 41 °C as monitored by thermal camera, which was tolerable for the cells. As a result, the cell viability was as high as 86.4% when the concentration of **SCNPs** was 500 μg mL^{-1}. At high power density (0.5 W cm^{-2}), the relative cell viability decreased significantly by increasing the concentration of **SCNPs** (Supplementary Fig. 33) as a result of the relatively high temperature (~60 ^{o}C).

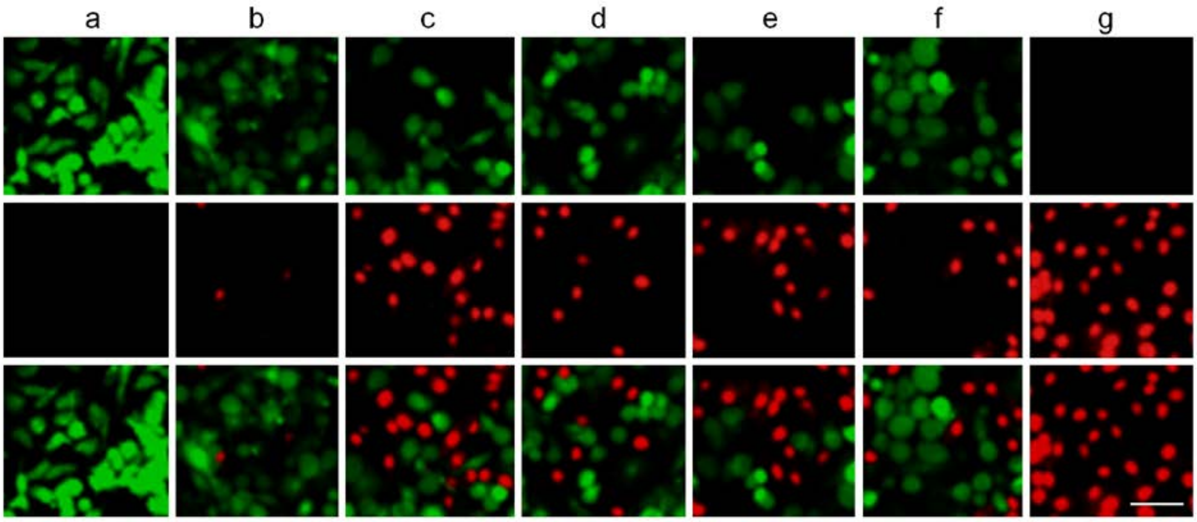

Supplementary Figure 34. Fluorescence images of calcein AM (green fluorescence; live cells) and propidium iodide (red fluorescence; dead cells) co-stained HeLa cells treated with (a) PBS, (b) **SCNPs** + laser (671 nm, 0.1 W cm^{-2}, 3 min), (c) PTX (100 nM), (d) **SCNPs**@PTX (PTX, 100 nM), (e) **SCNPs**@PTX (PTX, 100 nM) + laser (671 nm, 0.1 W cm^{-2}, 3 min), (f) **SCNPs** + laser (671 nm, 0.5 W cm^{-2}, 3 min), (g) **SCNPs**@PTX (PTX, 100 nM) + laser (671 nm, 0.5 W cm^{-2}, 3 min). Scale bar is 50 μm.

In vivo thermo-chemotherapy investigations of ***SCNPs**@PTX*

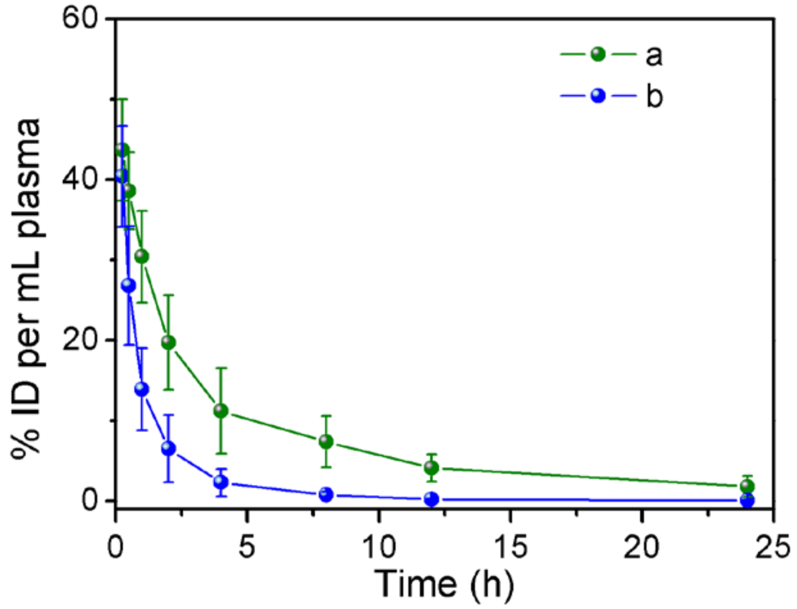

Supplementary Figure 35. *In vivo* blood elimination kinetics of (a) **SCNPs**@PTX and (b) PTX (n = 3 for each group).

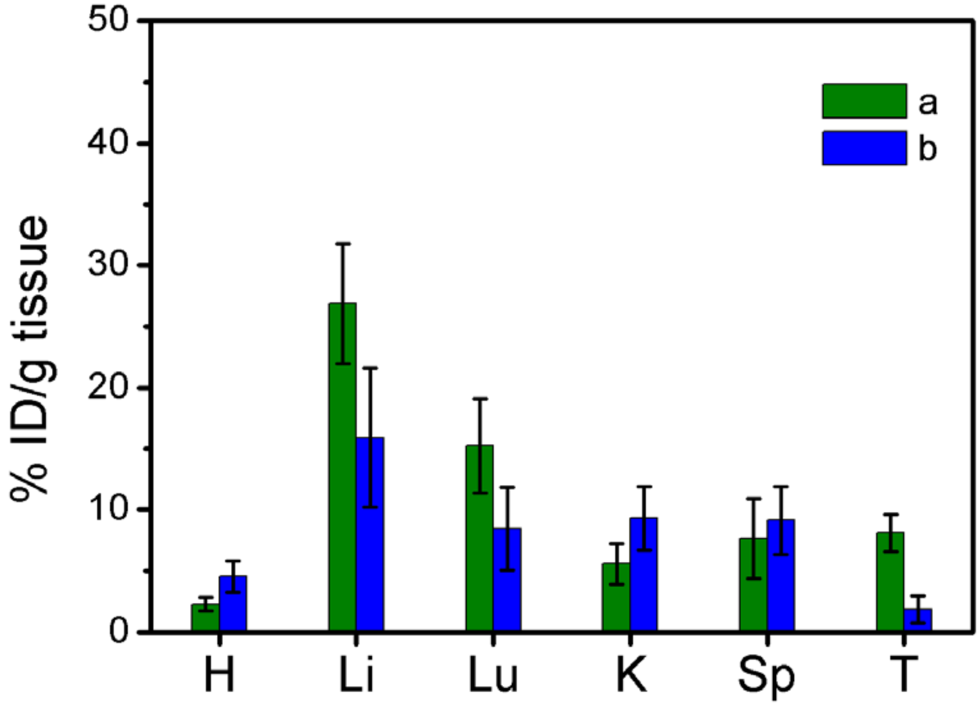

Supplementary Figure 36. Tissue distributions in the main organs after *i.v.* injection of (a) **SCNPs**@PTX and (b) PTX at 24 h post injection. (H: heart, Li: liver, Lu: lung, Sp: spleen, K: kidney, T: tumour). Data are expressed as mean ± s.e.m. of three independent experiments.

The bio-distribution of PTX in the main organs was measured by using HPLC, which was in good agreement with the result obtained from PET investigation (Fig. 6f).

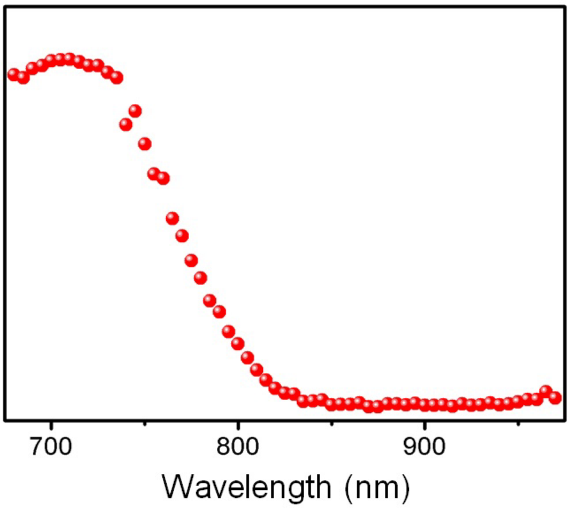

Supplementary Figure 37. PA spectrum of **SCNPs** in tumour tissue at 24 post injection.

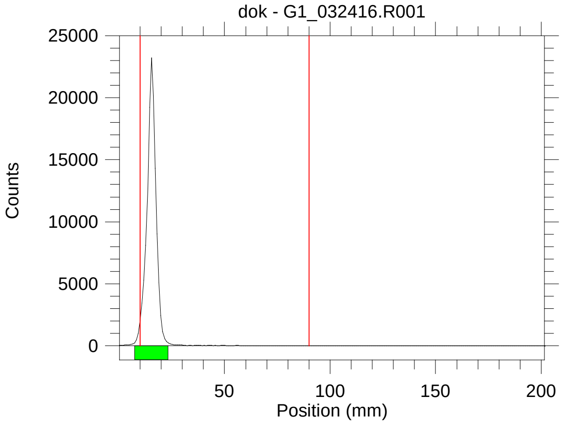

Supplementary Figure 38. Radio TLC chromatograms of ^{64}Cu **SCNPs**@DOTA after incubation in mouse serum for 24 h.

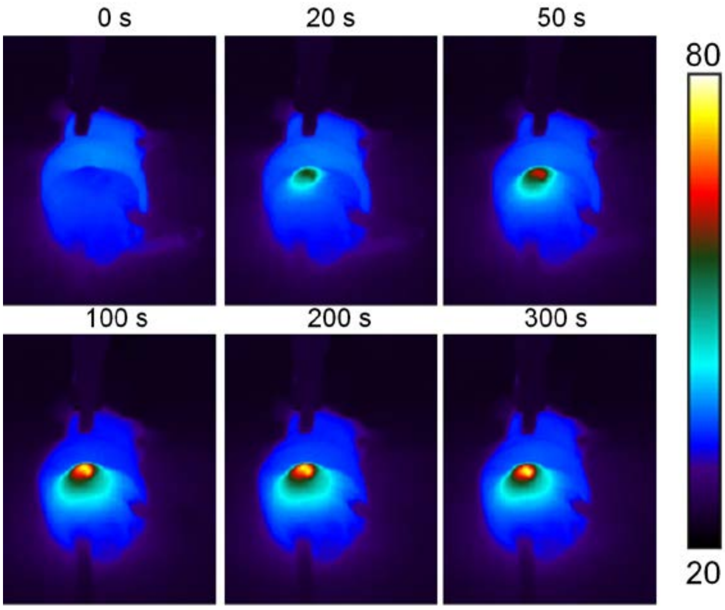

Supplementary Figure 39. IR thermal images of HeLa tumour-bearing mice administered by *i.v.* injection under the 671 nm laser exposure at 0.5 W cm^{-2} for 5 min.

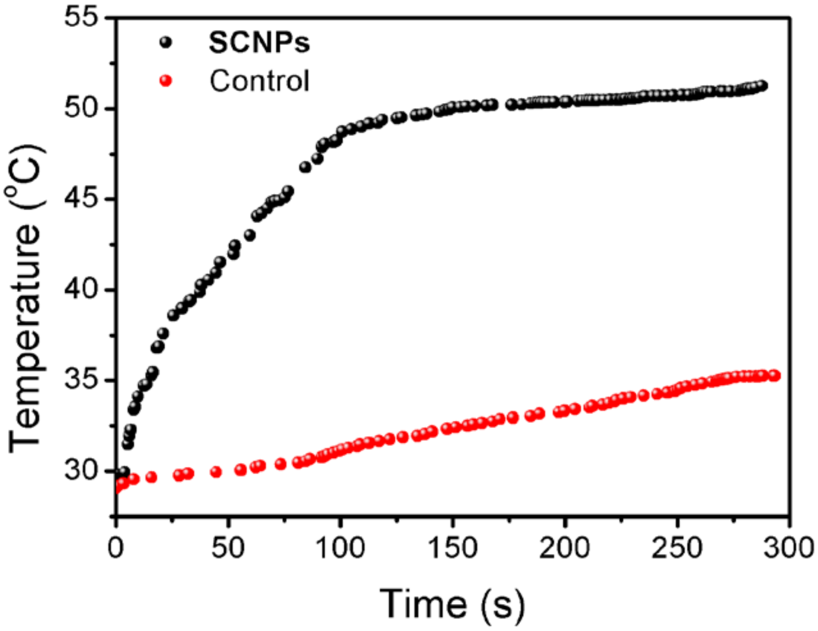

Supplementary Figure 40. The temperature raise on tumours of mice with/without **SCNPs** administered by *i.v.* injection under the 671 nm laser exposure at 0.5 W cm^{-2} for 5 min.

To evaluate the photothermal effects of **SCNPs**@PTX *in vivo*, we irradiated mice bearing HeLa tumours with NIR laser at 671 nm (0.5 W cm^{-2}) for a total of 5 min at 24 h post-injection (Supplementary Fig. 39). The local temperature of tumours was monitored by an infrared thermal camera. In the **SCNPs**@PTX-treated group, the local temperature of tumours increased rapidly from 29.8 to 51.3 °C, which was significant enough for heat-induced tumour inhibition (Supplementary Fig. 40). In comparison, the temperature at the irradiated area only reached 35.4 ^{o}C for the mice treated with PBS under the same conditions.

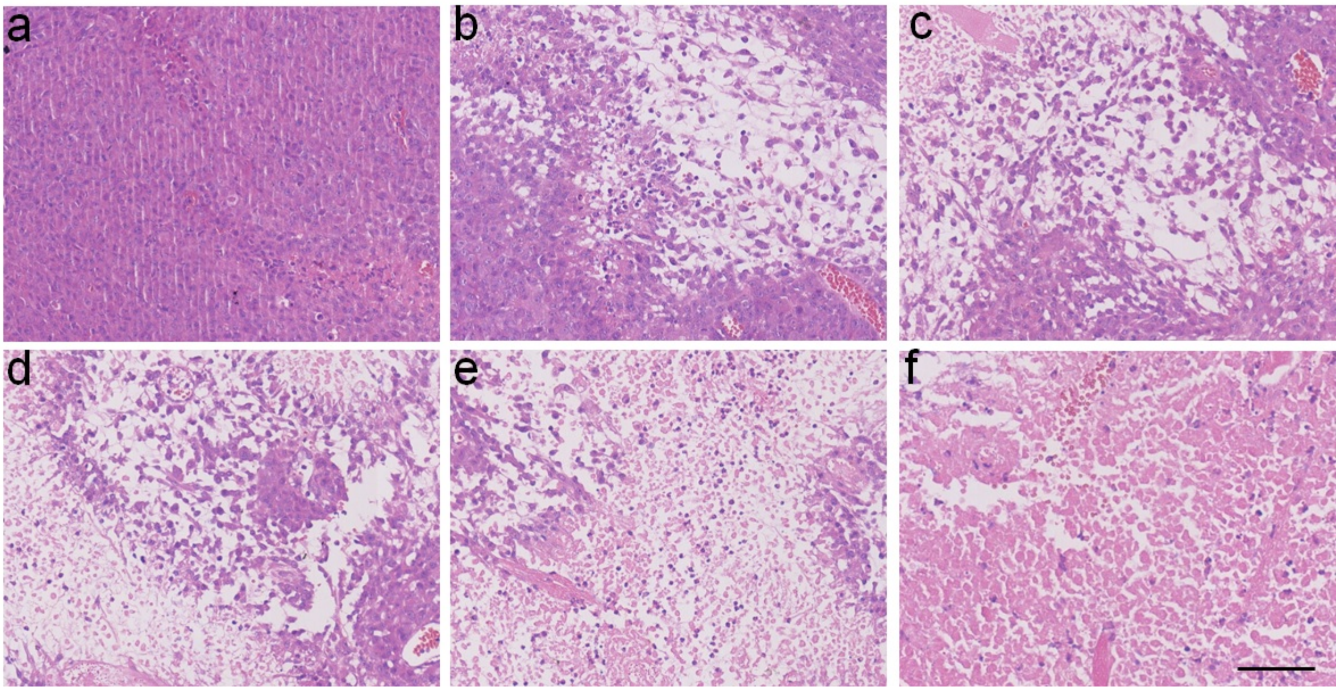

Supplementary Figure 41. H&E analyses of tumour tissues after treatment with various formulations: (a) PBS, (b) **SCNPs**@PTX (PTX, 20 mg kg^{-1}), (c) Abraxane (PTX, 20 mg kg^{-1}), (d) **SCNPs**@PTX (PTX, 60 mg kg^{-1}), (e) **SCNPs** + laser (671 nm, 0.5 W cm^{-2}, 5 min), (f) **SCNPs**@PTX (PTX, 60 mg kg^{-1}) + laser (671 nm, 0.5 W cm^{-2}, 5 min). Scale bar is 50 μm.

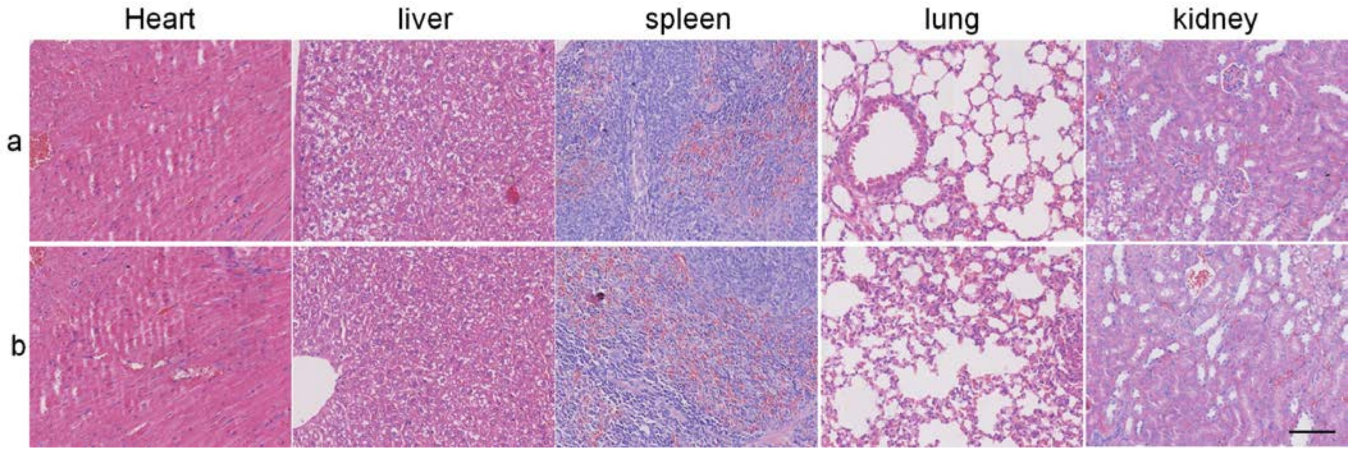

Supplementary Figure 42. H&E stained images of heart, liver, spleen, lung and kidney from different groups after treatment with various formulations: (a) PBS and (b) **SCNPs**@PTX (PTX, 60 mg kg^{-1}) + laser (671 nm, 0.5 W cm^{-2}, 5 min). Scale bar is 100 μm.

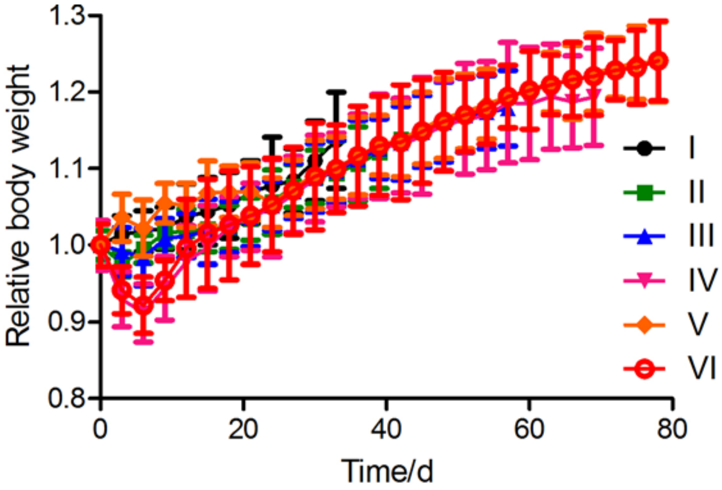

Supplementary Figure 43. Body weight changes of the mice (*n* = 10) treated with different formulations. I, PBS; II, **SCNPs**@PTX (PTX, 20 mg kg^{-1}); III, Abraxane (PTX, 20 mg kg^{-1}); IV, **SCNPs**@PTX (PTX, 60 mg kg^{-1}); V, **SCNPs** + laser (671 nm, 0.5 W cm^{-2}, 5 min); VI, **SCNPs**@PTX (PTX, 60 mg kg^{-1}) + laser (671 nm, 0.5 W cm^{-2}, 5 min). Data are expressed as mean ± s.e.m..

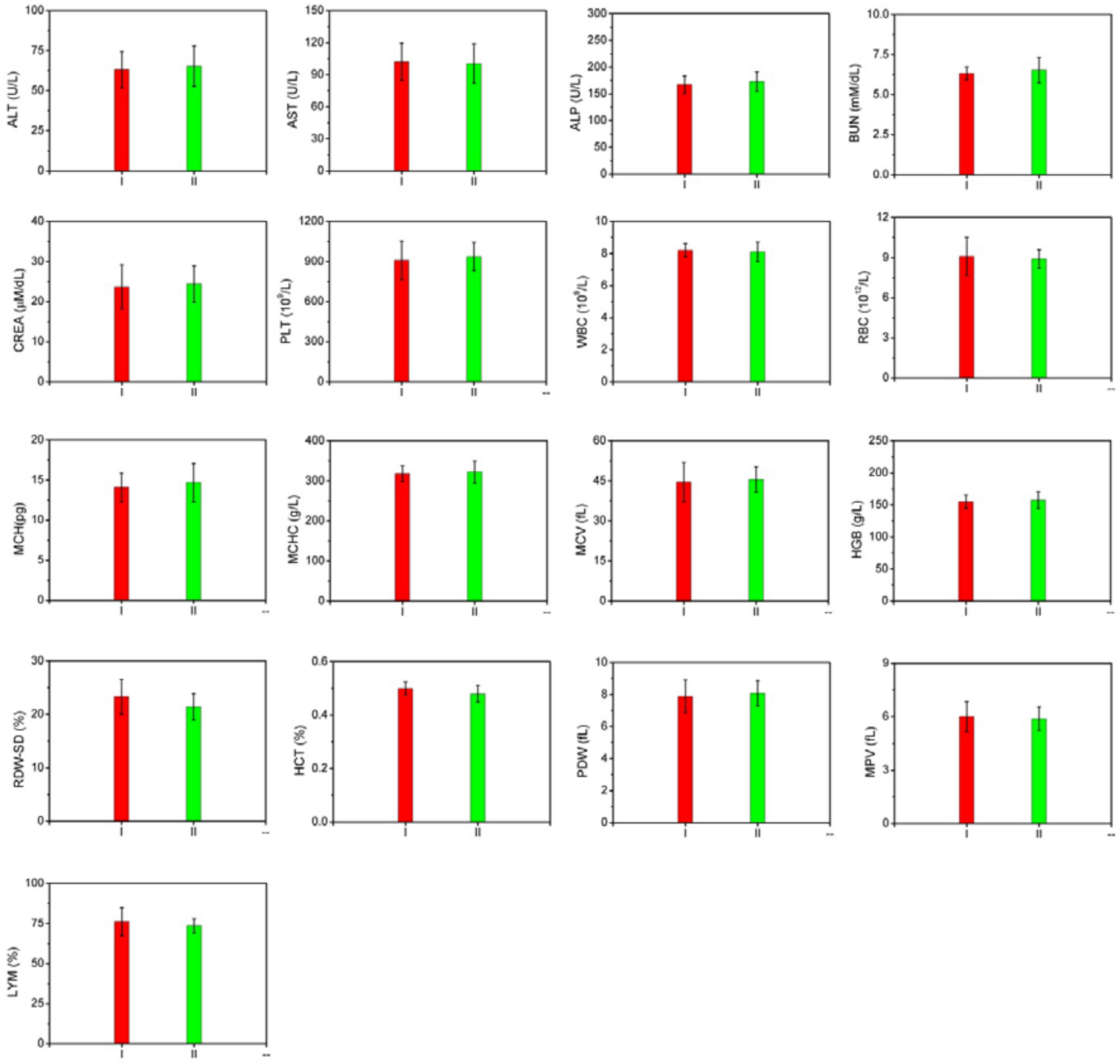

Supplementary Figure 44. The variations of blood index of the mice after treatment with various formulations: (I) PBS and (II) **SCNPs**@PTX (PTX, 60 mg kg^{-1}) + laser (671 nm, 0.5 W cm^{-2}, 5 min) at 15 d post-injection. Data are expressed as mean ± s.e.m. of three independent experiments.

Moreover, no obvious signs of toxic effects, such as changes in drinking, eating, grooming, activity, urination, or neurological status took place during the treatment period. Alanine aminotransferase (ALT), alkaline phosphatase (ALP), and aspartate aminotransferase (AST) as liver function indicators showed that no hepatic dysfunction was induced by the **SCNPs**@PTX (PTX, 60 mg kg^{-1}) + laser administration (Supplementary Fig. 44). On the other hand, no significant differences in renal function indexes including blood urea nitrogen (BUN) and creatinine (CREA) were detected between the mice treated with PBS and **SCNPs**@PTX (PTX, 60 mg kg^{-1}) + laser. Furthermore, negligible chnages were observed between the thermo-chemotherapy group and control group in the hematological tests, such as counts for platelets (PLT), white blood cells (WBC), red blood cells (RBC), mean corpuscular hemoglobin (MCH), mean corpuscular hemoglobin concentration (MCHC), mean corpuscular volume (MCV), hemoglobin (HGB),

red cell distribution width (RDW-SD), hematocrit (HCT), mean platelet platelet distribution width (PDW), volume (MPV), and lymphocyte (LYM). All of these examinations firmly confirmed that this supramolecular nanomedicine could be safely used in cancer treatment *in vivo*.

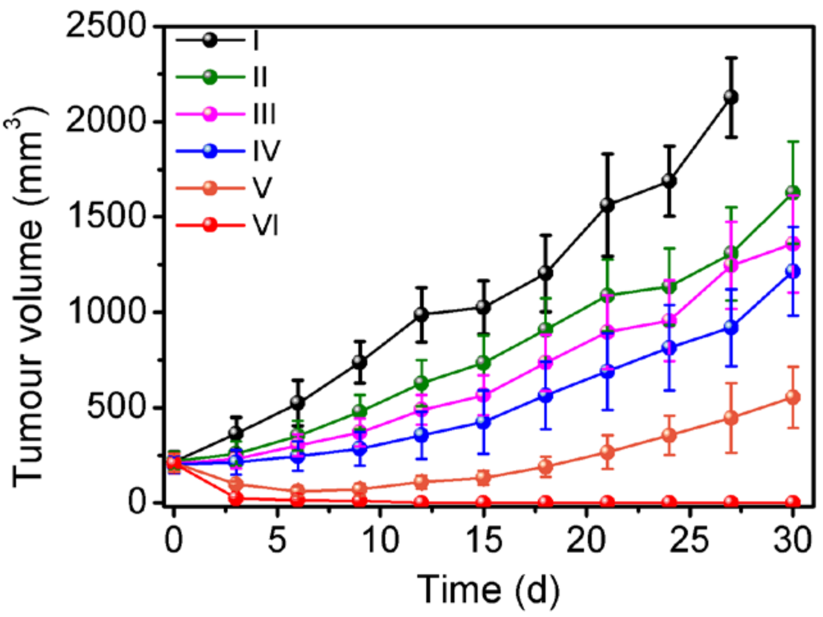

Supplementary Figure 45. (a) Tumour volume changes of the mice bearing HeLa xenografts (around 220 mm^3) treated with different formulations after one injection (n = 8). I, PBS; II, **SCNPs**@PTX (PTX, 20 mg kg^{-1}); III, Abraxane (PTX, 20 mg kg^{-1}); IV, **SCNPs**@PTX (PTX, 60 mg kg^{-1}); V, **SCNPs** + laser (671 nm, 0.5 W cm^{-2}, 10 min); VI, **SCNPs**@PTX (PTX, 60 mg kg^{-1}) + laser (671 nm, 0.5 W cm^{-2}, 10 min). Data are expressed as mean ± s.e.m..

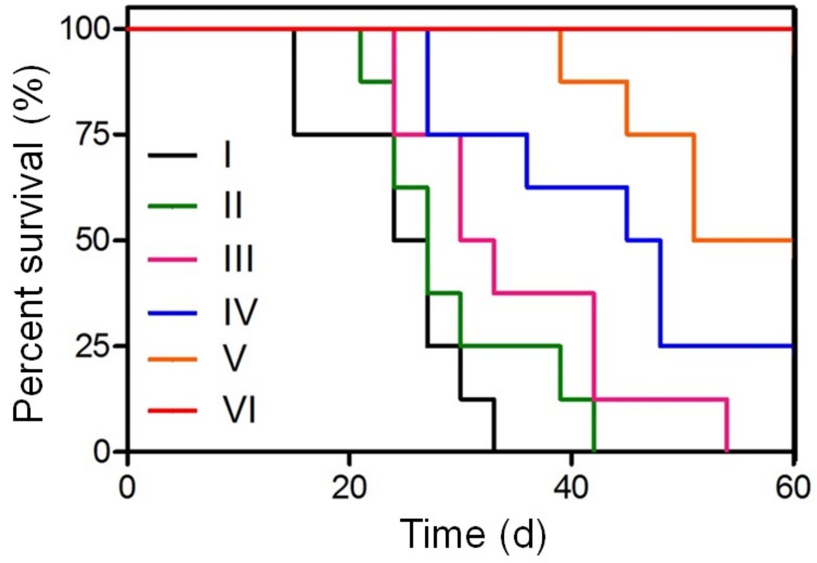

Supplementary Figure 46. Kaplan-Meier survival curves of the mice bearing HeLa xenografts (around 220 mm^3) treated with different formulations after one injection (n = 8). I, PBS; II, **SCNPs**@PTX (PTX, 20 mg kg^{-1}); III,

Abraxane (PTX, 20 mg kg^{-1}); IV, **SCNPs**@PTX (PTX, 60 mg kg^{-1}); V, **SCNPs** + laser (671 nm, 0.5 W cm^{-2}, 10 min); VI, **SCNPs**@PTX (PTX, 60 mg kg^{-1}) + laser (671 nm, 0.5 W cm^{-2}, 10 min).

Our supramolecular nanomedicine (**SCNPs**@PTX) could also completely ablate the tumours with larger size (around 220 mm^3) by taking full advantage of chemotherapy and PTT.

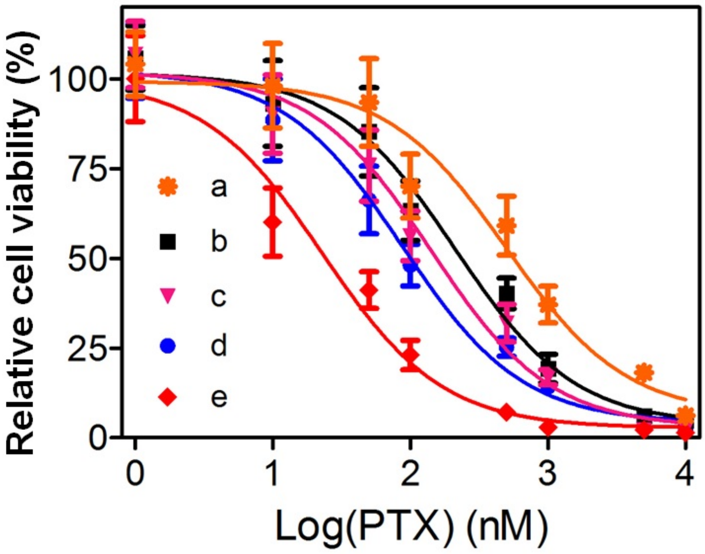

Supplementary Figure 47. *In vitro* cytotoxicity of different formulations toward 4T1 cells: (a) **SCNPs**@PTX pretreated with cRGDfk (20 μM) for 30 min, (b) **SCNPs**@PTX, (c) **SCNPs**@PTX + laser (671 nm, 0.1 W cm^{-2}, 3 min), (d) PTX, (e) **SCNPs**@PTX + laser (671 nm, 0.5 Wcm^{-2}, 3 min). Data are expressed as mean ± s.e.m. of five independent experiments.

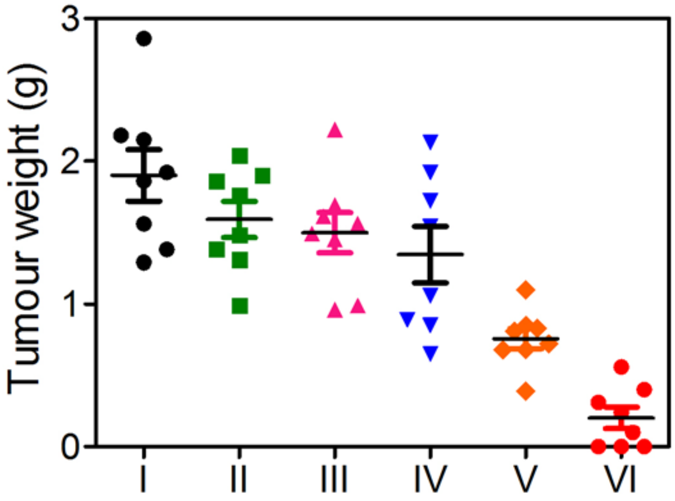

Supplementary Figure 48. Tumour weight from the mice (n = 8) treated with different formulations. I, PBS; II, **SCNPs**@PTX (PTX, 20 mg kg^{-1}); III, Abraxane (PTX, 20 mg kg^{-1}); IV, **SCNPs**@PTX (PTX, 60 mg kg^{-1}); V, **SCNPs** + laser (671 nm, 0.5 W cm^{-2}, 5 min); VI, **SCNPs**@PTX (PTX, 60 mg kg^{-1}) + laser (671 nm, 0.5 W cm^{-2}, 5 min). Data are expressed as mean ± s.e.m..

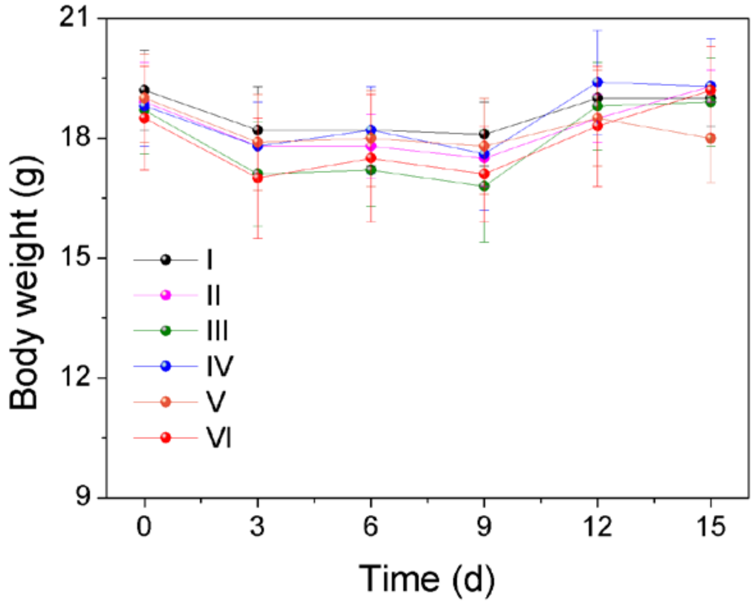

Supplementary Figure 49. Body weight changes of the mice bearing orthotopic 4T1 tumours (n = 8) treated with different formulations. I, PBS; II, **SCNPs**@PTX (PTX, 20 mg kg^{-1}); III, Abraxane (PTX, 20 mg kg^{-1}); IV, **SCNPs**@PTX (PTX, 60 mg kg^{-1}); V, **SCNPs** + laser (671 nm, 0.5 W cm^{-2}, 5 min); VI, **SCNPs**@PTX (PTX, 60 mg kg^{-1}) + laser (671 nm, 0.5 W cm^{-2}, 5 min). Data are expressed as mean ± s.e.m..

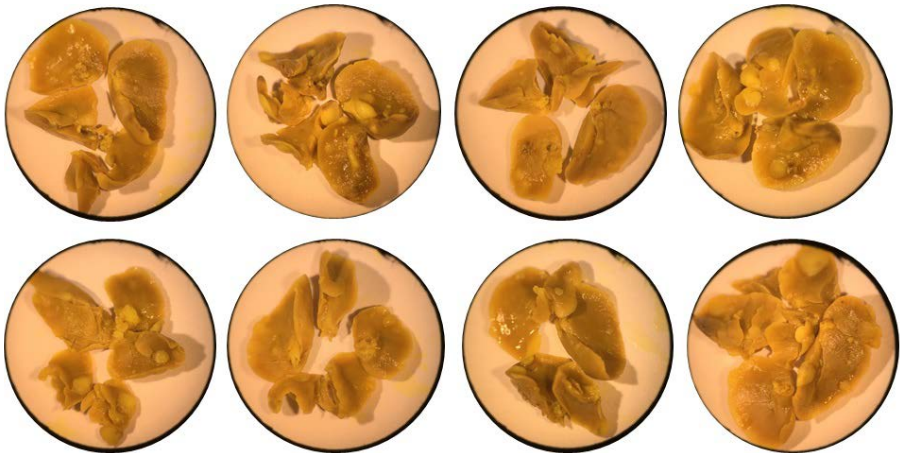

Supplementary Figure 50. Photo images of lung tissues from the mice treated with PBS.

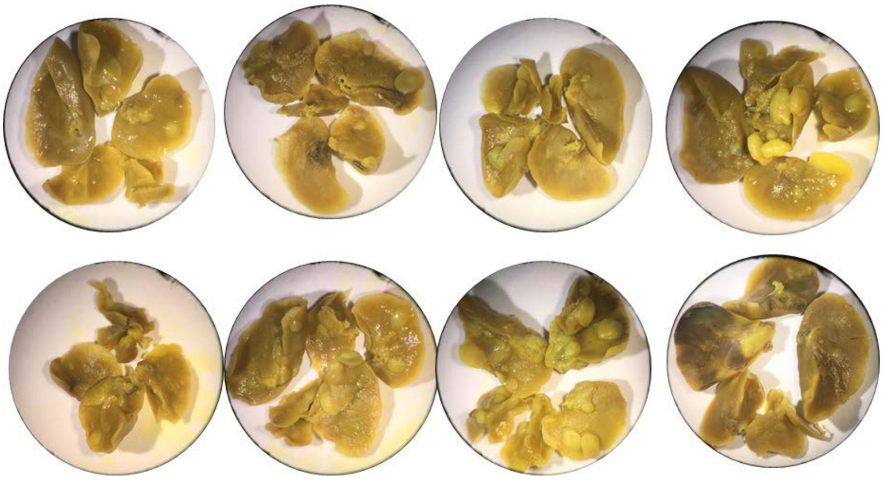

Supplementary Figure 51. Photo images of lung tissues from the mice treated with **SCNPs**@PTX (PTX, 20 mg kg^{-1}).

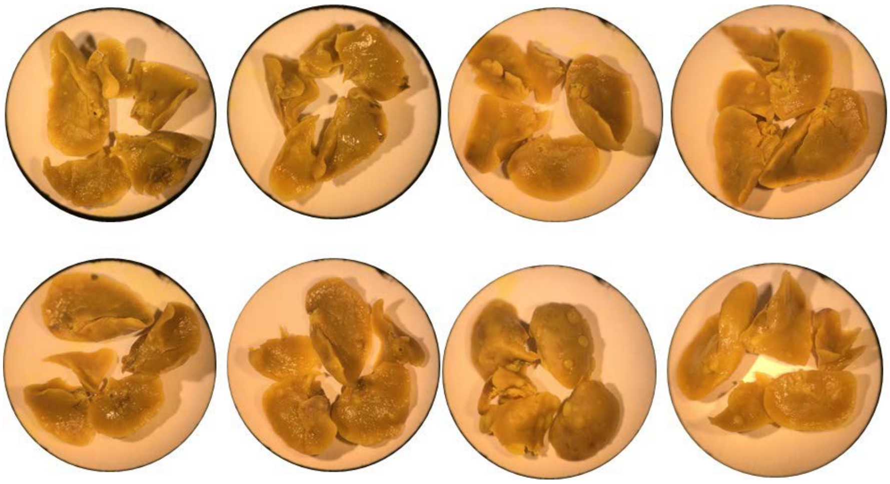

Supplementary Figure 52. Photo images of lung tissues from the mice treated with Abraxane (PTX, 20 mg kg^{-1}).

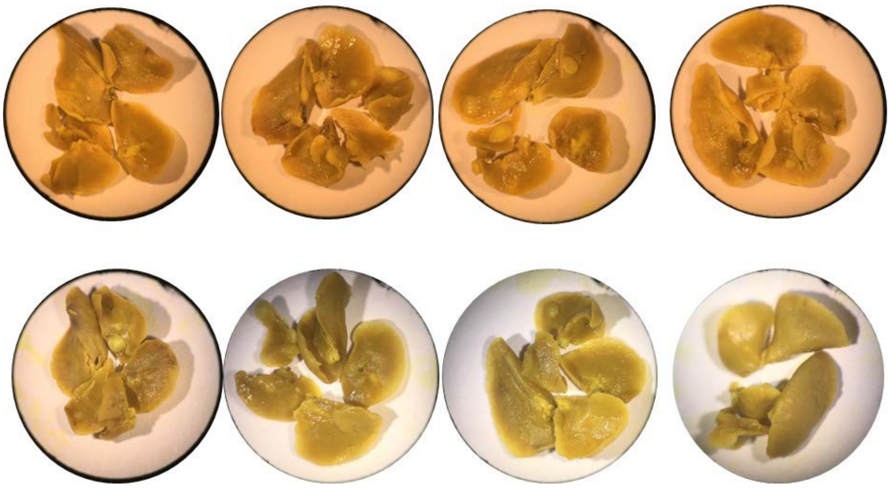

Supplementary Figure 53. Photo images of lung tissues from the mice treated with **SCNPs**@PTX (PTX, 60 mg kg^{-1}).

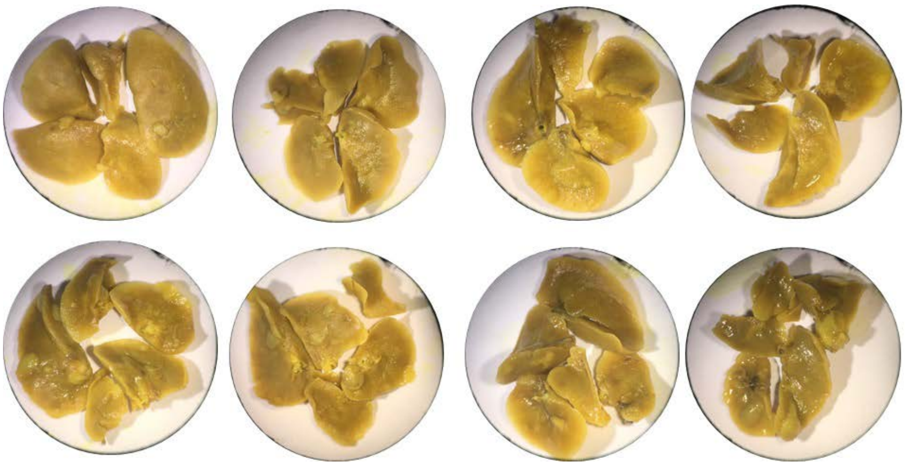

Supplementary Figure 54. Photo images of lung tissues from the mice treated with **SCNPs** + laser (671 nm, 0.5 W cm^{-2}, 5 min).

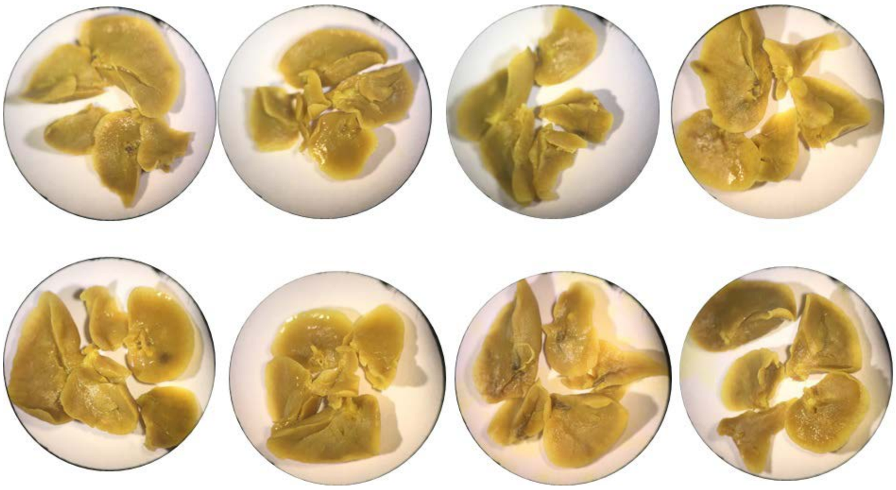

Supplementary Figure 55. Photo images of lung tissues from the mice treated with **SCNPs**@PTX (PTX, 60 mg kg^{-1}) + laser (671 nm, 0.5 W cm^{-2}, 5 min).

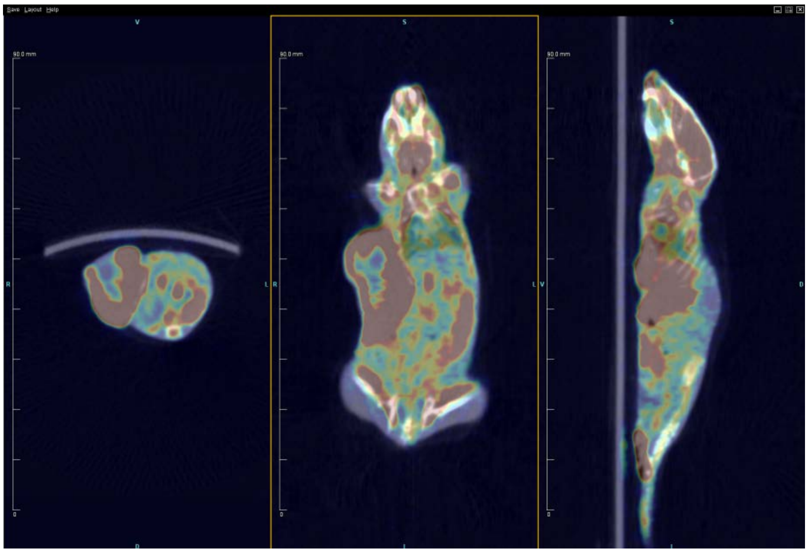

Supplementary Figure 56. PET/CT image of the mouse treated with PBS.

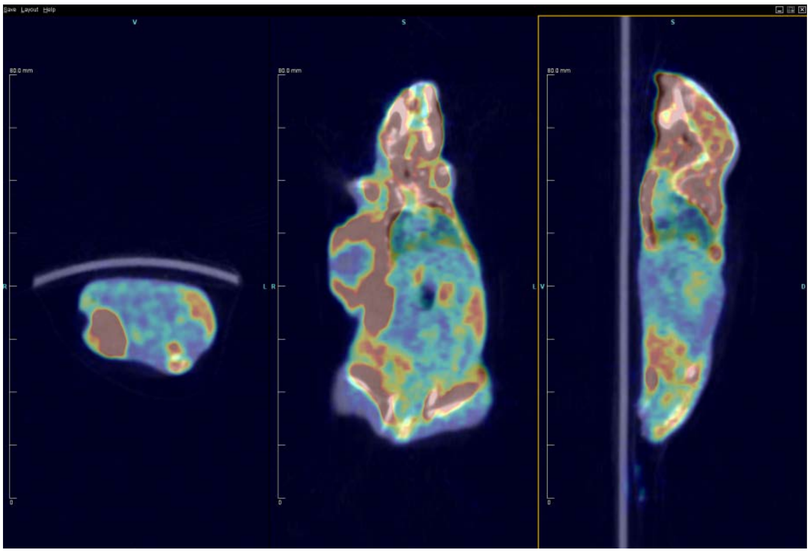

Supplementary Figure 57. PET/CT image of the mouse treated with **SCNPs**@PTX (PTX, 20 mg kg^{-1}).

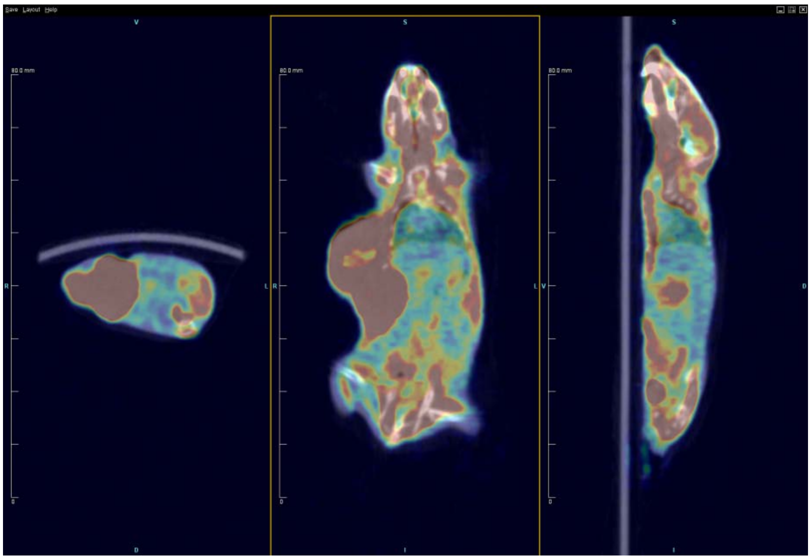

Supplementary Figure 58. PET/CT image of the mouse treated with Abraxane (PTX, 20 mg kg^{-1}).

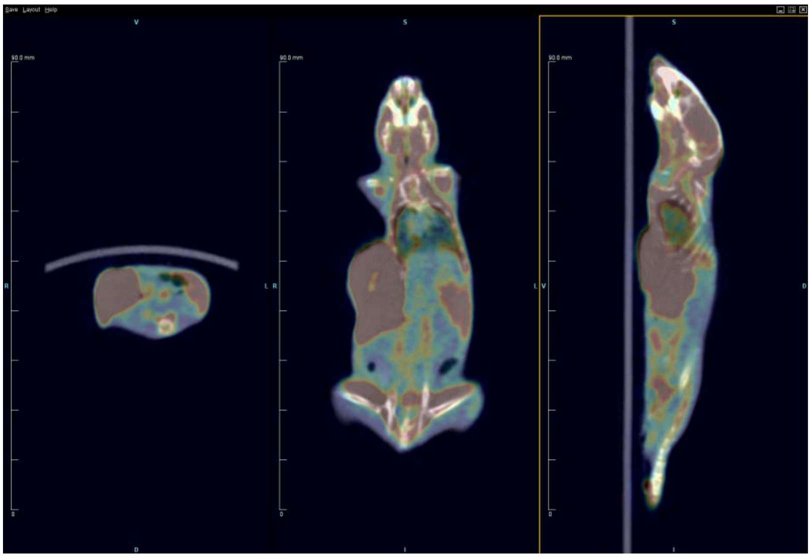

Supplementary Figure 59. PET/CT image of the mouse treated with **SCNPs**@PTX (PTX, 60 mg kg^{-1}).

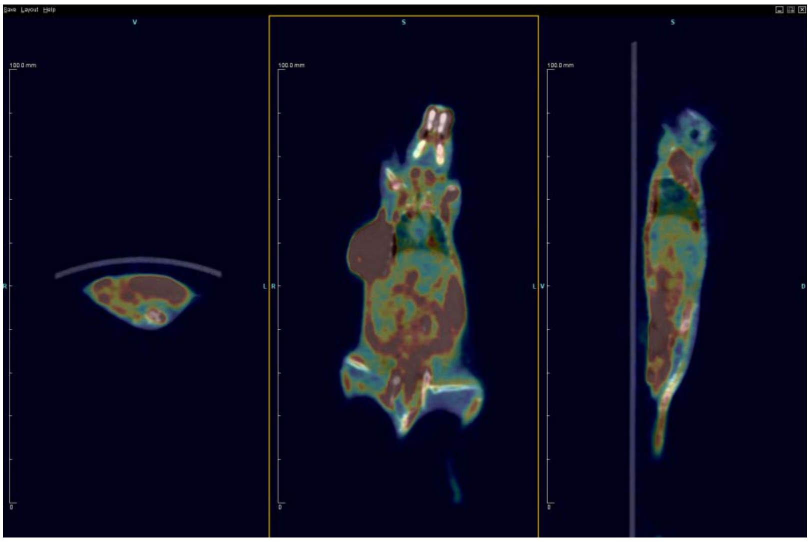

Supplementary Figure 60. PET/CT image of the mouse treated with **SCNPs** + laser (671 nm, 0.5 W cm^{-2}, 5 min).

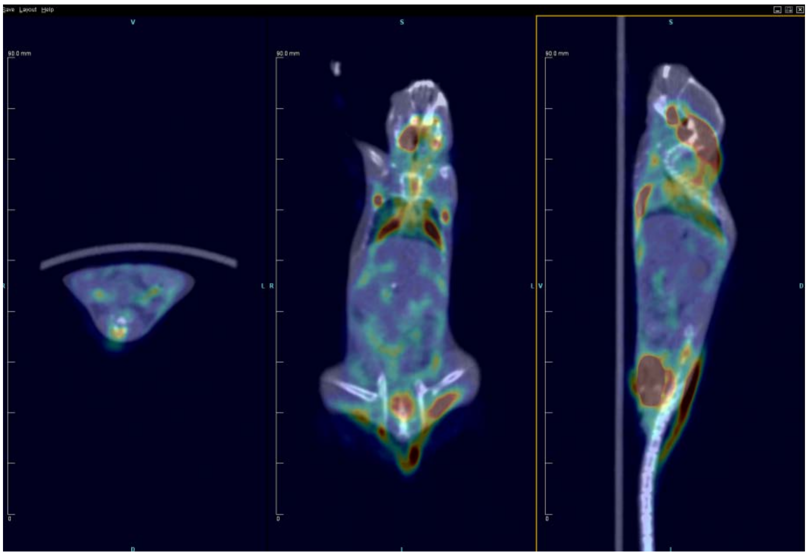

Supplementary Figure 61. PET/CT image of the mouse treated with **SCNPs**@PTX (PTX, 60 mg kg^{-1}) + laser (671 nm, 0.5 W cm^{-2}, 5 min).

*In vitro and in vivo thermo-chemotherapy investigations of **SCNPs**@CPT*

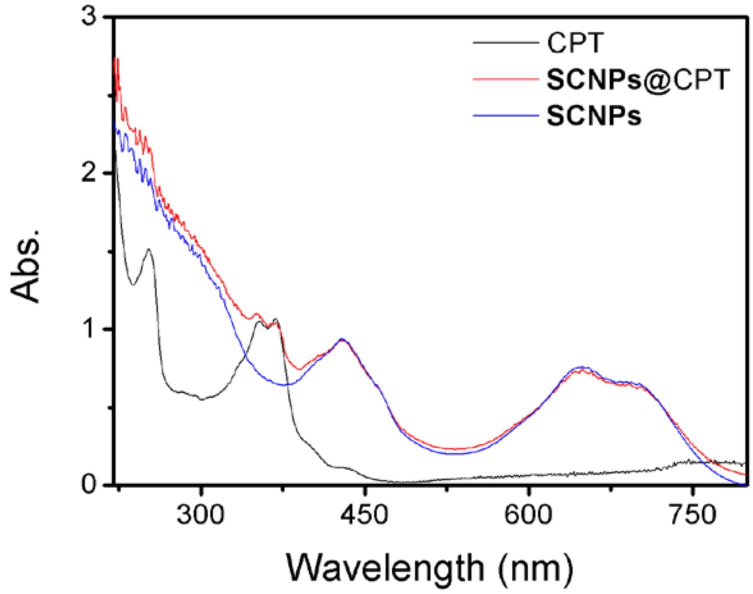

Supplementary Figure 62. UV-vis absorption spectra of CPT, **SCNPs**, and **SCNPs**@CPT.

Supplementary Table 2. Drug Loading Capability and Stability Evaluations.

	Polymer (mg)	CPT (mg)	DLE (%)[a]	DLC (%)[b]	Size (nm)[c]	Stability
Polyrotaxane	100	50	97.7	32.8	128 ± 17.6	No precipitation over 2 d
	100	60	96.6	36.7	134 ± 14.2	No precipitation over 2 d
	100	70	95.5	40.1	147 ± 21.7	No precipitation over 2 d
	100	80	95.0	43.2	152 ± 19.7	No precipitation over 2 d
**PDI-PCL-*b*-PEG-Mal**	100	20	92.1	15.6	124 ± 18.2	No precipitation over 2 d
	100	30	80.2	19.4	174 ± 15.5	No precipitation over 2 d
	100	40	70.3	21.9	275 ± 29.7	Precipitation within 2 h
	100	50	62.3	23.7	449 ± 54.7	Precipitation within 1 h

[a] Drug loading efficient (DLE) = m_{load}/m_{add} * 100%, where m_{add} and m_{load} represent the drug mass added during the preparation of drug-loaded NPs and loaded by the NPs.

[b] Drug loading content (DLC) = $m_{load}/(m_{load} + m)$ * 100%, where m represents the polymer mass used during the preparation of drug-loaded NPs.

[c] The size of the drug-loaded NPs was measured by using DLS.

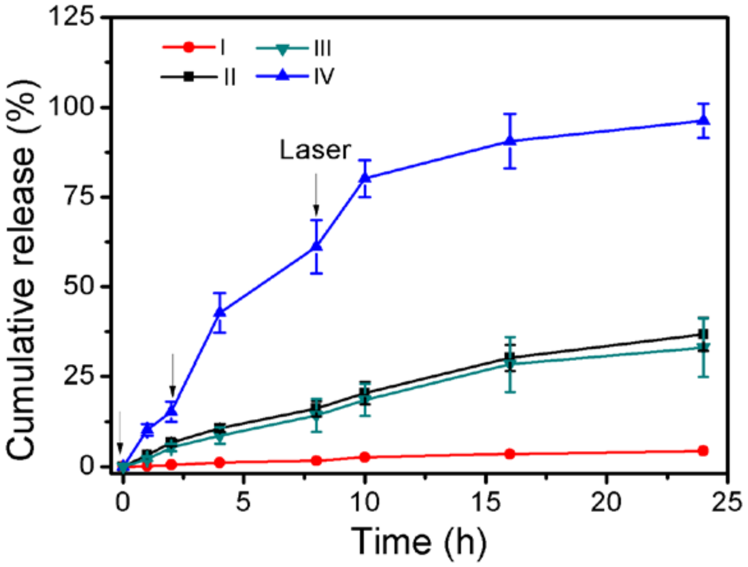

Supplementary Figure 63. Controlled release profiles of (I) **SCNPs**@CPT without any treatments, (II) NPs@CPT, (III) **SCNPs**@CPT in the presence of 10.0 mM GSH, and (IV) **SCNPs**@CPT in the presence of 10.0 mM GSH upon laser irradiation (671 nm, 0.5 W cm^{-2}, 3 min). Data are expressed as mean ± s.e.m. of three independent experiments.

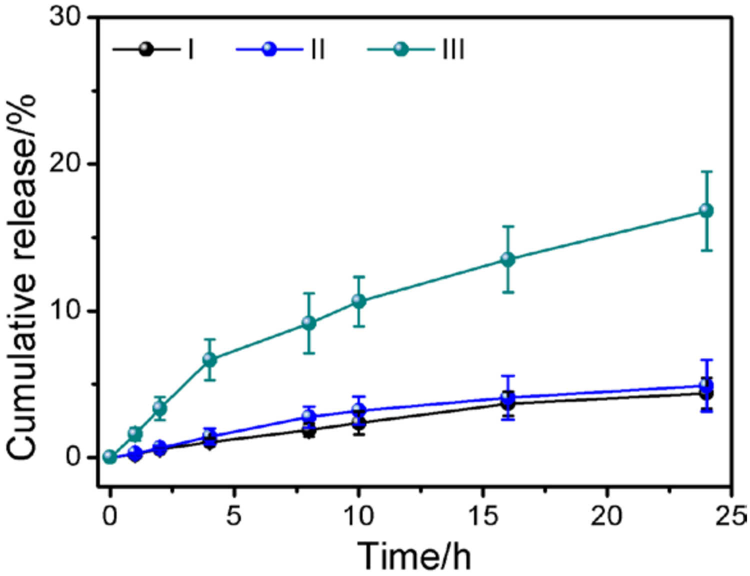

Supplementary Figure 64. Controlled release profiles of (I) **SCNPsCC**@CPT without any treatments, (II) **SCNPsCC**@CPT in the presence of 10.0 mM GSH, and (III) **SCNPsCC**@CPT in the presence of 10.0 mM GSH upon laser irradiation (671 nm, 0.5 W cm^{-2}). Data are expressed as mean ± s.e.m. of three independent experiments.

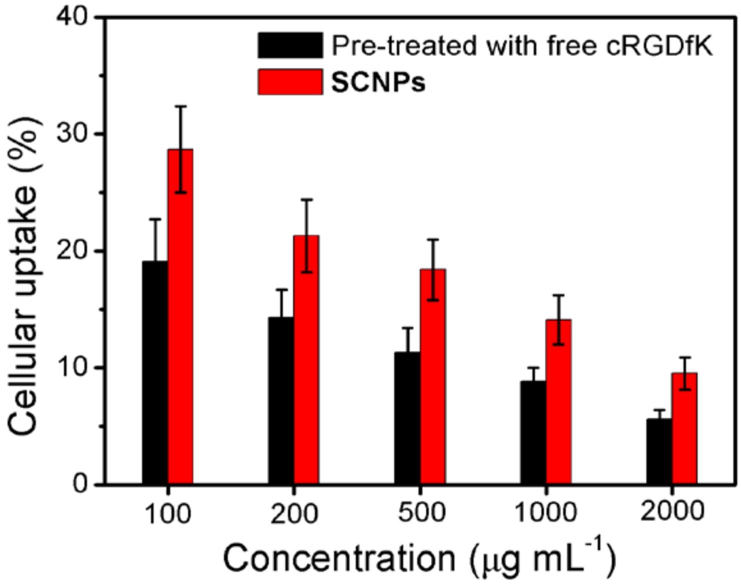

Supplementary Figure 65. Cellular uptake of **SCNPs** by A549 cell line under various concentrations in the absence and presence of free cRGDfK (20 μM) (***P*<0.01). Data are expressed as mean ± s.e.m. of three independent experiments.

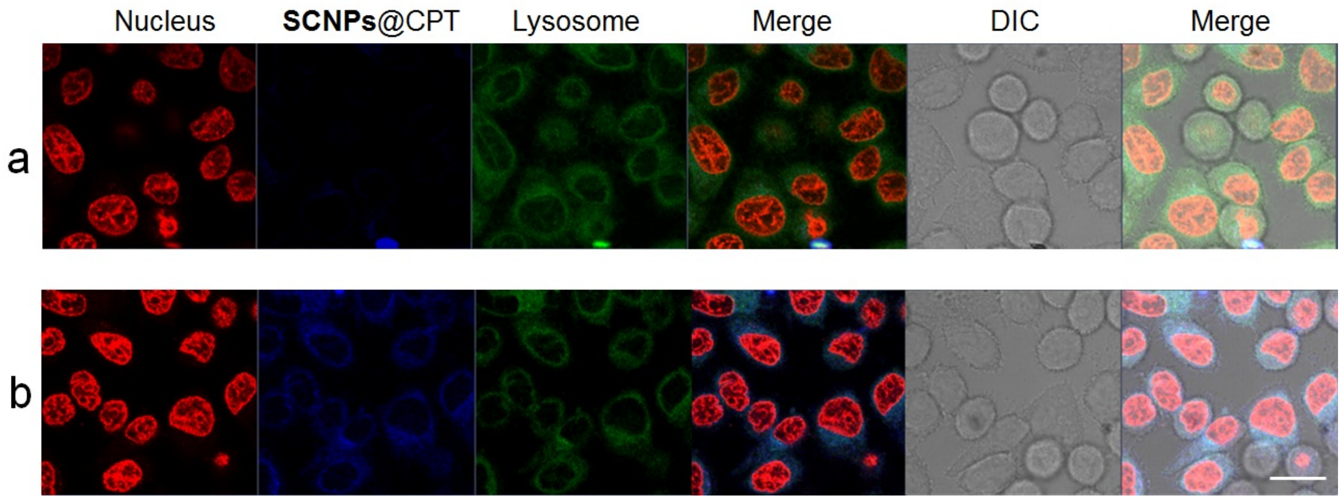

Supplementary Figure 66. CLSM images of the A549 cells incubated with (a) **SCNPs**@CPT pretreated with free cRGDfk (20 μM) for 30 min and (b) **SCNPs**@CPT. Blue fluorescence shows nuclear staining with DOX·HCl for 24 h; blue fluorescence shows the location of **SCNPs**@CPT; green fluorescence shows lysosome staining with Lyso tracker Green (0.10 μM). Scale bar is 20 μm.

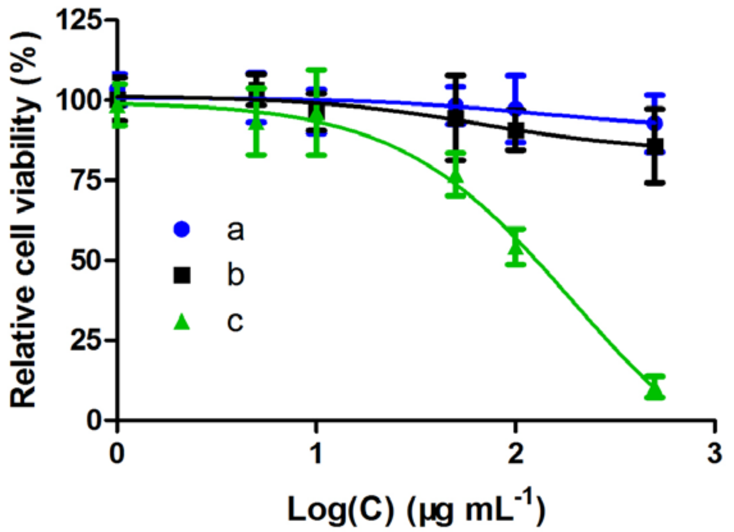

Supplementary Figure 67. Relative cell viability of A549 cells incubated with various concentrations of (a) **SCNPs**, (b) **SCNPs** + laser (671 nm, 0.1 W cm^{-2}, 3 min), and (c) **SCNPs** + laser (671 nm, 0.5 W cm^{-2}, 3 min). Data are expressed as mean ± s.e.m. of five independent experiments.

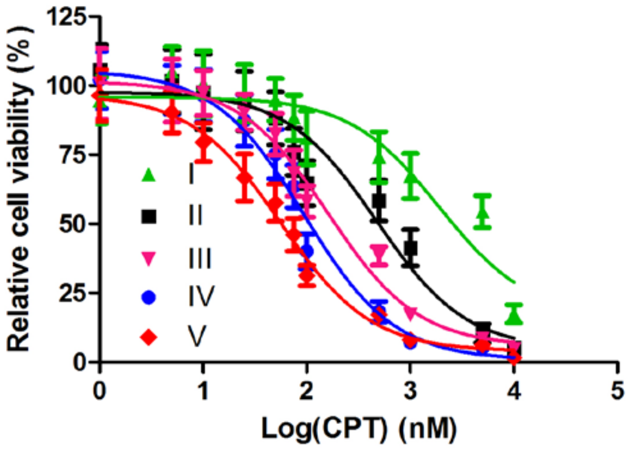

Supplementary Figure 68. *In vitro* cytotoxicity of different formulations toward A549 cells: I, **SCNPsCC**@CPT; II, **SCNPs**@CPT; III, **SCNPs**@CPT + laser (671 nm, 0.1 W cm^{-2}, 3 min); IV, CPT; V, **SCNPs**@CPT + laser (671 nm, 0.5 W cm^{-2}, 3 min). Data are expressed as mean ± s.e.m. of five independent experiments.

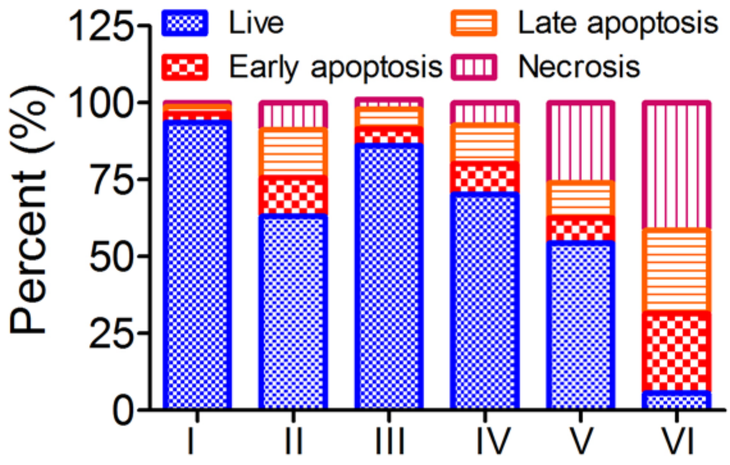

Supplementary Figure 69. Flow-cytometric analysis of annexin-V/PI staining of A549 cells after different formulations: I, control; II, CPT (50 nM); III, **SCNPsCC**@CPT (CPT, 50 nM); IV, **SCNPs**@CPT (50 nM); V, **SCNPs** + laser (671 nm, 0.5 W cm^{-2}, 3 min); VI, **SCNPs**@CPT (CPT, 50 nM) + laser (671 nm, 0.5 W cm^{-2}, 3 min).

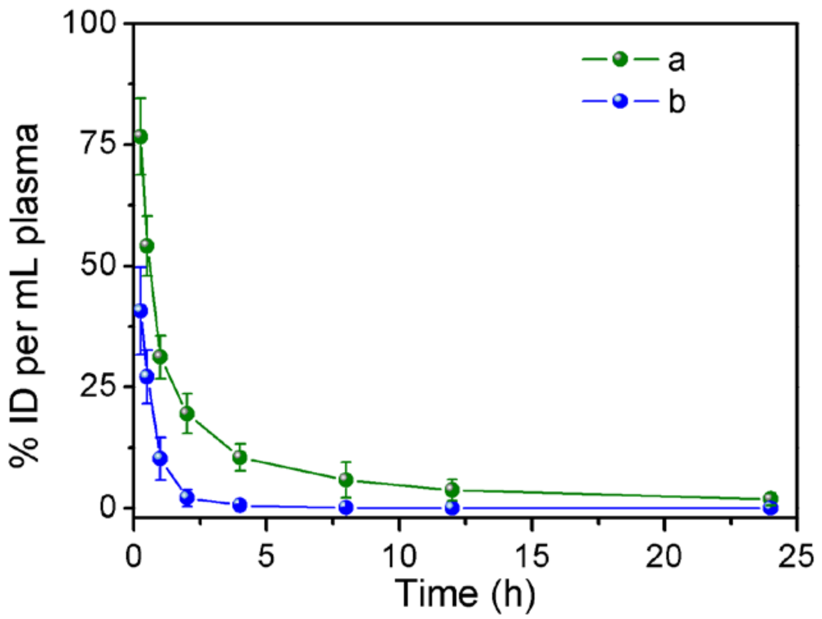

Supplementary Figure 70. *In vivo* blood elimination kinetics of (a) **SCNPs**@CPT and (b) CPT (n = 3 for each group).

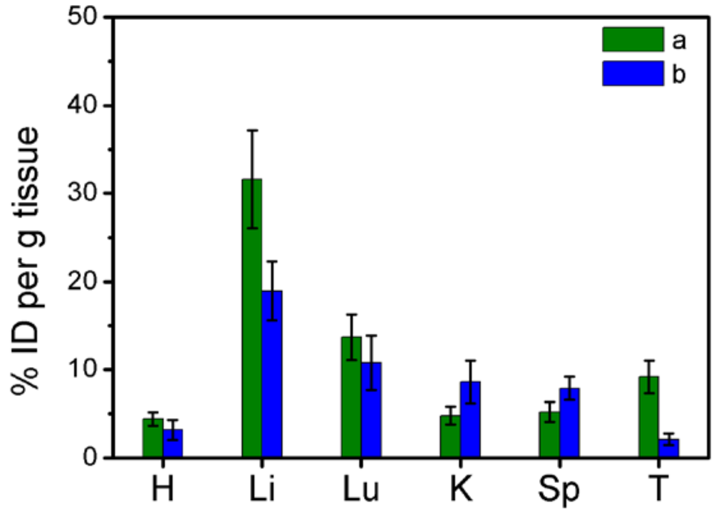

Supplementary Figure 71. Tissue distributions in the main organs after *i.v.* injection of (a) **SCNPs**@CPT and (b) CPT at 24 h post injection. (H: heart, Li: liver, Lu: lung, Sp: spleen, K: kidney, T: tumour). Data are expressed as mean ± s.e.m. of three independent experiments.

Supplementary Table 3. Treatment Response for Maximum Tolerable Dose (MTD) Study

	Dose (mg/kg)[a]	No. of nude mice	max % wt loss (day)[b]	Death (day)
CPT	5.00	6	4.91 (5)	0
	10.0	6	9.74 (6)	0
	15.0	6	14.2 (6)	1 (2)
	20.0	6	18.4 (5)	2 (3)<br>1 (4)<br>culled[c]
**SCNPs**@CPT	10.0	6	6.53 (8)	0
	20.0	6	9.76 (6)	0
	25.0	6	12.1 (6)	0
	30.0	6	14.2 (8)	1 (4)
	35.0	6	19.4 (10)	1 (3)<br>1 (4)<br>culled<br>culled

[a] The groups (CPT and **SCNPs**@CPT) containing CPT are based on mg CPT per kg, the blank control (**SCNPs**) is based on mg **SCNPs** per kg.

[b] Maximum percent body weight loss.

[c] Animals culled due to exceeding 15% body weight loss.

[d] No decrease in body weight was observed during the experimental period.

As discussed above, **SCNPs** exhibited excellent structural stability under physiological conditions, of which the encapsulated drugs were able to be retained, with minimal premature release and

dissociation of the NPs. On the other hand, high tumour accumulation was achieved by taking advantage of the EPR effect and active targeting, effectively reducing side effects towards normal tissues. Therefore, the injected dose of **SCNPs**@CPT could be increased to promote anti-tumour efficacy.

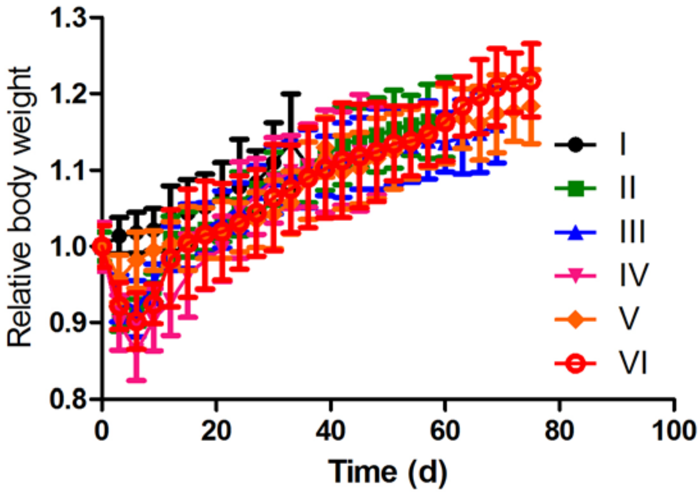

Supplementary Figure 72. Body weight changes of the mice treated with different formulations. I, PBS; II, CPT (5 mg kg^{-1}); III, **SCNPs**@CPT (CPT, 5 mg kg^{-1}); IV, **SCNPs**@CPT (CPT, 15 mg kg^{-1}); V, **SCNPs** + laser (671 nm, 0.5 W cm^{-2}, 5 min); VI, **SCNPs**@CPT (CPT, 15 mg kg^{-1}) + laser (671 nm, 0.5 W cm^{-2}, 5 min). Data are expressed as mean ± s.e.m..

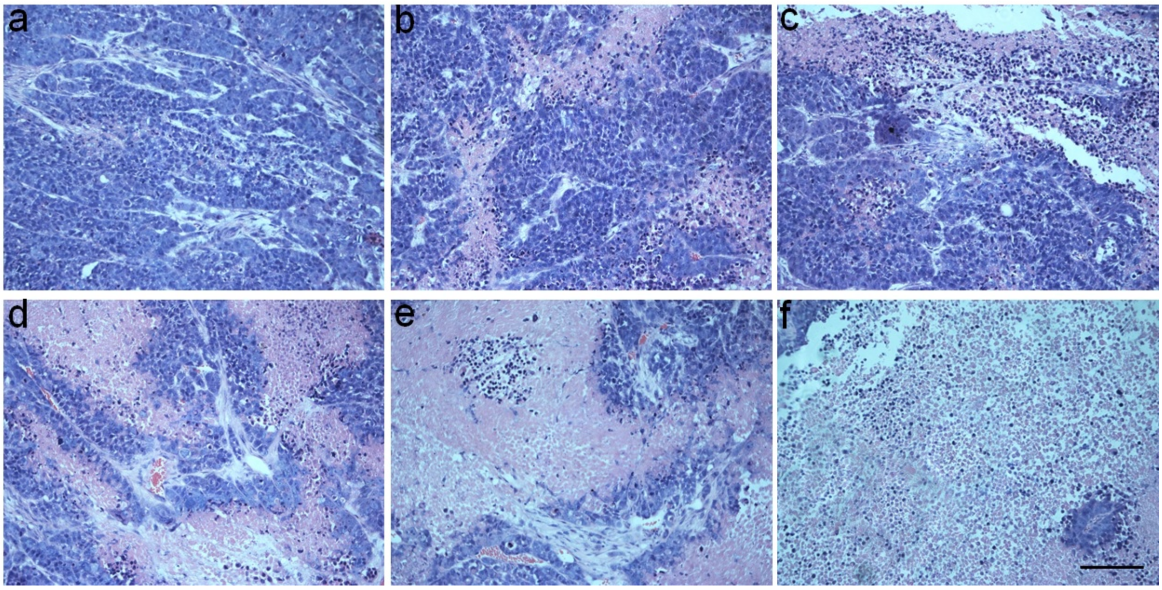

Supplementary Figure 73. H&E analyses of tumour tissues after treatment with various formulations: (a) PBS, (b) CPT (5 mg kg^{-1}), (c) **SCNPs**@CPT (CPT, 5 mg kg^{-1}), (d) **SCNPs**@CPT (CPT, 15 mg kg^{-1}), (e) **SCNPs** + laser (671 nm, 0.5 W cm^{-2}, 5 min), (f) **SCNPs**@CPT (CPT, 15 mg kg^{-1}) + laser (671 nm, 0.5 W cm^{-2}, 5 min). Scale bar is 100 μm.

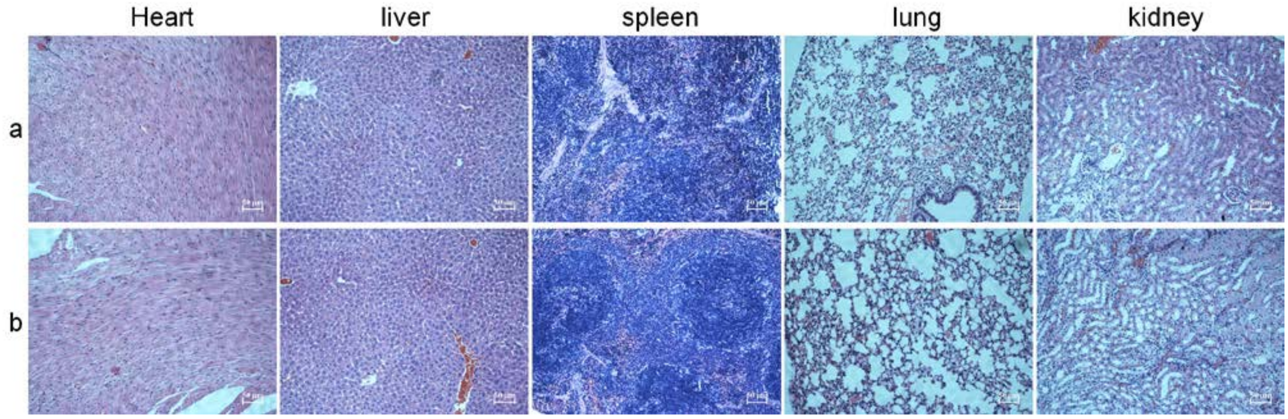

Supplementary Figure 74. H&E stained images of heart, liver, spleen, lung and kidney from different groups after treatment with various formulations: (a) PBS and (b) **SCNPs**@CPT (CPT, 15 mg kg^{-1}) + laser (671 nm, 0.5 W cm^{-2}, 5 min).

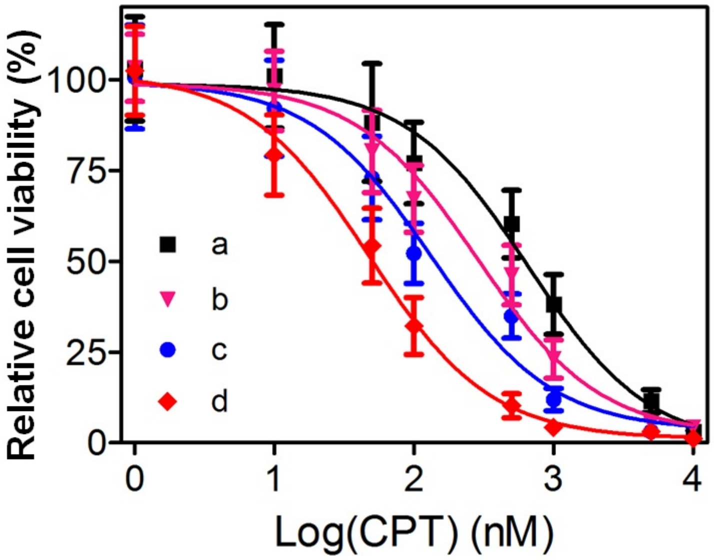

Supplementary Figure 75. *In vitro* cytotoxicity of different formulations toward 4T1 cells: (a) **SCNPs**@CPT, (b) **SCNPs**@CPT + laser (671 nm, 0.1 W cm^{-2}, 3 min), (c) CPT, (d) **SCNPs**@CPT + laser (671 nm, 0.5 W cm^{-2}, 3 min). Data are expressed as mean ± s.e.m. of five independent experiments.

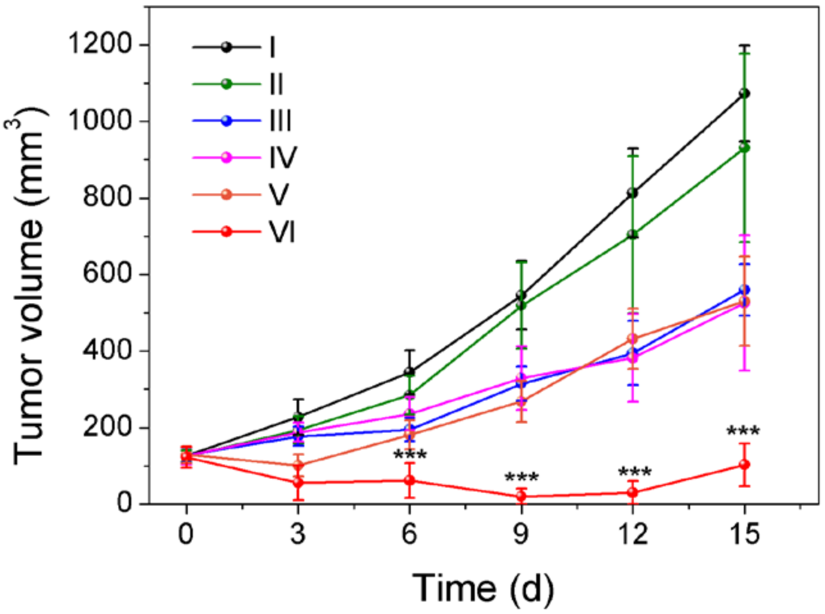

Supplementary Figure 76. Tumour volume change of the mice bearing orthotopic 4T1 tumours treated with different formulations after one injection (n = 8). I, PBS; II, CPT (5 mg kg^{-1}); III, **SCNPs**@CPT (CPT, 5 mg kg^{-1}); IV, **SCNPs**@CPT (CPT, 15 mg kg^{-1}); V, **SCNPs** + laser (671 nm, 0.5 W cm^{-2}, 5 min); VI, **SCNPs**@CPT (CPT, 15 mg kg^{-1}) + laser (671 nm, 0.5 W cm^{-2}, 5 min). Data are expressed as means ± s.e.m., ***P<0.001.

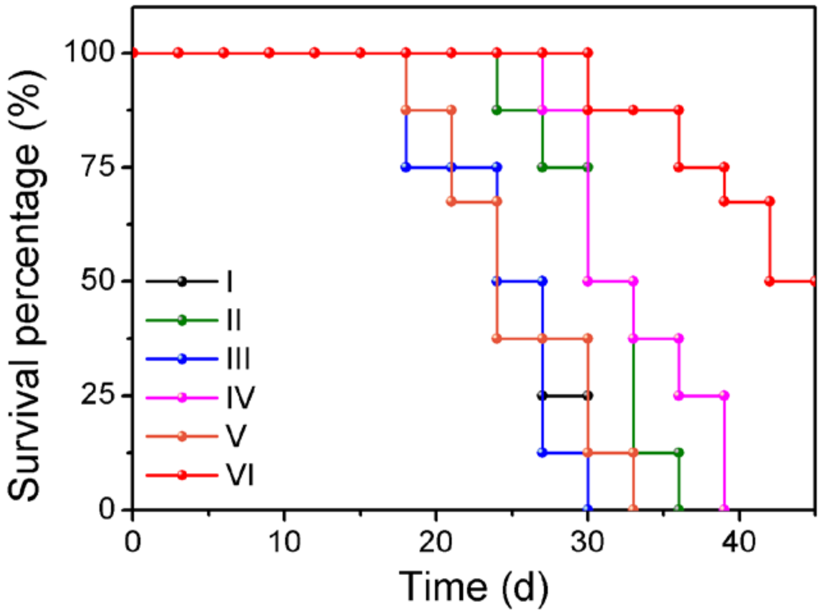

Supplementary Figure 77. Kaplan-Meier survival curves of the mice bearing orthotopic 4T1 tumour treated with different formulations after one injection (n = 8). I, PBS; II, CPT (5 mg kg^{-1}); III, **SCNPs**@CPT (CPT, 5 mg kg^{-1}); IV, **SCNPs**@CPT (CPT, 15 mg kg^{-1}); V, **SCNPs** + laser (671 nm, 0.5 W cm^{-2}, 5 min); VI, **SCNPs**@CPT (CPT, 15 mg kg^{-1}) + laser (671 nm, 0.5 W cm^{-2}, 5 min).

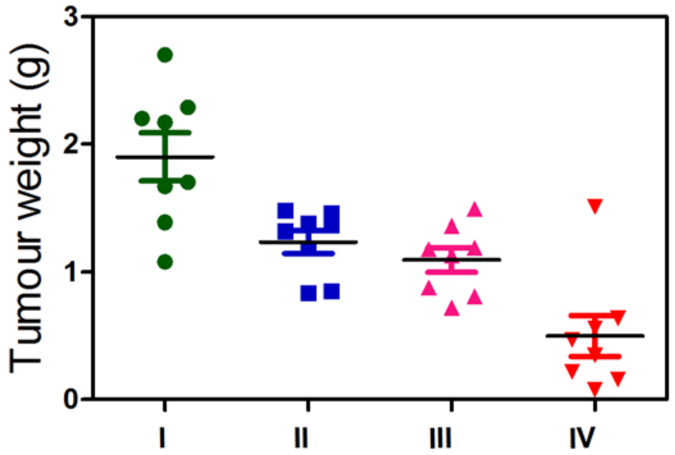

Supplementary Figure 78. Tumour weight from the mice treated with different formulations. I, CPT (5 mg kg^{-1}); II, **SCNPs**@CPT (CPT, 5 mg kg^{-1}); III, **SCNPs**@CPT (CPT, 15 mg kg^{-1}); IV, **SCNPs**@CPT (CPT, 15 mg kg^{-1}) + laser (671 nm, 0.5 W cm^{-2}, 5 min). Data are expressed as mean ± s.e.m..

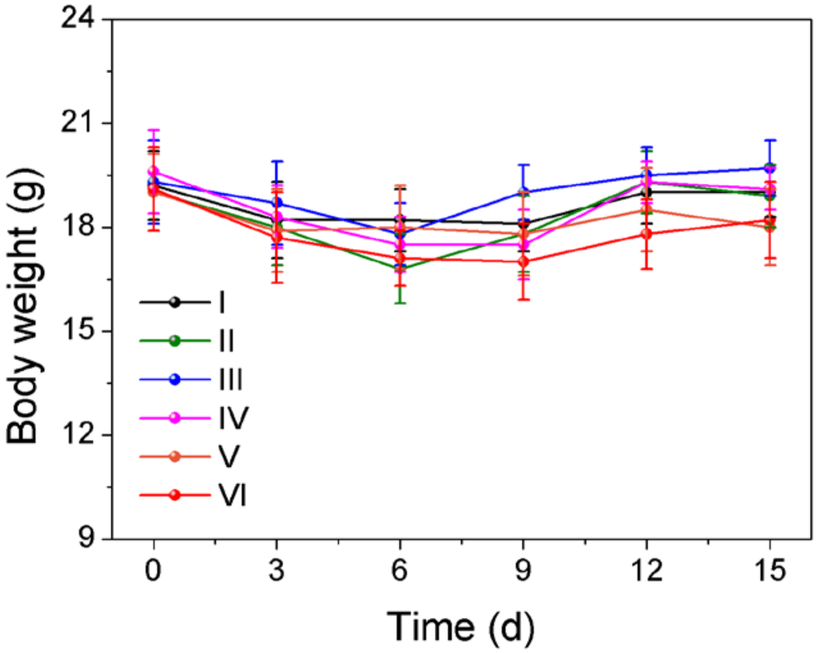

Supplementary Figure 79. Body weight changes of the mice bearing orthotopic 4T1 tumour treated with different formulations. I, PBS; II, CPT (5 mg kg^{-1}); III, **SCNPs**@CPT (CPT, 5 mg kg^{-1}); IV, **SCNPs**@CPT (CPT, 15 mg kg^{-1}); V, **SCNPs** + laser (671 nm, 0.5 W cm^{-2}, 5 min); VI, **SCNPs**@CPT (CPT, 15 mg kg^{-1}) + laser (671 nm, 0.5 W cm^{-2}, 5 min). Data are expressed as mean ± s.e.m..

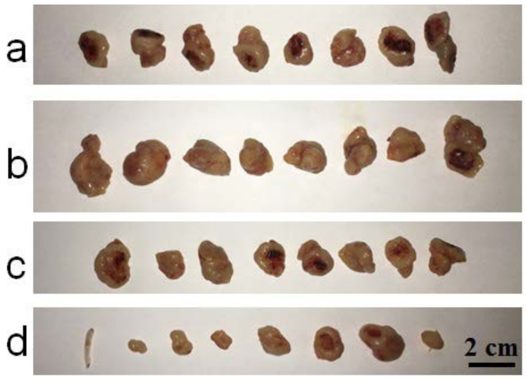

Supplementary Figure 80. Representative images of the tumours harvested from the mice treated with (a) CPT (5 mg kg^{-1}), (b) **SCNPs**@CPT (CPT, 5 mg kg^{-1}), (c) **SCNPs**@CPT (CPT, 15 mg kg^{-1}), and (d) **SCNPs**@CPT (CPT, 15 mg kg^{-1}) + laser (671 nm, 0.5 W cm^{-2}, 5 min).

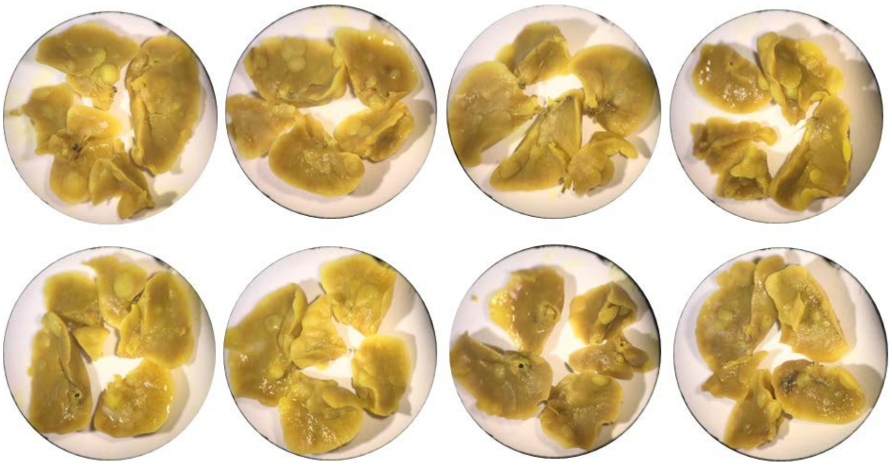

Supplementary Figure 81. Photo images of lung tissues from the mice treated with CPT (CPT, 5 mg kg^{-1}).

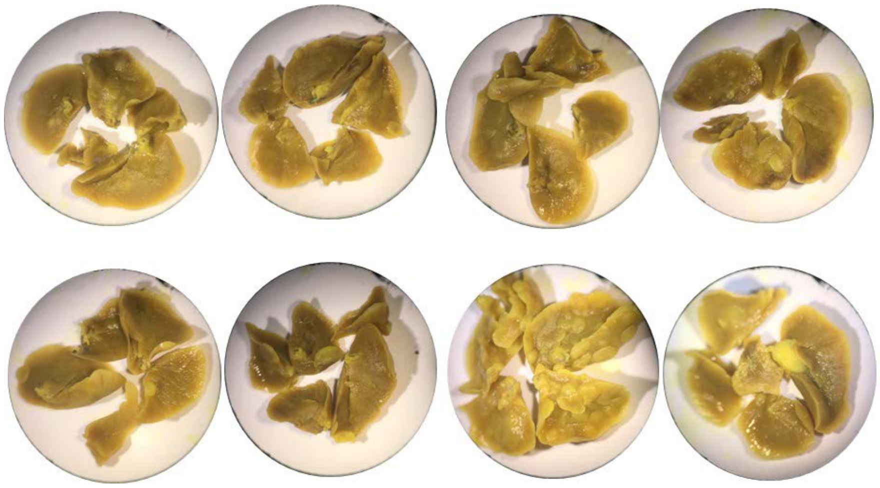

Supplementary Figure 82. Photo images of lung tissues from the mice treated with **SCNPs**@CPT (CPT, 5 mg kg^{-1}).

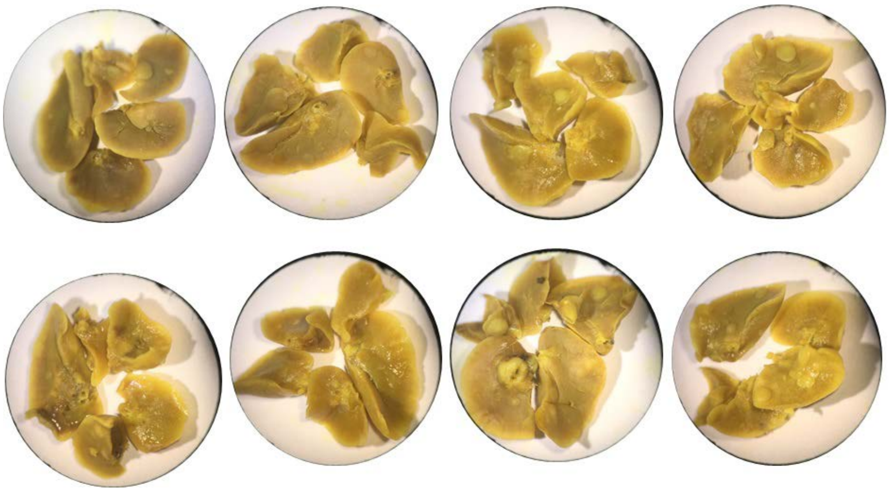

Supplementary Figure 83. Photo images of lung tissues from the mice treated with **SCNPs**@CPT (CPT, 15 mg kg^{-1}).

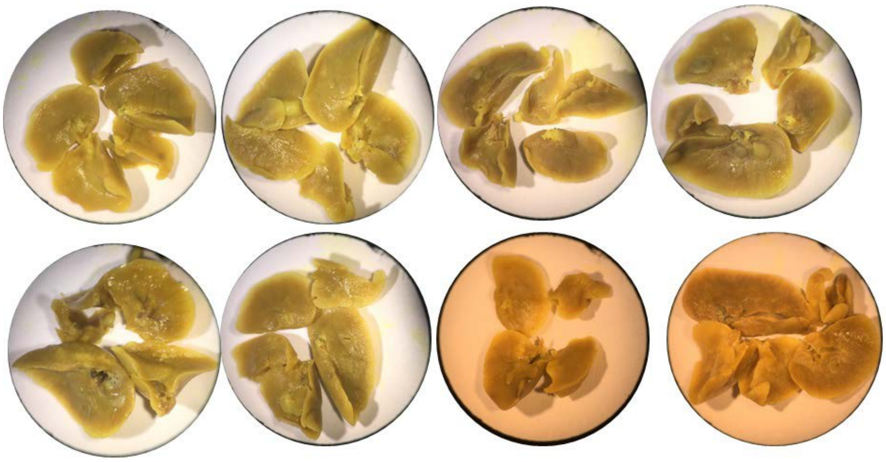

Supplementary Figure 84. Photo images of lung tissues from the mice treated with **SCNPs**@CPT (CPT, 15 mg kg^{-1}) + laser (671 nm, 0.5 W cm^{-2}, 5 min).

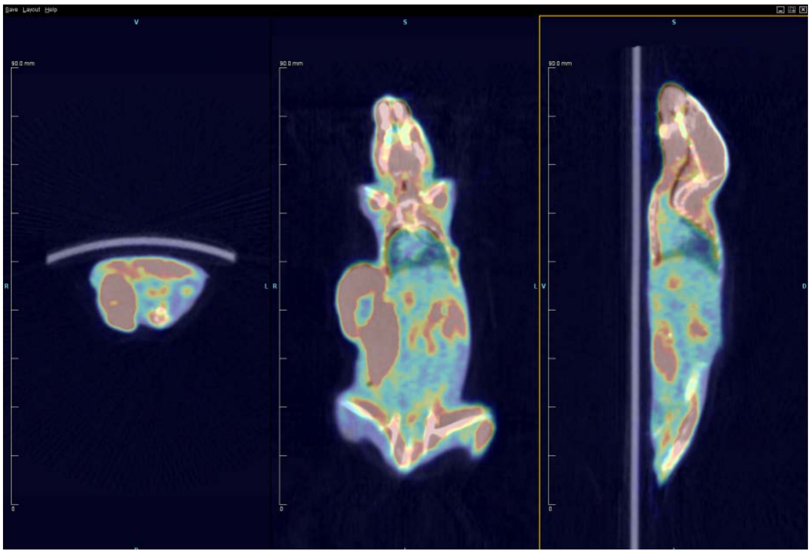

Supplementary Figure 85. PET/CT image of the mouse treated with CPT (CPT, 5 mg kg^{-1}).

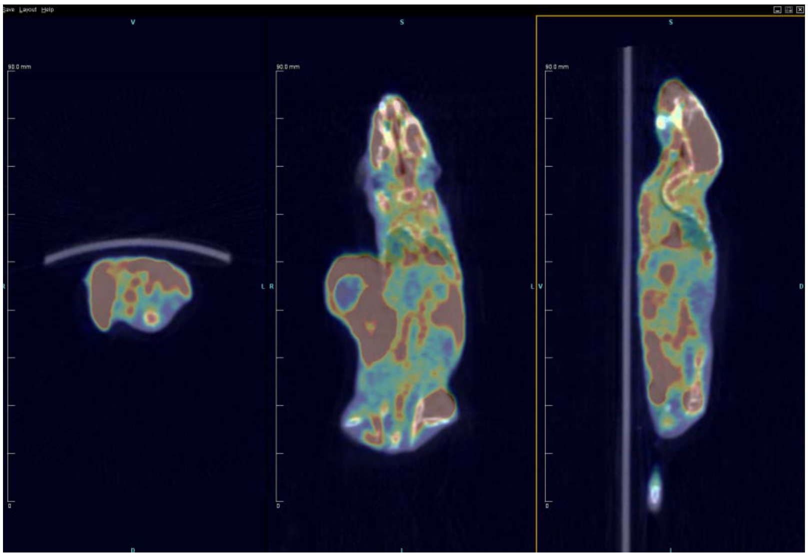

Supplementary Figure 86. PET/CT image of the mouse treated with **SCNPs**@CPT (CPT, 5 mg kg^{-1}).

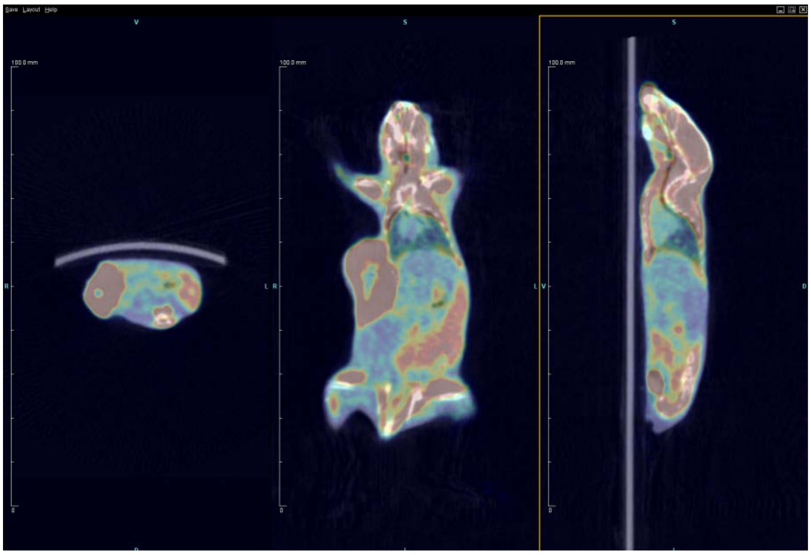

Supplementary Figure 87. PET/CT image of the mouse treated with **SCNPs**@CPT (CPT, 15 mg kg^{-1}).

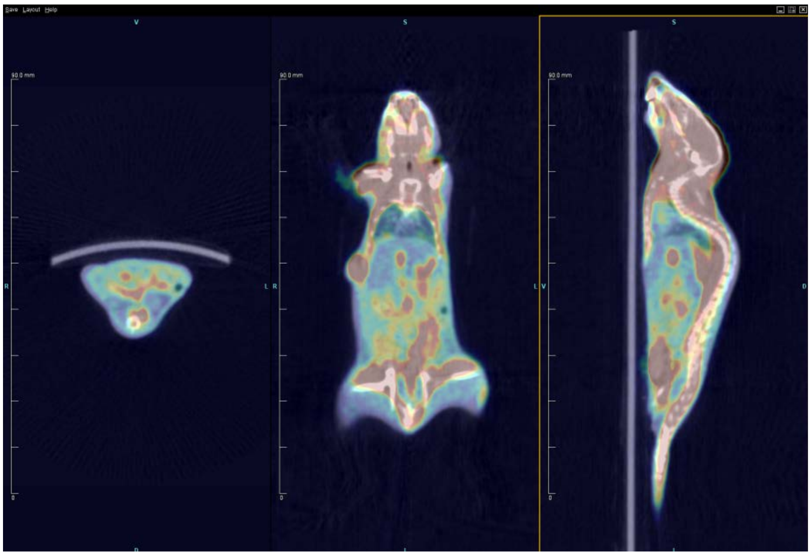

Supplementary Figure 88. PET/CT image of the mice mouse with **SCNPs**@CPT (CPT, 15 mg kg^{-1}) + laser (671 nm, 0.5 W cm^{-2}, 5 min).

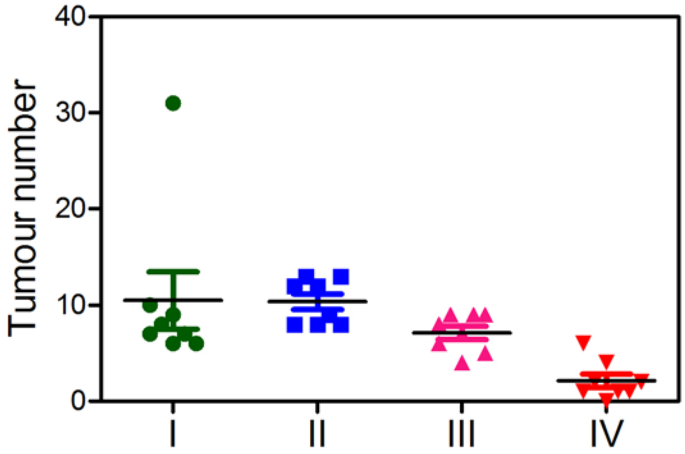

Supplementary Figure 89. Metastasis nodules in lung tissue from the mice treated with different administrations. (I) CPT (5 mg kg^{-1}), (II) **SCNPs**@CPT (CPT, 5 mg kg^{-1}), (III) **SCNPs**@CPT (CPT, 15 mg kg^{-1}), (IV) **SCNPs**@CPT (CPT, 15 mg kg^{-1}) + laser (671 nm, 0.5 W cm^{-2}, 5 min). Data are expressed as mean ± s.e.m..

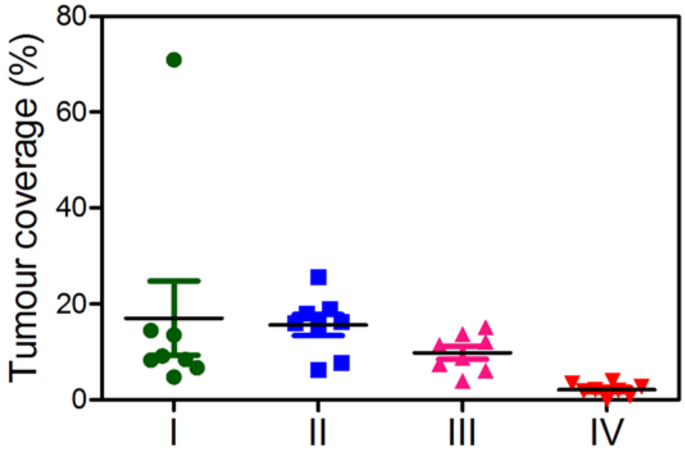

Supplementary Figure 90. Tumour coverage in lung tissue from the mice treated with different administrations. (I) CPT (5 mg kg^{-1}), (II) **SCNPs**@CPT (CPT, 5 mg kg^{-1}), (III) **SCNPs**@CPT (CPT, 15 mg kg^{-1}), (IV) **SCNPs**@CPT (CPT, 15 mg kg^{-1}) + laser (671 nm, 0.5 W cm^{-2}, 5 min). Data are expressed as mean ± s.e.m..

Apart from hydrophobic interactions, π-π stacking interactions could also be realized between PDI groups and planar CPT in **SCNPs**, which was favorable to enhance its loading capacity. Almost identical release behavior for **SCNPs**@CPT as that of **SCNPs**@PTX (Supplementary Fig. 63-64), except that the release rate was slightly lower because of the additional π-π stacking interactions between CPT and PDI groups located in the core of **SCNPs**, confirming that **SCNPs** could be utilized as a robust drug delivery platform. The average diameter of **SCNPs**@CPT was measured to be 152 nm by DLS (Supplementary Fig. 26), and the morphology was revealed by TEM imaging (Supplementary Fig. 21c). CLSM and cellular internalization experiments showed that the uptake of **SCNPs**@CPT was effectively blocked by

pre-treatment with free cRGDfK (20 μM), thus demonstrating the presence of cRGDfK on the surface of **SCNPs** was favourable for endocytosis by A549 cells overexpressing $\alpha_v\beta_3$ integrin receptor (Supplementary Fig. 65-66). The IC_{50} values were calculated to be 94.6 ± 8.43, 2018 ± 264, 472± 60.7, 162 ± 14.7, and 56.2 ± 8.96 nM, for the cells treated with CPT, **SCNPsCC**@CPT, **SCNPs**@CPT, **SCNPs**@CPT + laser (0.1 W cm^{-2}, 3 min), and **SCNPs**@CPT + laser (0.5 W cm^{-2}, 3 min), respectively, confirming the optimal therapeutic effect due to their synergetic PTT and chemotherapy (Supplementary Fig. 68-69). The pharmacokinetics and biodistribution investigations proved that the blood circulation time and tumour accumulation of **SCNPs**@CPT was greatly increased, in comparison with free CPT (Supplementary Fig. 70-71), attributing to the EPR effect and active targeting capability. Compared with free CPT (10 mg kg^{-1}), the MTD value of **SCNPs**@CPT significantly increased to 25 mg kg^{-1}, benefiting from the shell crosslinking strategy (Supplementary Table 3). *In vivo* anti-tumour efficacy of **SCNPs**@CPT was tested in a A549 xenograft model (Fig. 7c). Complete remission of A549 tumours was achieved with a single injection of high dose **SCNPs**@CPT (CPT, 15 mg kg^{-1}) plus laser irradiation without losses in body weight (Supplementary Fig. 72). While in the other groups, tumour recurrance occurred for the mice treated wtih free CPT (5 mg kg^{-1}), low dose **SCNPs**@CPT (CPT, 5 mg kg^{-1}), high dose **SCNPs**@CPT (CPT, 15 mg kg^{-1}), and **SCNPs** + laser. H&E staining of the tumour tissues and main organs confirmed that the synergetic thermo-chemotherapy was advantageous over PTT or chemotherapy alone, with no signs of systemic toxicity (Supplementary Fig. 73-74). In comparison with the other control groups, the median survival rate of the mice treated with **SCNPs**@CPT (CPT, 15 mg kg^{-1}) increased significantly (Fig. 7d), benefiting from the synergistic anticancer efficacy and low systemic toxicity. Orthotopic 4T1 tumour model also demonstrated that combination of chemotherapy with high dosage and PTT (**SCNPs**@CPT + laser irradiation) significantly suppressed primary tumour growth and prevented lung metastasis more effectively than either chemotherapy or PTT alone (Supplementary Fig. 75–90).

References:

S1. Tian, Q. *et al*. Sub-10 nm Fe_3O_4@$Cu_{2-x}S$ core–shell nanoparticles for dual-modal imaging and photothermal therap. *J. Am. Chem. Soc*. **135**, 8571–8577 (2013).

S2. Cui, C. *et al*. Organic semiconducting nanoparticles as efficient photoacoustic agents for lightening early thrombus and monitoring thrombolysis in living mice. *ACS Nano* **11**, 3298–3310 (2017).

S3. Kang, Y., Zhou, L., Li, X. & Yuan, J. *β*-Cyclodextrin-modified hybrid magnetic nanoparticles for catalysis and adsorption. *J. Mater. Chem*. **21**, 3704–3710 (2011).
